# The inverse-*trans*-influence in tetravalent lanthanide and actinide *bis*(carbene) complexes

**DOI:** 10.1038/ncomms14137

**Published:** 2017-02-03

**Authors:** Matthew Gregson, Erli Lu, David P. Mills, Floriana Tuna, Eric J. L. McInnes, Christoph Hennig, Andreas C. Scheinost, Jonathan McMaster, William Lewis, Alexander J. Blake, Andrew Kerridge, Stephen T. Liddle

**Affiliations:** 1 School of Chemistry, The University of Manchester, Oxford Road, Manchester M13 9PL, UK; 2 EPSRC National UK EPR Facility, School of Chemistry and Photon Science Institute, The University of Manchester, Oxford Road, Manchester M13 9PL, UK; 3 Helmholtz-Zentrum Dresden-Rossendorf, Institute of Resource Ecology, Bautzner Landstrasse 400, D-01314 Dresden, Germany; 4 The Rossendorf Beamline, ESRF, BP 220, F-38043 Grenoble, France; 5 School of Chemistry, University of Nottingham, University Park, Nottingham NG7 2RD, UK; 6 Department of Chemistry, Lancaster University, Lancaster LA1 4YB, UK

## Abstract

Across the periodic table the *trans*-influence operates, whereby tightly bonded ligands selectively lengthen mutually *trans* metal–ligand bonds. Conversely, in high oxidation state actinide complexes the inverse-*trans*-influence operates, where normally *cis* strongly donating ligands instead reside *trans* and actually reinforce each other. However, because the inverse-*trans*-influence is restricted to high-valent actinyls and a few uranium(V/VI) complexes, it has had limited scope in an area with few unifying rules. Here we report tetravalent cerium, uranium and thorium *bis*(carbene) complexes with *trans* C=M=C cores where experimental and theoretical data suggest the presence of an inverse-*trans*-influence. Studies of hypothetical praseodymium(IV) and terbium(IV) analogues suggest the inverse-*trans*-influence may extend to these ions but it also diminishes significantly as the 4*f* orbitals are populated. This work suggests that the inverse-*trans*-influence may occur beyond high oxidation state 5*f* metals and hence could encompass mid-range oxidation state actinides and lanthanides. Thus, the inverse-*trans*-influence might be a more general *f*-block principle.

## Abstract (web summary)

The inverse-*trans*-influence has been shown to operate in high oxidation state actinide complexes. Here, the authors report tetravalent cerium, uranium and thorium *bis*(carbene) complexes with trans C=M=C cores where experimental and theoretical data also suggest the presence of an inverse-*trans*-effect.

The *trans*-influence is a long-established, well-documented concept of broad relevance across inorganic chemistry^1^,^2^. This thermodynamic ground-state phenomenon classically occurs in square-planar and *pseudo*-octahedral d-block complexes where tightly bonded ligands selectively lengthen mutually *trans* metal–ligand bonds. The *trans*-influence is fundamentally important and underpins the *trans*-effect^3^, a kinetic rate effect where the order of substitution of ligands at a metal centre can be controlled; this is a key parameter to control, for example, the syntheses of *cis-* or *trans*-[PtCl_2_(NH_3_)_2_], whose isomerism is important regarding cancer treatment^4^. Although the bonding of lanthanide(III) and low/mid oxidation state early actinide ions is considered more ionic than in the d-block, there are crystallographic, and in some instances computationally supported, examples of complexes where metrical parameters are consistent with the presence of the *trans*-influence^5^,^6^,^7^,^8^,^9^,^10^,^11^,^12^,^13^,^14^,^15^,^16^.

In high oxidation state actinide complexes the opposite phenomenon of the inverse-*trans*-influence (ITI) can be found^17^,^18^,^19^,^20^,^21^. Here, strongly donating ligands that normally adopt *cis* orientations to avoid destabilizing the respective metal–ligand bonds via the *trans*-influence in fact reside *trans* to one another and even mutually reinforce each other. The classical, dominant example of the ITI is the uranyl(VI) dication, {UO_2_}^2+^, that adopts a *trans-*linear geometry and is chemically robust because of strong, ITI-strengthened uranium–oxygen bonds^20^. Indeed, linear *trans*-dioxo actinyls {AnO_2_}^n+^ (An=U, Np, Pu) are well known and prevalent, but *trans-*dioxos in the d-block are unusual and require strong equatorial σ-donor ligands to weaken the metal-oxo linkages sufficiently to enable them to reside mutually *trans*. Two isostructural complexes that demonstrate the *trans*-influence and the ITI are [MoO_2_Cl_2_(OPPh_3_)_2_] (**I**)^22^ and [UO_2_Cl_2_(OPPh_3_)_2_] (**II**)^23^, respectively (Fig. 1); in the former the oxos are *cis*, whereas in the latter they are *trans*. The ITI often plays a structure-dictating role, but this is not a criterion that must be met to make invoking the ITI valid; there are examples of high valent uranium complexes where ligands are constrained by their own architecture such that they have no choice but to place donor groups *trans* to a strongly donating ligand like a nitride or oxo, but despite this they present very short metal–ligand distances despite their unfavourable bonding situation. For example, in the complexes [U(Tren^TIPS^)(E)] [Tren^TIPS^=N(CH_2_CH_2_NSiPr^i^_3_)_3_; E=N (**III**), O (**IV**)] (Fig. 1)^24^,^25^ the U–N_amine_ distances are short at 2.465(5) and 2.482(6) Å, respectively, despite being *trans* to nitride and oxo ligands, whereas such U–N_amine_ distances are normally 2.5–2.7 Å (ref. 26). In these systems there is no ITI structure-dictating role, and as part of a polydentate Tren-ligand with minimal reorganization energy^27^ the Tren amines are forced to be unfavourably *trans* to a nitride or oxo^28^, but the U–N_amine_ distances are short, not long, that credibly invokes the ITI.

The origin of the ITI is complex, but is in part rationalized on the basis that in high oxidation state early actinides 6*p* orbitals are semi-core and transfer electron density to vacant 5*f* orbitals, creating an electron hole that is compensated for by additional donation of electron density from *trans* ligands^17^,^18^,^19^,^20^,^21^. For many years the ITI was limited to uranyl(VI) complexes^20^ or structurally analogous complexes such as [UOCl_5_]^−^ (ref. 29), but in recent years a limited number of uranium(V) and (VI) ITI complexes have emerged^24^,^25^,^30^,^31^,^32^,^33^. The unifying theme has been high oxidation state (V or VI) metal complexes combined with hard, polarizing, charge-loaded oxo, imide and nitride ligands. Because it is limited to high oxidation state early actinides, the question of whether the ITI is a niche concept or in fact has a broader underpinning role for the *f*-block has remained unanswered for around a quarter of a century in an area with few unifying rules.

When considering if the ITI could have a broader basis, it would have to be demonstrated to operate over a larger range of oxidation states, and be expanded beyond actinides to include the lanthanides. The IV oxidation state is the logical next step to take in terms of the general synthetic availability of uranium and thorium complexes, as the only two actinides that can be routinely handled without specialist facilities, and also because a IV oxidation state opens the door to extend this concept to the lanthanides; cerium has an accessible IV oxidation state under normal conditions, presenting the opportunity to compare cerium, uranium and thorium together^34^,^35^. Although hard, formally di- and trianionic oxygen and nitrogen ligands have so far exclusively supported the ITI with high oxidation state metals, by moving to a mid-range oxidation state a softer, isoelectronic dianionic carbon-based ligand might be arguably desirable to approximately maintain the relative energy matching of frontier metal and ligand orbitals, and we note that the only examples of uranium and even thorium in the +2 oxidation state under ambient conditions are stabilized by carbon-based ligands^36^,^37^,^38^. Indeed, carbon should be a good ligand for the ITI more generally because of its generally high-lying frontier orbitals compared with uranium^31^. However, a paucity of synthetically accessible families of complexes where the metal can be varied in a common mid-range oxidation state has limited testing the above hypothesis.

Here, we report the realization of our aim by the synthesis of cerium, uranium and thorium *bis*(carbene) complexes that exhibit linear C=M=C cores supported by the BIPM^TMS^ ligand [BIPM^TMS^={C(Ph_2_PNSiMe_3_)_2_}^2−^]. Although the C=M=C units are *trans* and thus would conventionally be expected to present long M=C distances, they in fact exhibit exceedingly short M=C distances, and for cerium among the shortest experimental Ce-C distance on record. Theoretical calculations reveal that when the *pseudo*-core 5*p* (cerium) or 6*p* (uranium or thorium) orbitals are isolated from the valence manifold the M=C distances increase. Taken together with the short M=C distances, and considering that they are disposed *trans*, this suggests that the ITI may extend beyond high oxidation state 5*f* metals to operate in mid-range oxidation state *f-*element metal complexes with appropriate ligand matching. Investigations of hypothetical praseodymium(IV) and terbium(IV) analogues suggest similar but increasingly diminished ITI phenomena.

## Results

### Synthesis

Treating the cerium(III) carbene-methanide complex [Ce(BIPM^TMS^)(BIPM^TMS^H)] (**1Ce**)^39^ with benzyl potassium and 18-crown-6 ether (18C6) in tetrahydrofuran (THF) gives the yellow cerium(III) *bis*(carbene) complex [Ce(BIPM^TMS^)_2_][K(18C6)(THF)_2_] (**2Ce**) in 52% yield (Fig. 2). Although cerium(IV) is regarded as a difficult oxidation state to access in an organometallic context because cerium(IV) is oxidizing and organometallic ligands are reducing, we find that oxidation of **2Ce** can be straightforwardly accomplished by AgBPh_4_ to give the green cerium(IV) *bis*(carbene) [Ce(BIPM^TMS^)_2_] (**3Ce**) in 43% yield after work-up and recrystallization (Fig. 2). The oxidation of **2Ce** to **3Ce** is so favourable that even small traces of dry air will effect oxidation. This suggests that the two carbenes together are well suited to stabilizing cerium(IV) and producing a robust C=Ce=C unit, cf, the stability of the ITI-stabilized uranyl O=U=O dication. The corresponding uranium and thorium *bis*(carbene) complexes [M(BIPM^TMS^)_2_] (M=U, **3U**; Th, **3Th**) were prepared by a different methodology (Fig. 2). The mono(carbene) dichloride complexes [M(BIPM^TMS^)Cl_3_Li(THF)_2_] (M=U, **4U**; Th, **4Th**)^40^,^41^ were converted to the corresponding dialkyls [M(BIPM^TMS^)(CH_2_SiMe_3_)_2_] (M=U, **5U**; Th, **5Th**)^42^; subsequent thermolysis with BIPM^TMS^H_2_ gave **3U** and **3Th** in 75% and 52% yields as brown and colourless crystals, respectively, after work-up.

### Characterization data

The ^1^H nuclear magnetic resonance (NMR) spectra of **3Ce** and **3Th** span 0–10 p.p.m. and are characteristic of diamagnetic complexes, whereas that of paramagnetic **3U** spans ±33 p.p.m. The ^31^P NMR spectra of **3Ce**, **3U** and **3Th** exhibit resonances at −13.7, −219.7 and 6.3 p.p.m., respectively; the ^31^P NMR resonance for **3Ce** compares well with that of [Ce(BIPM^TMS^)(ODipp)_2_] (−10.2 p.p.m., Dipp=2,6-diisopropylphenyl)^43^, and those of **3U** and **3Th** are typical of such complexes^41^,^44^. The ^13^C{^1^H} NMR carbene resonances for **3U** and **3Th** could not be located, even utilizing ^13^C-^31^P 2D NMR techniques, but the equivalent carbenes in **3Ce** were observed at 343.5 p.p.m. (*J*_PC_=170 Hz); this is more deshielded than that of [Ce(BIPM^TMS^)(ODipp)_2_] (324.6 p.p.m., *J*_PC_=149 Hz)^43^, possibly suggesting that the carbenes in **3Ce** are donating more strongly to cerium than in [Ce(BIPM^TMS^)(ODipp)_2_] despite their *trans* arrangement. This might be expected for an ITI, and is also within the 200–400 p.p.m. range of covalent transition metal carbenes rather than that observed for ionic yttrium(III) analogues (10–40 p.p.m.)^44^.

As expected for a ^1^S_0_ 4*f*^0^ cerium(IV) ion, the ultraviolet/visible/near-infrared (UV/Vis/NIR) spectrum of **3Ce** exhibits no absorbances in the NIR region where *f*–*f* or transitions associated with multiconfigurational character might occur (Supplementary Methods). Two absorptions (Fig. 3a), the broadness of which is a defining feature in many cerium(IV) complexes^45^, are observed in the visible region at 17,000 and 24,700 cm^−1^ (ε=4,895 and 14,387 M^–1^ cm^–1^, respectively), and the latter absorbance is responsible for the green colour of **3Ce**. In order to understand the electronic transitions responsible for the green colour of **3Ce** we modelled the spectrum using time-dependent density functional theory (TD-DFT) calculations at the statistical average of orbital potentials (SAOP)/zeroth order regular approximation (ZORA)/triple zeta basis set with one polarization function (TZP) level and the profile of the experimental spectrum is reproduced well by these calculations (Supplementary Fig. 1); the calculated absorption bands at 17,000 and 24,700 cm^−1^ are principally composed of ligand-to-metal charge-transfer from Ce=C π- and σ-combinations to cerium(IV) 4*f* orbitals. The UV/Vis/NIR spectrum of **3U** is characterized by weak (ε<80 M^–1^ cm^–1^) absorptions over the range 5,000–20,000 cm^−1^ that are characteristic of the ^3^H_4_ electronic manifold of the 5*f*^2^ uranium ion (Supplementary Fig. 2), whereas for **3Th** the spectrum is featureless in the visible and NIR regions as expected for its 6*d*^0^5*f*^0^ nature.

The room temperature cyclic voltammogram of **3Ce** reveals a well-resolved, quasi-reversible single redox process at *E*_1/2_=−1.63 V versus [Fe(η^5^-C_5_H_5_)_2_]^0/+^ assigned as the Ce^IV^/Ce^III^ redox couple (Fig. 3b; for full scan see Supplementary Fig. 3). The Ce^IV^/Ce^III^ redox couple is known to vary widely as a function of ligand environment^46^, and the reduction potential observed for **3Ce** is towards the negative end of reported values, suggesting that the *bis*(carbene) environment stabilizes cerium(IV), reflecting the facile oxidation of **2Ce** to **3Ce**. Under the same conditions, no redox processes were observed for **3U** or **3Th** in the accessible solvent window.

Powdered samples of **3Ce**, **3U** and **3Th** were studied by variable temperature SQUID (superconducting quantum interference device) magnetometry (Fig. 3c and Supplementary Fig. 4). Complex **3U** has a *χ*T value of 0.95 cm^3^ K mol^−1^ at 298 K (equivalent to 2.77 *μ*_B_, in agreement with an Evans method solution magnetic moment of 2.61 *μ*_B_) that decreases below 30 K to a value of 0.4 cm^3^ K mol^−1^ at 2 K. Unlike typical magnetization behaviour of uranium(IV) complexes generally (Supplementary Fig. 5), complex **3U** retains a high *χ*T value over most of the temperature range and the low-temperature *χ*T value is much higher than expected from temperature-independent paramagnetism alone. This suggests that the crystal field of two strongly donating axial ligands is sufficient to stabilize low-lying paramagnetic states (consistent with low-temperature magnetization data) separated widely from higher energy states (assuming a ^3^H_4_ ground term, a strongly axial field would stabilize the |*m*_J_|=4 non-Kramers doublet), and hence even at low temperatures the complex is paramagnetic. This phenomenon has been observed before in uranium(IV) complexes with strongly donating, multiply bonded axial ligands^47^,^48^,^49^,^50^,^51^,^52^. Complexes **3Ce** and **3Th** are diamagnetic, and the essentially nil or small negative *χ*T slope for the latter rule out any temperature-independent paramagnetism behaviour, suggesting there is no multiconfigurational ground character in **3Ce**, and this is also consistent with the absence of low energy absorptions in the optical spectrum. At low temperatures, complex **3Ce** has an insignificant *χ*T value (0.003 cm^3^ K mol^−1^ at 2 K) and X- and Q-band electron paramagnetic resonance (EPR) spectra are completely silent that contrasts with 4*f*^1^ **2Ce** that exhibits strong EPR features characteristic of cerium(III) (Supplementary Figs 6 and 7).

The characterization data for **3Ce**, **3U** and **3Th** support their IV oxidation state formulations. To unequivocally confirm that **3Ce** is a ^1^S_0_ complex we subjected it to X-ray absorption spectroscopy techniques. The X-ray photoemission spectroscopy (XPS) of **3Ce** is weak because the cerium is a small component of the 151-atom structure, but it exhibits a spectrum characteristic of cerium(IV) in the energy range 870–930 eV (Supplementary Fig. 8). The X-ray absorption near-edge spectroscopy (XANES) of **3Ce** reveals two absorptions characteristic of cerium(IV)^53^, (Fig. 3d), and when all characterization data are taken together the self-consistent picture that emerges is that **3Ce** is a closed-shell singlet cerium(IV).

### Solid-state structures

The solid-state structures of **3Ce**, **3U** and **3Th** were determined by single crystal X-ray diffraction and were found to be essentially isostructural. Complex **3Ce** is illustrated in Fig. 4 and further details of that and all other structurally determined complexes in this study can be found in Supplementary Figs 9–14 and Supplementary Tables 1 and 2. In each case, *pseudo*-octahedral metal centres present with mutually *trans* carbenes (**3Ce**, 176.98(7)°; **3U**, 177.5(2)°; **3Th**, 176.21(8)°) with deviations from the octahedral ideal because of the BIPM^TMS^ N-M-N bite angles resulting in the imino nitrogen atoms residing above and below the hypothetical equatorial plane. The carbenes adopt essentially planar T-shaped geometries with sum of angles spanning the range 357.23(18) to 360.00(15)° and the metal-BIPM^TMS^ 6-membered chelate rings are essentially planar contrasting with some BIPM^TMS^ complexes where the carbene can be distinctly pyramidal^44^, including [Ce(BIPM^TMS^)(ODipp)_2_]^43^. In **3Ce**, **3U** and **3Th**, the two BIPM^TMS^ ligands are disposed essentially orthogonally to one another, with dihedral angles between the two N-M-N planes of 92.5(2), 92.2(4) and 91.4(2)°, respectively. The Ce1-C1 and Ce1-C32 distances in **3Ce** (2.385(2) and 2.399(3), respectively) are exceedingly short, >0.2 Å shorter than the corresponding distances in **2Ce**, and ∼0.04 Å shorter than the Ce=C distances in [Ce(BIPM^TMS^)(ODipp)_2_]^43^ and the special case of cerium confined within an endohedral fullerene^54^. Significantly, considering they are *trans*, the Ce-C bond distances in **3Ce** are the shortest experimentally determined Ce-C distances to date in discrete molecular compounds, being surpassed only by short (2.247(17)-2.334(15) Å) cerium–carbon distances in periodic ethynediide–halide clusters^55^,^56^,^57^,^58^,^59^. Short molecular Ce-C distances have been found in theoretical models of experimentally unknown CeCH_2_^+^ and [Cp_2_CeCH_2_] that are sterically unimpeded and, in the case of the former, benefit from the reduced electronic repulsion from a net positive charge^60^,^61^.

The U1-C1 and U1-C32 distances in **3U** (2.410(6) and 2.421(6) Å, respectively) are statistically equivalent to the corresponding distances in **3Ce** (ionic radii of Ce^IV^=0.87 Å versus U^IV^=0.89 Å)^62^. However, the Th1-C1 and Th1-C32 distances in **3Th** (2.514(3) and 2.516(3) Å, respectively) are ∼0.05 Å longer than would be predicted purely based on the increase in ionic radius of Th^IV^ (0.94 Å)^62^. The short M=C bond lengths in **3Ce**, and to some extent **3U**, are all the more notable for the fact they are *trans*, and suggest that an ITI, rather than *trans*-influence, may be operating.

### Reactivity

To give experimental support to the formulation of **3Ce**, **3U** and **3Th** as carbene complexes, we examined their reactivity towards PhCHO. In all cases the Wittig-alkene product PhC(H)=C(PPh_2_NSiMe_3_)_2_ was formed in essentially quantitative yield. Although **3Th** reacts too quickly to be monitored (reaction complete in <5 min), and **3U** reacts quickly (>80% consumed in 15 min) and is paramagnetic, hence reliable data could not be extracted from questionable NMR integrations, was found to be amenable to a full study (Supplementary Figs 15–17). The reaction of **3Ce** with two equivalents of PhCHO was fitted to second-order kinetics overall (first order with respect to both **3Ce** and PhCHO) with *k* (298 K)=1.28 × 10^−4^±0.255 × 10^−4^ mol^−1^ dm^3^ s^−1^. Eyring and Arrhenius analyses yielded Δ*H*^‡^=+37.2±2 kJ mol^–1^ and Δ*S*^‡^=–194.4 ±6 J mol^–1^ K^–1^, affording Δ*G*^‡^=+95.2±2 kJ mol^–1^ (298 K) and *E*_a_=+40.0±2 kJ mol^–1^. These data are consistent with the reaction conditions and the metallaoxetane intermediates formed from a double [2+2]-cycloaddition/bond metathesis reaction to produce CeO_2_ and PhC(H)=C(PPh_2_SiMe_3_)_2_. This reactivity is characteristic of covalent early metal carbenes^44^ and contrasts with more ionic yttrium(III)-methanediide BIPM^TMS^ complexes that activate aryl C–H bonds of carbonyl compounds^63^. We note that the order of reactivity of **3Ce**, **3U** and **3Th** with PhCHO is **3Th**>**3U**>**3Ce**; this is principally consistent with the ionic radii of the metals but is also consistent with the increasing level of covalency in the M=C bonds of **3Ce**>**3U**>**3Th** suggested by our calculations.

### Theoretical calculations

To probe the electronic structures of **3Ce**, **3U** and **3Th**, we undertook DFT calculations on their full structures. The geometry-optimized calculations match closely the experimentally determined structures within 0.05 Å and 2° (Supplementary Tables 3–11), and the TD-DFT calculations model the experimentally determined electronic absorption spectrum of **3Ce** well. We thus conclude that the theoretical models provide a qualitative description of the electronic manifolds of **3Ce**, **3U** and **3Th**. Although the analysis that follows shows that the dominant feature of the metal–carbon interactions in **3Ce**, **3U** and **3Th** is electrostatic bonding, covalent contributions are present and the discussion focusses on this latter aspect. For **3Ce**, **3U** and **3Th**, the calculated MDC_q_ metal charges are 1.99, 2.77 and 2.48, respectively, and the MDC_q_ carbene charges are −1.79/−1.80, −2.03/−2.06 and −1.95/−2.01, respectively. Although care must be taken when analysing calculated charges, they are indicative of metal(IV) ions^44^,^64^ and that perhaps the cerium ion in **3Ce** is better matched to receiving electron donation from the ligands than the uranium and thorium ions in **3U** and **3Th**. However, the calculated MDC_m_ spin density of −2.27 for the uranium ion in **3U** is certainly consistent with charge donation from the BIPM^TMS^ ligand to uranium and also with its 5*f*^2^ uranium(IV) formulation.

Inspection of the Kohn Sham frontier orbitals of **3Ce**, **3U** and **3Th** (Supplementary Figs 18–20) clearly shows twofold bonding interactions between each carbene and the respective metal. Each M=C bond is polarized, however, as evidenced by M=C Nalewajski–Mrozek bond orders averaging 1.10, 1.30 and 0.73 for **3Ce**, **3U** and **3Th**, respectively. For comparison, the Ce=C bond order in [Ce(BIPM^TMS^)(ODipp)_2_] is also 1.1 (ref. 43), whereas uranium(IV)-BIPM complexes typically have U=C double and U–C single bond orders of ∼1.4 and ∼0.5, respectively^44^,^64^, and thorium is polarized with Th=C bond orders of ∼0.7 (ref. 64). Therefore, the data presented here fit the overall literature trends.

A clearcut view of the M=C bonding in **3Ce**, **3U** and **3Th** from the Kohn Sham frontier orbitals is precluded because of the delocalized nature of molecular orbital calculations. We therefore turned to natural bond orbital (NBO) analysis (Supplementary Figs 21 and 22) that is suited to the treatment of chemical bonding in molecular complexes. For **3Ce**, the Ce=C σ-bonds are composed of ∼13% cerium and 87% carbon character; in each case the cerium contribution is ∼46% 5*d* and ∼53% 4*f*, with the remaining ∼1% being 6*s*. For the Ce=C π-bonds, cerium contributes only ∼8% to these bonds and the cerium component is predominantly 4*f* character (∼80%) with a modest 5*d* component (∼19%), with the remainder being of 5*p* character. These data compare well with those of [Ce(BIPM^TMS^)(ODipp)_2_]^42^, and indeed there is growing evidence that cerium(IV) is suited to utilizing its 4*f* orbitals in bonding to ligands^43^,^53^,^65^,^66^. This may be important, because a study on lanthanide(III) chalcogenide complexes found a *trans*-influence where the lanthanide utilizes predominantly *d* orbitals^12^; in contrast the metal(IV) complexes, certainly for cerium and uranium, are deploying predominantly *f* orbitals, in line with the general theory of the ITI^17^,^18^,^19^,^20^,^21^. For **3U**, the U=C σ-bonds are composed of ∼14.5% uranium character and ∼85.5% carbon character. As for **3Ce**, the 5*f*:6*d* contributions of **3U** are well balanced at ∼51:47%, with the remainder of 7*s* character. The U=C, π-bond is ∼14:86% uranium/carbon and, like for cerium, is within the uranium component principally 5*f* (∼86%) with modest 6*d* (∼13%), with the remaining ∼1% being 6*p* character. For **3Th**, NBO does not return any Th=C interactions, suggesting that the Th=C bonding is highly ionic.

To better understand the nature of the M=C interactions in these compounds we turned to complete/restricted active space self-consistent field (CASSCF/RASSCF) methodologies that allows us to consider electron correlation through a rigorous configuration-interaction-based approach to directly compare open- and closed-shell compounds. The computational cost of such calculations required truncation and symmetrization of the experimentally determined structures to produce tractable models, but by retaining key structural motifs near identical electronic structures are obtained (Supplementary Tables 12–14).

RASSCF calculations were employed in order to identify an appropriate active space for each system. Because of the computational expense of such calculations, RAS1, RAS2 and RAS3 subspaces were constrained to consist of 12, 7 and 12 orbitals, respectively. The 7 RAS2 orbitals comprise the 4*f*/5*f* manifold, whereas the RAS1 and RAS3 orbitals account for orbitals with significant C/N 2*s* and 2*p* character and natural orbitals whose occupation numbers most deviate from integer values. This active space ensured that all M=C and M–N interactions were accurately modelled. State-averaged RASSCF calculations indicated a degenerate ^3^B_1_/^3^B_2_ ground state in the **3U** model complex, corresponding to a state of E symmetry in the full idealized D_2d_ point group. The natural orbital occupation numbers resulting from these calculations allowed complete active spaces to be defined. Subsequent CASSCF calculations correlated 8 electrons in 8 orbitals in the cases of **3Ce** and **3Th** model complexes, and 10 electrons in 12 orbitals in the case of the U complex.

CASSCF calculations revealed that all complexes are dominated by M(IV) configurations, (Fig. 5), contributing 96.0%, 96.0% and 95.4% to the ground state wavefunctions of the **3Ce**, **3U** and **3Th** model complexes, respectively. Maximum deviations from integer values in natural orbital occupations were 0.026, 0.021 and 0.026 for the **3Ce**, **3U** and **3Th** model complexes, respectively, indicating at most weak multiconfigurational character. The resultant electronic structure is almost identical to that obtained from the RASSCF simulations, and the dominant M(IV) character in all systems is commensurate with all experimental measures. Notably, as shown in Fig. 5, **3Ce**, **3U** and **3Th** show σ-bonding combinations that are strongly reminiscent of uranyl, a feature that also emerges from the DFT analysis that is consistent with an ITI in these complexes.

To further probe the covalent contribution to bonding in these compounds, we directly analyse the resultant electron densities via the quantum theory of atoms in molecules (QTAIM) and focus on two parameters: the delocalization index between two bonded atomic centres (δ) and the magnitude of the electron density at the bond critical point between the centres (*ρ*_BCP_). The delocalization index, formally a two-electron property of the system, is a measure of the number of electrons shared between two atoms, and is large when orbital mixing because of energetic near degeneracy between the two atoms is pronounced. In this sense, it probes similar properties to those given by orbital decomposition via NBO analysis or, experimentally, by X-ray absorption spectroscopy that probes orbital energy near degeneracy. On the other hand, *ρ*_BCP_ quantifies electronic charge concentration in the bond between two atoms. Combined, these measures give an indication as to whether orbital mixing leads to charge accumulation in the bonding region (and hence bond stabilization) and hence provide a more complete method for assessing bond covalency than orbital analysis alone (Supplementary Table 15)^53^.

QTAIM-derived atomic charges are more consistent than those obtained via NBO analysis, with *q*(Ce)<*q*(U)<*q*(Th). Carbene charges reflect this trend that therefore provides some evidence for a greater covalent interaction in the **3Ce** complex. Stronger evidence is provided by the delocalization indices, *δ*(M,C), that are notably larger for **3Ce** than either **3U** or **3Th**. Consideration of *ρ*_BCP_ demonstrates that this electron sharing corresponds to charge accumulation in the M=C bonding region: *ρ*_BCP_ follows a similar trend to *δ*(M,C), with the Ce and U complexes exhibiting significantly larger values than that of Th. Inspection of the ellipticity parameter *ε* for the M=C bonds reveals values that are consistent with an asymmetric distribution of electron density around the M=C bond comparable to those found for alkenes and [M(BIPM^TMS^)(ODipp)_2_]^43^, confirming that in **3Ce**, **3U** and **3Th** there is a M=C bonding interaction involving two electron pairs donated from a carbene to a metal.

### Probing the inverse-*trans*-influence

To probe whether the ITI is operating in **3Ce**, **3U** and **3Th**, we adopted the method of O’Grady and Kaltsoyannis^29^. Here, **3Ce**, **3U** and **3Th** were geometry optimized with a frozen core, up to 4*d* for **3Ce** and 5*d* for **3U** and **3Th** and with the *pseudo*-core 5 or 6*p* orbitals, respectively, either explicitly included as valence orbitals or placed in the frozen core. Although the ITI is a complex phenomenon that involves several factors, it is clear that p orbitals are involved in the ITI and that this method isolates the contributions that the *pseudo*-core 5/6*p* orbitals have on the bonding. Although the carbene ligands in **3Ce**, **3U** and **3Th** clearly exhibit polarized M=C bonds, the ITI is predominantly dependent on the charge and polarizing nature of the coordinated ligands^33^. To rule out coincidental systematic errors from a particular method, we examined the effect of varying the functional (BP86 versus PBE) and the basis set (normal all-electron basis set versus a normal frozen core up to 4 or 5*d* for **3Ce**, **3U**, and **3Th**, respectively) separately or simultaneously, and found no significant changes in equilibrium geometries. However, when the 5 or 6*p* orbitals for **3Ce**, **3U** and **3Th**, respectively, were additionally also placed in the frozen core, significant changes to the equilibrium geometries were observed in all cases. Specifically, the M=C distances lengthen by ∼0.05 Å when the relevant *p* orbitals are placed in the frozen core that are very similar shifts to those found previously for [MOX_5_]^−^ anions (M=U, Np; X=F, Cl, Br)^29^, and this represents the *p* orbital contributions to the ITI. It should be noted that, on inclusion of the relevant *p* orbitals into the frozen cores, the M–N_imino_ distances also elongate; however, the latter lengthen by only ∼0.02 Å, less than half that of the change to the M=C linkages. As expected, with *p* orbitals in the frozen cores the metal and carbene charges increase, indicating more polarized and presumably weakened interactions. For example, in **3Ce** the cerium and carbene charges rise from 1.83 and −1.71 when 5*p* orbitals are included in the valence region to 2.54 and −1.82 when 5*p* orbitals are placed in the frozen core. Inspection of the differences of the total energies of the geometry-optimized structures of **3Ce**, **3U** and **3Th** with the *p* orbitals in the frozen core or in the valence region yields energy differences of 12.5, 20.5 and 18.2 kcal mol^−1^ for **3Ce**, **3U** and **3Th**, respectively. This provides a qualitative bracketing of the stabilizing energy that the inclusion of the *pseudo*-core *p* orbitals in the bonding to a *bis*(carbene) ligand set provides, and compares well with the ITI of 6 kcal mol^−1^ calculated for a uranium(VI)-*mono*(oxo) unit in a tris(aryloxide) triazacyclononane complex^30^.

**Figure 1:**
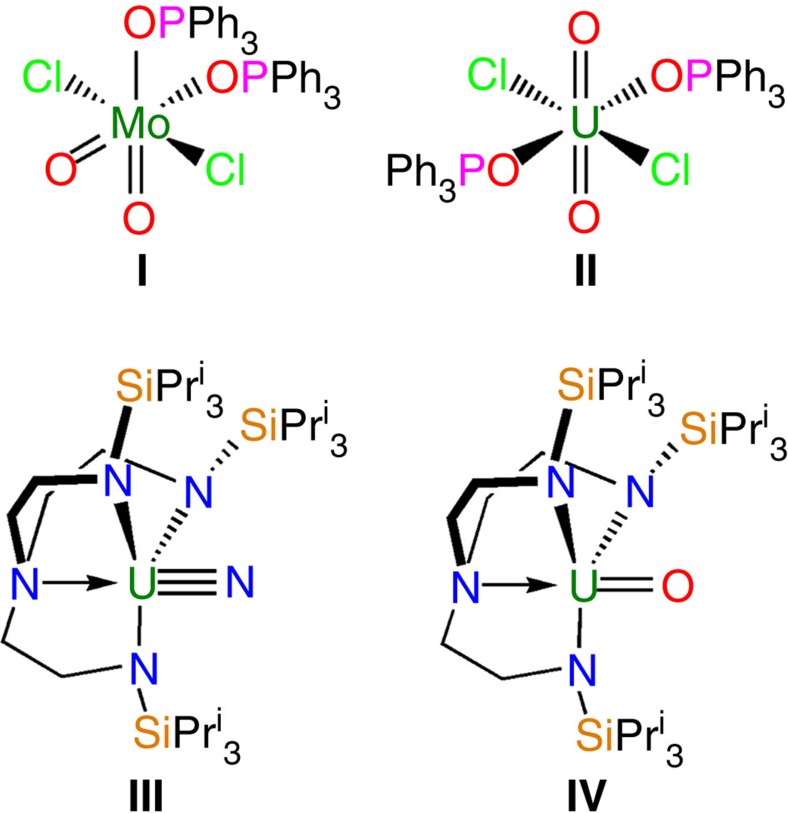
Selected literature examples where the *trans*-influence and inverse-*trans*-influence are invoked. Complexes **I** and **II** are identical except for the metal ion and the molybdenum complex exhibits *cis*-oxos whereas the uranium complex has *trans*-oxos, consistent with the *trans*-influence and inverse-*trans*-influence, respectively. Complexes **III** and **IV** both exhibit short uranium–amine distances despite being *trans* to hard, charge-loaded nitride and oxo ligands that is not consistent with the *trans*-influence but conversely invokes the inverse-*trans*-influence.

**Figure 2:**
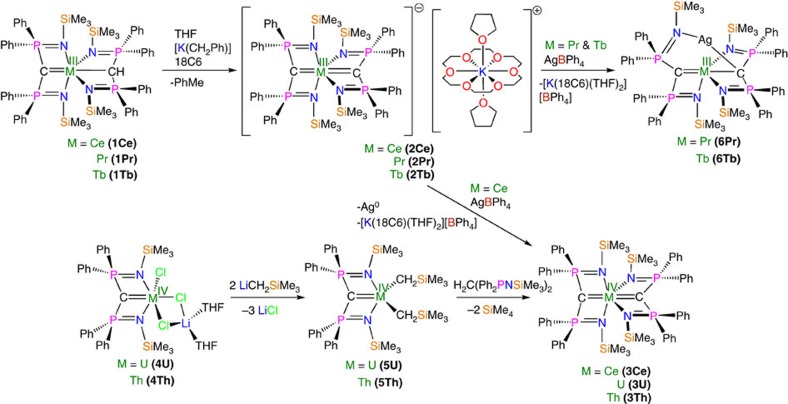
Synthesis of *bis*(carbene) complexes. The cerium(III) carbene–methanide complex **1Ce** reacts with benzyl potassium and 18C6 in THF to give the cerium(III) *bis*(carbene) separated ion pair complex **2Ce** with elimination of toluene. Complex **2Ce** is readily oxidized by silver tetraphenylborate to give the cerium(IV) *bis*(carbene) complex **3Ce** with elimination of elemental silver and potassium 18-crown-6 ether *bis*(THF) tetraphenylborate. The uranium and thorium carbene complexes **4U** and **4Th** are converted to the corresponding carbene dialkyl complexes **5U** and **5Th** by salt elimination with two equivalents of trimethylsilylmethyl lithium. Complexes **5U** and **5Th** react with BIPM^TMS^H_2_ by alkane elimination to give the uranium and thorium *bis*(carbene) complexes **3U** and **3Th**, respectively. Attempts to prepare the praseodymium(IV) and terbium(IV) analogues of **3Ce** resulted in elimination of potassium 18-crown-6 *bis*(THF) tetraphenylborate and insertion of silver into the *bis*(carbene) complexes to give **6Pr** and **6Tb**.

**Figure 3:**
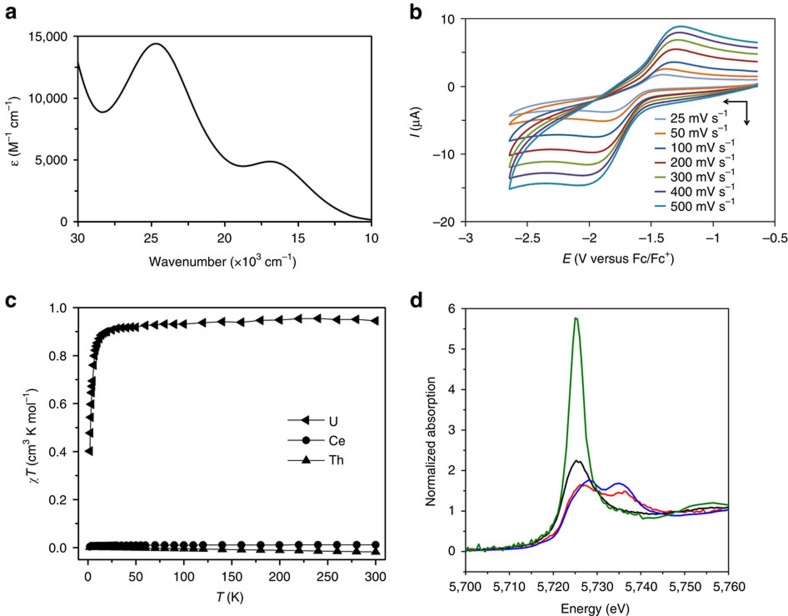
Spectroscopic and magnetic characterization data. (**a**) The UV/Vis/NIR spectrum of **3Ce**, illustrating the broad nature of the two principal transitions in the visible region. (**b**) Cyclic voltammogram of 0.2 mM **3Ce** in THF at selected sweep rates (0.1 M [N(Pr)_4_][BAr^F^_4_] supporting electrolyte, BAr^F^_4_=tetrakis(3,5-trifluoromethylphenyl)borate) versus [Fe(Cp)_2_]^0/+^ showing a single quasi-reversible redox process assigned to the Ce^IV^/Ce^III^ redox couple. (**c**) Variable temperature magnetic susceptibility data for **3Ce**, **3U** and **3Th** indicative of populated low-lying paramagnetic states for **3U** and diamagnetic, closed-shell assignments for **3Ce** and **3Th**. (**d**) Cerium L_III_-edge XANES spectrum of the cerium(IV) complex **3Ce** (red trace) in comparison with its cerium(III) precursor **2Ce** (black trace). As references, spectra of 0.01 M cerium(III) nitrate in water (green trace) and of cerium(IV) dioxide (blue trace) are given. The XANES spectra of **2Ce** and **3Ce** were recorded at 15 K and the references were recorded at 298 K.

**Figure 4:**
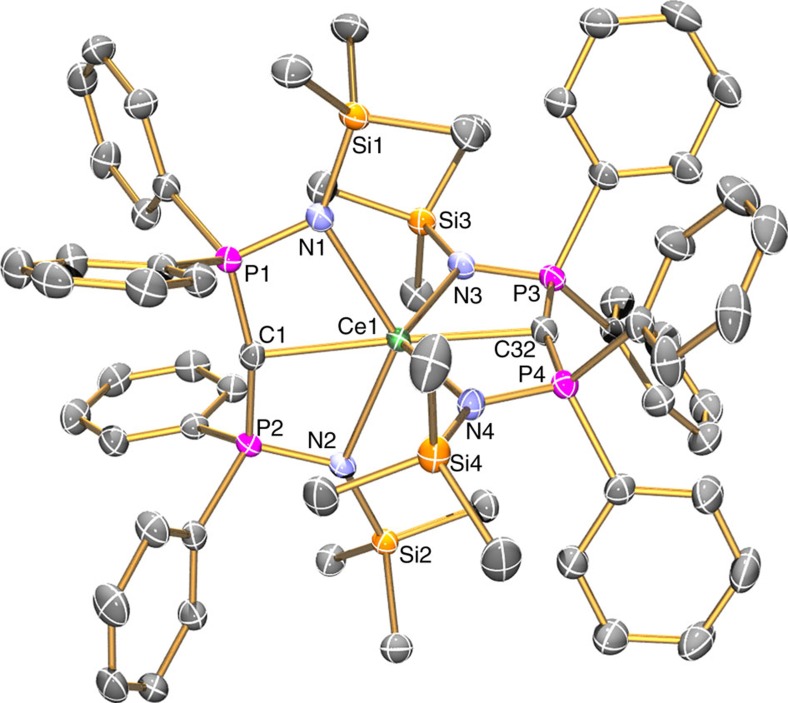
Molecular structure of 3Ce at 150 K with displacement ellipsoids set to 30%. Hydrogen atoms and lattice solvent are omitted for clarity. The structures of **3U** and **3Th** are very similar. Selected bond lengths (Å) and angles (°): C1-P1 1.664(2), C1-P2 1.664(2), C32-P3 1.665(5), C32-P4 1.663(4), P1-N1 1.6128(18), P2-N2 1.6174(19), P3-N3 1.6247(18), P4-N4 1.6202(18), Ce1-C1 2.385(2), Ce1-C32 2.399(3), Ce1-N1 2.4766(17), Ce1-N2 2.5122(17), Ce1-N3 2.4726(18), Ce1-N4 2.4966(16), P1-C1-P2 164.31(15), P3-C32-P4 163.61(14), N1-Ce1-N2 127.16(6), N3-Ce1-N4 127.36(6), C1-Ce1-C32 176.98(7).

**Figure 5:**
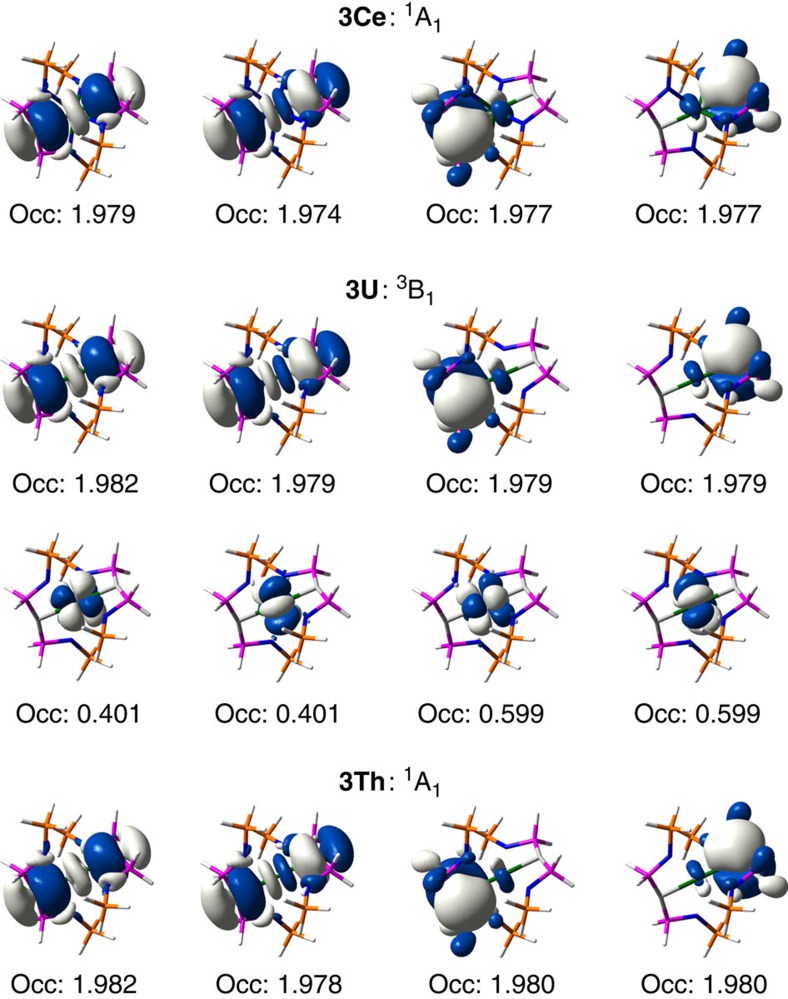
Selected CASSCF-calculated natural orbitals of truncated models of *bis*(carbene) complexes. Only strongly occupied M–C bonding orbitals and 5*f* orbitals are shown. Pronounced multiconfigurational character is present among the 5*f* orbitals in the truncated U complex, but the rest of the electronic manifold comprises orbitals with occupations close to integer values showing at best weak multiconfigurational character. All orbitals rendered at an isosurface of 0.02 atomic units (a.u.).

As **3Ce**, **3U** and **3Th** all appear to exhibit the ITI, we investigated the synthesis of the analogous praseodymium(IV) and terbium(IV) *bis*(BIPM) complexes **3Pr** and **3Tb**, respectively. We targeted these complexes because after cerium they have the next two lowest fourth ionization energies of all lanthanides^34^,^35^. Nevertheless, the fourth ionization energies of these two elements are still considerable, and we could not access **3Pr** and **3Tb** experimentally. Attempting AgBPh_4_-mediated oxidations of **2Pr** and **2Tb** (Fig. 1) results not in oxidation to give **3Pr** and **3Tb** but instead elimination of [K(18C6)(THF)_2_][BPh_4_] and isolation of [M(BIPM^TMS^)_2_Ag] (M=Pr, **6Pr**; M=Tb, **6Tb**); photolysis or electrochemistry experiments on **6Pr** and **6Tb** resulted in intractable decomposition products. However, although we could not prepare **3Pr** and **3Tb**, **3Ce**, **3U**, **3Th**, **2Pr** and **2Tb** provide experimentally calibrated benchmarks with which to provide confidence in the calculated hypothetical geometry-optimized structures of **3Pr** and **3Tb**. Inspection of the equilibrium geometries of **3Pr** and **3Tb** calculated with their respective 5*p* orbitals in-core and included in the valence regions reveals that the ITI persists but diminishes on moving from Ce to Pr to Tb (Supplementary Tables 16–19). Specifically, the Pr=C distances elongate by 0.02 Å when the 5*p* orbitals are placed in the frozen core, and the Pr-N distances elongate by only 0.006 Å. For Tb, the effect is significantly reduced, with a 0.007 Å elongation of the Tb=C distance when the 5*p* orbitals are placed in the frozen core and the Tb-N distances elongate by 0.006 Å. We conclude from these data that the ITI may, in principle, apply across the lanthanide(IV) series, but as 4*f* electron occupancy increases the ITI diminishes. This is consistent with the theory of the ITI as donation of (*n*)*p* electron density into the (*n*−1)*f* orbital manifold will become less favourable as the (*n*−1)*f* occupancy increases because of interelectronic repulsion^19^. Furthermore, the appearance of a trend suggests greater levels of *f*-orbital covalency that is supported by the characterization data more widely, whereas for lanthanide(III) systems when the *trans*-influence has been studied and principally *d*-orbital participation has been invoked then the *trans*-influence trend is uniform, suggestive of mainly ionic bonding character^12^. Thus, the observations of uniform *trans*-influence with *d*-orbital bonding for lanthanide(III) ions^12^ versus diminishing ITI for lanthanide(IV) ions where *f*-orbital bonding is principally invoked is internally consistent.

The delocalized nature of the molecular orbital approach makes the identification of key molecular orbitals involved in the ITI difficult^29^,^30^. Thus, inspection of individual molecular orbitals would not be expected to provide clear-cut information, as has proven to be the case even in highly symmetric complexes^29^. However, an examination of the electronic manifolds of **3Ce**, **3U** and **3Th** reveals a common molecular orbital that may be significant. All three complexes exhibit a molecular orbital at ∼−16.4 eV (**3Ce**, −16.483; **3U**, −16.485; **3Th**, −16.494 eV) ∼12 eV below the highest occupied molecular orbital (−2 for 5*f*^2^ **3U**). In each case contributions from the 2*s* orbitals of each carbene (normalized to 34%) and 5 or 6*p* orbitals for cerium or uranium and thorium, respectively (normalized to 5%) are found. Interestingly, a similar orbital is computed for **3Pr** at −16.476 eV, although here the carbon (normalized to 32%) and praseodymium (normalized to 2.5%) contributions are notably less well matched. In contrast, for **3Tb** the closest match is now a molecular orbital at −20.455 eV with normalized carbon and terbium contributions of 9% and 3%, respectively. We suggest that this molecular orbital that is common to **3Ce**, **3U**, **3Th** and **3Pr** but not **3Tb** may represent a signature in this instance of the ITI^19^,^20^,^29^.

## Discussion

We have prepared three new metal *bis*(carbene) complexes that contain linear C=M=C cores and the characterization data show these complexes to be unequivocally metal(IV) complexes and thus valid to compare with one another. For the cerium and uranium derivatives the M=C bonds are short, and for the former one of the shortest Ce-C bonds on record; that they are so short despite the fact that they are strongly donating dianions disposed *trans* with respect to one another suggests that the ITI is operating instead of the more classical *trans* influence. Theoretical calculations suggest the presence of an ITI in cerium, uranium and thorium derivatives, as removal of *pseudo*-core (*n*)*p* orbitals from the valence region in calculations consistently results in elongation of the M=C bonds by over twice that of the elongation of the M-imino bonds. Interestingly, the characterization data and theoretical data taken together suggest a consistent trend of covalency of **3Ce**∼**3U**>**3Th**. This work suggests that the ITI concept, first established a quarter of a century ago, may extend beyond high oxidation state 5*f* metals to now encompass mid-range oxidation state 5*f* actinides and 4*f* lanthanides. Calculations also suggest, however, that the ITI may diminish on moving from left to right in the lanthanide series and with increasing (*n*−1)*f* occupation number. Although an opposite trend may operate for the actinides^29^, it may be that the more diffuse 5*f* orbitals could tolerate occupancy more; however, thus far the radioactivity of those elements has precluded any detailed body of experimental work from being compiled and hence further work will be required to provide the necessary benchmarks with which to investigate this. The observations of uniform *trans*-influence with *d*-orbital bonding for lanthanide(III) ions^12^ versus the trend reported here of diminishing ITI for the lanthanide(IV) ions investigated, where *f*-orbital bonding is principally invoked, is gratifyingly internally consistent. Thus, the ITI might be a more general *f*-block principle.

## Methods

### General

Experiments were carried out under a dry, oxygen-free dinitrogen atmosphere using Schlenk-line and glove-box techniques. All solvents and reagents were rigorously dried and deoxygenated before use. Compounds were variously characterized by elemental analyses, electrochemistry, NMR, Fourier transform infrared spectroscopy (FTIR), EPR, XANES and UV/Vis/NIR electronic absorption spectroscopies, single crystal X-ray diffraction studies (Supplementary Data 1–11), Evans methods and SQUID magnetometry, and DFT, NBO, QTAIM, CASSCF and RASSCF computational methods.

### Synthesis of [Ce(BIPM^TMS^)_2_][K(18C6)(THF)_2_] (**2Ce**)

THF (15 ml) was added to a precooled (−78 °C) mixture of **1Ce** (1.41 g, 1.17 mmol) and [K(CH_2_Ph)] (0.15 g, 1.17 mmol). The resulting orange suspension was allowed to slowly warm to room temperature with stirring over 16 h to afford an orange solution. 18C6 (0.31 g, 1.17 mmol) in THF was added and stirred for a further 2 h. The solvent was removed *in vacuo* to afford an orange solid. The solid was washed with toluene to afford **2Ce** as a yellow powder. Yield: 2.31 g, 52%. Recrystallization of a small portion from toluene afforded yellow crystals of **2Ce** on storing at room temperature. Anal. Calcd for C_82_H_116_CeKN_4_O_8_P_4_Si_4_: C, 57.91; H, 6.87; N, 3.29%. Found: C, 57.65; H, 6.89; N, 3.09%. ^31^P{^1^H} NMR (C_6_D_6_, 298 K): δ 7.22 (CeC*P*_2_). FTIR *v*/cm^−1^ (Nujol): 1,350 (w) 1,302 (w), 1,077 (s), 961 (m), 846 (m), 771 (m), 743 (s), 695 (m), 633 (m), 522 (s). Magnetic moment (Evans method, THF, 298 K): *μ*_eff_=2.18 *μ*_B_.

### Synthesis of [Ce(BIPM^TMS^)_2_] (**3Ce**)

Toluene (15 ml) was added to a precooled (−78 °C) mixture of **2Ce** (1.64 g, 0.96 mmol) and [Ag(BPh_4_)] (0.41 g, 0.96 mmol). The resulting yellow suspension was allowed to warm to room temperature with stirring over 16 h to afford a green suspension. The suspension was filtered and volatiles were removed *in vacuo* to afford a green solid. Recrystallization from toluene (2 ml) afforded **3Ce** as green crystals. Yield: 0.52 g, 43%. Recrystallization of a small portion from pentane (3 ml) afforded green crystals suitable for single crystal X-ray diffraction analysis. Anal. Calcd for C_62_H_76_CeN_4_P_4_Si_4_: C, 59.42; H, 6.11; N, 4.47%. Found: C, 59.63; H, 6.15; N, 4.43%. ^1^H NMR (C_6_D_6_, 298 K): δ 0.95 (36H, s, NSi(C*H*_3_)_3_), 7.08 (24H, d, *p*/*o*-Ar-*H*), 7.24 (16H, m, *o*-Ar-*H*) p.p.m. ^13^C{^1^H} NMR (C_6_D_6_, 298 K): δ 6.41 (NSi(*C*H_3_)_3_), 127.26 (*m*-Ar-*C*), 129.78 (*o*-Ar-*C*), 132.52 (*p*-Ar-*C*), 140.50 (*i*-Ar-*C*), 343.48 (t, *J*_PC_=170.22 Hz, Ce*C*P_2_) p.p.m. ^31^P{^1^H} NMR (C_6_D_6_, 298 K): δ −13.65 (CeC*P*_2_) p.p.m. ^29^Si{^1^H} NMR (C_6_D_6_, 298 K): δ −2.17 (N*Si*(CH_3_)_3_) p.p.m. FTIR *v*/cm^−1^ (Nujol): 1,294 (w), 1,246 (w), 1,064 (s), 843 (m), 757 (w), 693 (w), 656 (m), 597 (w), 509 (m).

### Preparation of [U(BIPM^TMS^)(CH_2_SiMe_3_)_2_] (**5U**)

THF (10 ml) was added to a precooled (−78 °C) mixture of [U(BIPM^TMS^)(Cl)_3_(Li)(THF)_2_] (1.09 g, 1 mmol) and LiCH_2_SiMe_3_ (0.19 g, 2 mmol). The mixture was then allowed to slowly warm to room temperature with stirring over 6 h to afford a brown solution. Volatiles were removed *in vacuo* and the resulting brown solid was extracted with toluene (20 ml). Volatiles were removed *in vacuo* and the resulting brown solid was recrystallized from hexane (3 ml) and stored at −30 °C to afford **5U** as brown crystals. Yield 0.67 g (69%). Anal Calcd for C_38_H_60_N_2_P_2_Si_4_U: C, 47.22; H, 6.00; N, 2.98. Found: C, 46.53; H, 5.92; N, 2.97. The carbon is consistently low over five independently synthesized batches that we attribute to carbide formation. ^1^H NMR (C_6_D_6_, 298 K): δ −7.76 (s, 18H, Si(C*H*_3_)_3_), 0.41 (s, 18H, Si(C*H*_3_)_3_), 5.21 (m, 4H, Ar-*H*), 5.49 (br, 8H, Ar-*H*), 5.85 (br, 8H, Ar-*H*), 7.15 (br, 4H). IR *v*/cm^−1^ (Nujol): 1,403 (w), 1,305 (w), 1,248 (m, br), 1,108 (m), 1,019 (m, br), 836 (s, br). (Evans method, C_6_D_6_, 298 K): 2.45 *μ*_B_.

### Preparation of [U(BIPM^TMS^)_2_] (**3U**)

Toluene (15 ml) was added to a mixture of [U(BIPM^TMS^)(CH_2_SiMe_3_)_2_] (0.97 g, 1.00 mmol) and BIPM^TMS^H_2_ (0.56 g, 1.00 mmol) at room temperature. The resulting brown solution was stirred at 90 °C for 18 h, and then was allowed to cool to ambient temperature and filtered. All volatiles were removed from the filtrate to afford a brownish black solid that was washed by 5 ml of pentane at 0 °C to afford the product as a brown solid. Yield 0.81 g, 60%. Recrystallization of a small portion from a toluene/hexane mixture at 5 °C afforded brown crystals suitable for a single crystal X-ray diffraction study. Anal. Calcd for C_62_H_76_N_4_P_4_Si_4_U·C_6_H_14_: C 56.81; H 6.16; N 3.90. Found: C 56.44; H 6.16; N 3.89. ^1^H NMR (C_6_D_6_, 298 K): *δ* −33.88 (br, 36 H, NSi(C*H*_*3*_)_*3*_), 12.04 (s, 9 H, Ar-*H*), 13.76 (s, 17 H, Ar-*H*), 30.42 (br, 14 H, Ar-*H*). ^31^P{^1^H} NMR (C_6_D_6_, 298 K): *δ* −219.70 (br, UC*P*_2_) p.p.m. FTIR *ν*/cm^−1^ (Nujol): 1,958 (w), 1,450 (s), 1,334 (s), 1,281 (m), 1,245 (m), 1,178 (w), 1,155 (w), 1,103 (s), 1,035 (m), 745 (m), 669 (m), 628 (m), 514 (w), 489 (w). Magnetic moment (Evans method, C_6_H_6_ 298 K): *μ*_eff_=2.61 *μ*_B_.

### Preparation of [Th(BIPM^TMS^)(CH_2_SiMe_3_)_2_] (**5Th**)

Li_2_BIPM (1.71 g, 3 mmol) in THF (10 ml) was added to a precooled (−78 °C) suspension of ThCl_4_(THF)_3.5_ (1.88 g, 3 mmol) in THF (10 ml). The pale yellow reaction mixture was stirred at −78 °C for 30 min and at room temperature for 2 h. After which the mixture was cooled to −78 °C again, and LiCH_2_SiMe_3_ (0.56 g, 6 mmol) in THF (10 ml) was added. The resulted pale yellow solution was kept at −35 °C for 12 h and then all volatiles were removed *in vacuo* to afford a yellow oil that was extracted by *i*-hexane (3 × 10 ml). All volatiles were removed *in vacuo* from the filtrate to afford the product as a pale yellow solid. Yield: 2.17 g, 75%. Recrystallization of a small portion from *i-*hexane (3 ml) at −30 °C afforded colourless crystals suitable for single crystal X-ray diffraction analysis. Anal. Calcd for C_39_H_60_N_2_P_2_Si_4_Th: C, 48.63; H, 6.28; N, 2.91%. Found: C, 48.70; H, 6.39; N, 2.65%. ^1^H NMR (C_6_D_6_, 298 K): *δ* −0.01 (s, 4 H, ThC*H*_*2*_), 0.22 (s, 18 H, CH_2_Si*Me*_*3*_ or NSi*Me*_*3*_), 0.43 (s, 18 H, CH_2_Si*Me*_*3*_ or NSi*Me*_*3*_), 6.98 (br, 12 H, p- and o-Ar-*H*), 7.59 (br, 8 H, m-Ar-*H*) p.p.m. ^13^C{^1^H} NMR (C_6_D_6_, 298 K) *δ* 2.74 (s, CH_2_Si*Me*_*3*_ or NSi*Me*_*3*_), 4.54 (s, CH_2_Si*Me*_*3*_ or NSi*Me*_*3*_), 73.75 (t, ^1^*J*_PC_=159 Hz, Th*C*P_2_), 92.59 (s, Th*C*H_2_), 130.18 (s, o- and p-Ar*C*), 131.13 (t, ^3^*J*_PC_=5.6 Hz, m-Ar*C*), 137.79 (t, ^1^*J*_PC_=50.3 Hz, ipso-Ar*C*) p.p.m. ^31^P NMR (C_6_D_6_, 298 K): *δ* 5.81 (s) p.p.m. ^29^Si{^1^H} NMR (C_6_D_6_, 298 K): *δ* −6.85 (t, ^2^*J*_PSi_=3.10 Hz, N*Si*Me_3_), −1.17 (s, ThCH_2_*Si*Me_3_) p.p.m. FTIR *v*/cm^−1^ (Nujol): 1,591 (w), 1,403 (s), 1,302 (m), 861 (s), 695 (w), 608 (w), 588 (w), 551 (w).

### Preparation of [Th(BIPM^TMS^)_2_] (**3Th**)

Toluene (10 ml) was added to a mixture of [Th(BIPM^TMS^)(CH_2_SiMe_3_)_2_] (0.87 g, 0.90 mmol) and BIPM^TMS^H_2_ (0.51 g, 0.90 mmol). The resulting pale yellow solution was stirred at 50 °C for 20 h, and then was dried *in vacuo* to afford sticky yellow solid. The crude product was washed with hexanes (2 × 10 ml) and then dried *in vacuo* to afford **3Th** as a colourless powder. Yield: 0.63 g, 52%. Recrystallization of a small portion from toluene at 5 °C afforded colourless crystal suitable for single crystal X-ray diffraction. Anal. Calcd for C_62_H_76_N_4_P_4_Si_4_Th: C 55.34; H 5.69; N 4.16%. Found: C 55.02; H 5.78; N 3.91%. ^1^H NMR (C_6_D_6_, 298 K): *δ* 0.30 (s, 36 H, NSi(C*H*_3_)_3_), 7.03–7.12 (m, 25 H, Ar-*H*), 7.59–7.66 (m, 15 H, Ar-*H*). ^13^C{^1^H} NMR (C_6_D_6_, 298 K): *δ* 5.25 (s, NSi(*C*H_3_)_3_), 127.37 (s, Ar-*C*), 129.75 (s, Ar-*C*), 132.31 (s, Ar-*C*), 141.27 (t, ^3^*J*_PC_=47.0 Hz, *i-*Ar-*C*). The carbene centre in **3Th** was not observed in the ^13^C{^1^H} NMR spectrum. ^31^P{^1^H} NMR (C_6_D_6_, 298 K): *δ* 6.25 (ThC*P*_2_). ^29^Si{^1^H} NMR (C_6_D_6_, 298 K): *δ* −6.83 (N*Si*(CH_3_)_3_). FTIR *ν*/cm^−1^ (Nujol): 1,305 (m), 1,244 (w), 1,154 (m), 1,051 (s), 804 (s), 741 (w), 722 (s), 696 (w), 634 (s), 492 (m).

### Preparation of [Tb(BIPM^TMS^)(BIPM^TMS^H)] (**1Tb**)

BIPM^TMS^H_2_ (4.47 g, 8 mmol) in toluene (10 ml) was added dropwise to a precooled (−78 °C) suspension of [Tb(CH_2_Ph)_3_(THF)_3_] (2.32 g, 4 mmol) in toluene (15 ml). The resulting orange suspension was warmed to room temperature with stirring over 16 h and then refluxed for 10 min to afford a yellow solution. Volatiles were removed *in vacuo* and the resulting yellow residue recrystallized from hot toluene (4 ml) to afford colourless crystals of **1Tb** on cooling to room temperature. Yield: 1.81 g, 36%. Anal. Calcd for C_62_H_77_N_4_P_4_Si_4_Tb: C, 58.50; H, 6.10; N, 4.40%. Found: C, 58.61; H, 6.06; N, 4.33%. FTIR *v*/cm^−1^ (Nujol): 1,367 (s), 1,242 (m), 1,219 (m), 1,105 (s), 1,031 (m), 841 (s), 696 (m), 638 (w), 610 (w), 553 (m), 522 (m). Magnetic moment (Evans method, C_6_D_6_, 298 K): *μ*_eff_=9.90 *μ*_B_.

### Preparation of [Pr(BIPM^TMS^)_2_][K(18C6)(THF)_2_] (**2Pr**)

THF (15 ml) was added to a precooled (−78 °C) mixture of **1Pr** (5.25 g, 4.18 mmol) and [K(CH_2_Ph)] (0.55 g, 4.18 mmol). The resulting orange suspension was allowed to slowly warm to room temperature with stirring over 16 h to afford an orange solution. 18C6 (1.11 g, 4.18 mmol) in THF was added and stirred for a further 2 h. The solvent was removed *in vacuo* to afford an orange solid. The solid was washed with toluene to afford **2Pr** as a yellow powder. Yield: 4.50 g, 63%. Recrystallization of a small portion from toluene afforded yellow crystals of **2Pr** on storing at room temperature. Anal. Calcd for C_82_H_116_KN_4_O_8_P_4_PrSi_4_: C 57.89; H, 6.87; N, 3.29%. Found: C, 56.72; H, 6.67; N, 3.29%. FTIR *v*/cm^−1^ (Nujol): 1,352 (w), 1,302 (w), 1,105 (m), 964 (w), 826 (w), 695 (w), 634 (w), 522 (m). Magnetic moment (Evans method, THF, 298 K): *μ*_eff_=3.56 *μ*_B_.

### Preparation of [Tb(BIPM^TMS^)_2_][K(18C6)(THF)_2_] (**2Tb**)

THF (15 ml) was added to a precooled (−78 °C) mixture of **1Tb** (0.94 g, 0.74 mmol) and [K(CH_2_Ph)] (0.096 g, 0.74 mmol). The resulting orange suspension was allowed to slowly warm to room temperature with stirring over 16 h to afford a yellow solution. 18C6 (0.31 g, 1.17 mmol) in THF was then added and the resulting yellow solution stirred for 2 h. The solution was then reduced in volume to *ca*. 2 ml that afforded colourless crystals of **2Tb** on standing at room temperature. Yield: 0.26 g, 38%. Anal. Calcd for C_82_H_116_KN_4_O_8_P_4_Si_4_Tb: C, 57.27; H, 6.80; N, 3.26%. Found: C, 56.67; H, 6.63; N, 3.34%. FTIR *v*/cm^−1^ (Nujol): 1,351 (w), 1,303 (w), 1,071 (s), 960 (m), 848 (m), 760 (m), 743 (s), 700 (m), 634 (m), 523 (s). Magnetic moment (Evans method, THF, 298 K): μ_eff_=10.55 *μ*_B_.

### Preparation of [Pr(BIPM^TMS^)_2_Ag] (**6Pr**)

Toluene (15 ml) was added to a precooled (−78 °C) mixture of **2Pr** (1.70 g, 1.00 mmol) and [Ag(BPh_4_)] (0.43 g, 1.00 mmol). The resulting yellow suspension was allowed to slowly warm to room temperature with stirring over 16 h. The suspension was filtered and volatiles removed *in vacuo* and the resulting solid washed with hexanes (10 ml) to afford **6Pr** as a colourless powder. Yield 1.18 g, 87%. Recrystallization of a small portion from toluene afforded colourless crystals of **6Pr**. Anal. Calcd for C_62_H_76_AgN_4_P_4_PrSi_4_·1.25(C_7_H_8_): C 57.54; H, 5.87; N, 3.79%. Found: C, 57.44; H, 5.84; N, 3.69%. ^31^P{^1^H} NMR (C_6_D_6_, 298 K): δ −98.83 (1P, PrC*P*_2_), −83.25 (1P, PrC*P*_2_), 33.99 (2P, PrC*P*_2_) p.p.m. FTIR *v*/cm^−1^ (Nujol): 1,244 (w), 1,080 (s), 1,026 (s), 831 (m), 768 (m), 732 (m), 657 (m), 606 (w), 575 (w), 526 (w). Magnetic moment (Evans method, *d*_8_-THF, 298 K): *μ*_eff_=2.93 *μ*_B_.

### Preparation of [Tb(BIPM^TMS^)_2_Ag] (**6Tb**)

Toluene (15 ml) was added to a precooled (−78 °C) mixture of **2Tb** (1.72 g, 1.00 mmol) and [Ag(BPh_4_)] (0.43 g, 1.00 mmol). The resulting yellow suspension was allowed to slowly warm to room temperature with stirring over 16 h. The brown suspension was filtered and volatiles reduced in volume to 2 ml to afford colourless crystals of **6Tb** upon storage at room temperature. Yield 0.42 g, 31%. Anal. Calcd for C_62_H_76_AgN_4_P_4_Si_4_Tb·0.8(C_7_H_8_): C, 55.84; H, 5.71; N, 3.85%. Found: C, 55.85; H, 5.81; N, 3.83%. FTIR *v*/cm^−1^ (Nujol): 1,435 (m), 1,243 (w), 1,167 (m), 1,106 (s), 1,060 (s), 833 (s), 769 (m), 715 (m), 693 (m), 611 (w), 527 (w). Magnetic moment (Evans method, *d*_8_-THF, 298 K): *μ*_eff_=9.12 *μ*_B_.

### Reaction of [Ce(BIPM^TMS^)_2_] with PhCHO

Benzaldehyde (2.5 mg, 24 μmol) was added to a solution of [Ce(BIPM^TMS^)_2_] (15 mg, 12 μmol) in *d*_6_-benzene (0.4 ml). The reaction mixture was shaken vigorously, forming a green reaction mixture. The reaction mixture was stored at room temperature for 16 h and then analysed by multinuclear NMR that revealed a small amount of conversion to (Me_3_SiNPPh_2_)_2_C=C(H)Ph. The reaction mixture was then heated to 60 °C for 48 h. Analysis of the crude mixture showed quantitative conversion to (Me_3_SiNPPh_2_)_2_C=C(H)Ph. All spectroscopic data matched previously reported data^67^.

### Reaction of [Ce(BIPM^TMS^)_2_] with ArCHO

*d*_6_-benzene (0.4 ml) was added to a mixture of 9-anthracene carboxaldehyde (4.9 mg, 24 μmol) and [Ce(BIPM^TMS^)_2_] (15 mg, 12 μmol). The reaction mixture was shaken vigorously, forming a green reaction mixture. The reaction mixture was stored at room temperature for 16 h, heated to 60 °C for 48 h and heated to 80 °C for 48 h. Analysis of the crude mixture showed no conversion to (Me_3_SiNPPh_2_)_2_C=C(H)Ar.

### Reaction of [U(BIPM^TMS^)_2_] with PhCHO

Benzaldehyde (6.4 mg, 60 μmol) was added to a solution of [U(BIPM^TMS^)_2_] (40.5 mg, 30 μmol) in *d*_6_-benzene (0.4 ml). The reaction mixture was shaken vigorously, forming a brown reaction mixture. The reaction mixture was stored at room temperature for 48 h and then analysed by multinuclear NMR that revealed 95% conversion to (Me_3_SiNPPh_2_)_2_C=C(H)Ph. All spectroscopic data matched previously reported data^67^.

### Reaction of [U(BIPM^TMS^)_2_] with ArCHO

*d*_6_-benzene (0.4 ml) was added to a mixture of 9-anthracene carboxaldehyde (12.4 mg, 60 μmol) and [U(BIPM^TMS^)_2_] (40.5 mg, 30 μmol). The reaction mixture was shaken vigorously, forming a brown reaction mixture. The reaction mixture was stored at room temperature for 96 h and then analysed by multinuclear NMR that revealed 90% conversion to (Me_3_SiNPPh_2_)_2_C=C(H)Ar. All spectroscopic data matched previously reported data^40^.

### Reaction of [Th(BIPM^TMS^)_2_] with PhCHO

Benzaldehyde (6.4 mg, 60 μmol) was added to a solution of [Th(BIPM^TMS^)_2_] (40.1 mg, 30 μmol) in *d*_6_-benzene (0.4 ml). The reaction mixture was shaken vigorously, forming a colourless reaction mixture. The reaction mixture was stored at room temperature for 36 h and then analysed by multinuclear NMR that revealed 95% conversion to (Me_3_SiNPPh_2_)_2_C=C(H)Ph. All spectroscopic data matched previously reported data^67^.

### Reaction of [Th(BIPM^TMS^)_2_] with ArCHO

*d*_6_-benzene (0.4 ml) was added to a mixture of 9-anthracene carboxaldehyde (12.4 mg, 60 μmol) and [Th(BIPM^TMS^)_2_] (40.1 mg, 30 μmol). The reaction mixture was shaken vigorously, forming a yellow reaction mixture. The reaction mixture was stored at room temperature for 48 h and then analysed by multinuclear NMR that revealed 95% conversion to (Me_3_SiNPPh_2_)_2_C=C(H)Ar. All spectroscopic data matched previously reported data^40^.

### Data availability

The X-ray crystallographic coordinates (cif format) for structures reported in this article have been deposited at the Cambridge Crystallographic Data Centre (CCDC), under deposition numbers 1500929-1500939. These data can be obtained free of charge from The Cambridge Crystallographic Data Centre via www.ccdc.cam.ac.uk/data_request/cif. All other data are available from the corresponding authors on request.

## Additional information

**How to cite this article:** Gregson, M. *et al*. The inverse-*trans*-influence in tetravalent lanthanide and actinide *bis*(carbene) complexes. *Nat. Commun.* **8**, 14137 doi: 10.1038/ncomms14137 (2017).

**Publisher’s note:** Springer Nature remains neutral with regard to jurisdictional claims in published maps and institutional affiliations.

## Supplementary Material

Supplementary Information Supplementary Figures, Supplementary Tables, Supplementary Methods and Supplementary References

Supplementary Data 1 Crystallographic Information File for 1TB

Supplementary Data 2 Crystallographic Information File for 2Ce

Supplementary Data 3 Crystallographic Information File for 2Pr

Supplementary Data 4 Crystallographic Information File for 2Tb

Supplementary Data 5 Crystallographic Information File for 3Ce

Supplementary Data 6 Crystallographic Information File for 3U

Supplementary Data 7 Crystallographic Information File for 3Th

Supplementary Data 8 Crystallographic Information File for 5Th

Supplementary Data 9 Crystallographic Information File for 5U

Supplementary Data 10 Crystallographic Information File for 6Pr

Supplementary Data 11 Crystallographic Information File for 6Tb
