# Author Correction: The inverse-*trans*-influence in tetravalent lanthanide and actinide *bis*(carbene) complexes

**DOI:** 10.1038/ncomms16178

**Published:** 2018-02-05

**Authors:** Matthew Gregson, Erli Lu, David P. Mills, Floriana Tuna, Eric J. L. McInnes, Christoph Hennig, Andreas C. Scheinost, Jonathan McMaster, William Lewis, Alexander J. Blake, Andrew Kerridge, Stephen T. Liddle

Nature Communications 8: Article number: 14137; DOI: 10.1038/ncomms14137 (2017); Published: 02 03 2017; Updated: 02 05 2018

The original version of this Article contained a typographical error in Fig. 2, where reagent H_2_C(Ph_2_PNSiMe_3_)_2_ was incorrectly given as H_2_C(PNSiMe_3_)_2_. This has now been corrected in both the PDF and HTML versions of the Article.

