# Supplementary material for: The inverse-trans-influence in tetravalent lanthanide and actinide bis(carbene) complexes
Source: Nat Commun. 2017 Feb 3;8:14137. doi: 10.1038/ncomms14137 (PMC5296655; doi:10.1038/ncomms14137)
Supplement: Supplementary Information — Supplementary Figures, Supplementary Tables, Supplementary Methods and Supplementary References [file ncomms14137-s1.pdf]

Supplementary Figures

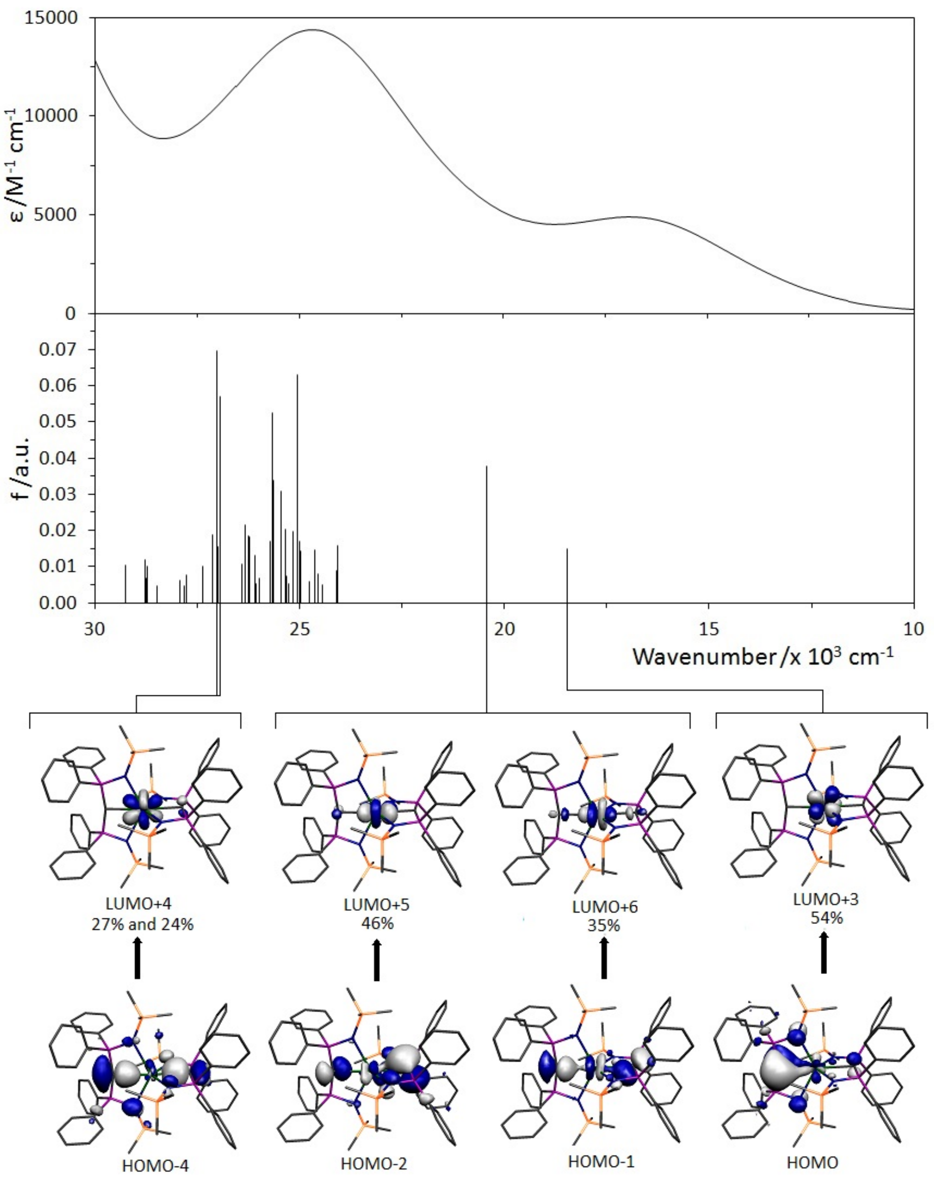

Supplementary Figure 1. Top: experimental optical spectrum of **3Ce** in toluene in the range 10,000-30,000 cm^{-1} (333-1,000 nm). Middle: Simulated UV/Vis/NIR electronic absorption spectrum of **3Ce** derived from TD-DFT with calculated oscillator strengths depicted as vertical black lines. Bottom: Selected electronic transitions corresponding to the individual bands of the calculated spectrum that principally correspond to electronic absorptions involving the σ- and π-components of the Ce=C double bond along with percentage contributions to these transitions. The remaining transitions involve the imino lone pairs. Hydrogen atoms are omitted for clarity.

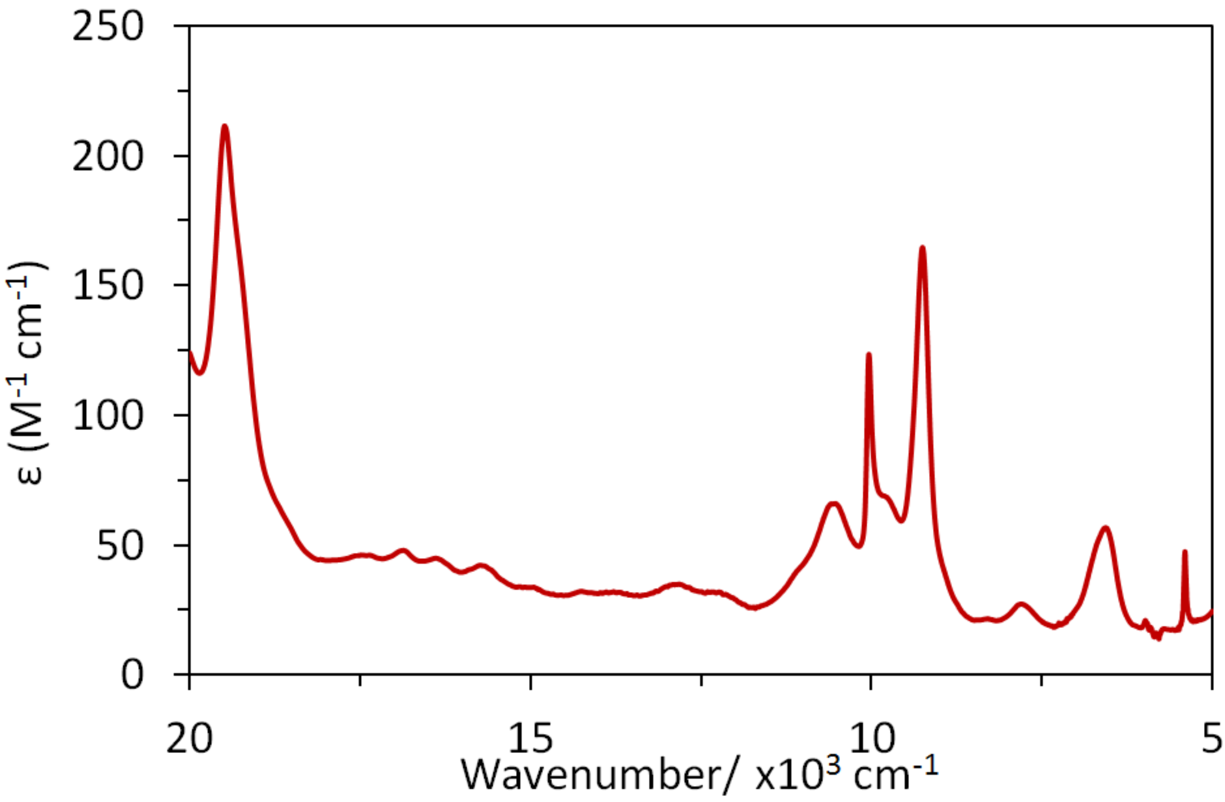

Supplementary Figure 2. Optical spectrum of **3U** in THF in the range 5,000-20,000 cm^{-1} (500-2,000 nm). The spectrum of colourless **3Th** is featureless in this range.

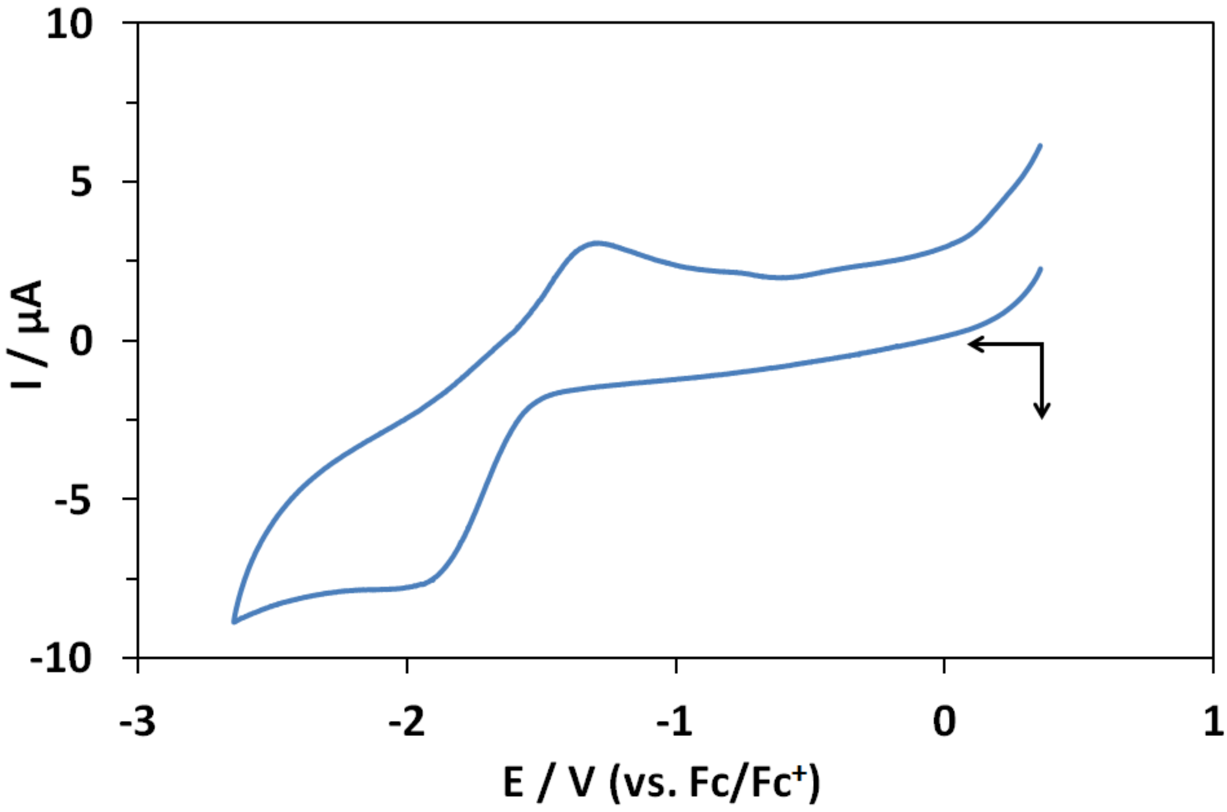

Supplementary Figure 3. Cyclic voltammogram of 2 mM **3Ce** in THF at 100 mV/s sweep rate (0.1 M $[N(Pr)_4][BAr^F{}_4]$ supporting electrolyte) vs. $[Fe(Cp)_2]^{0/+}$.

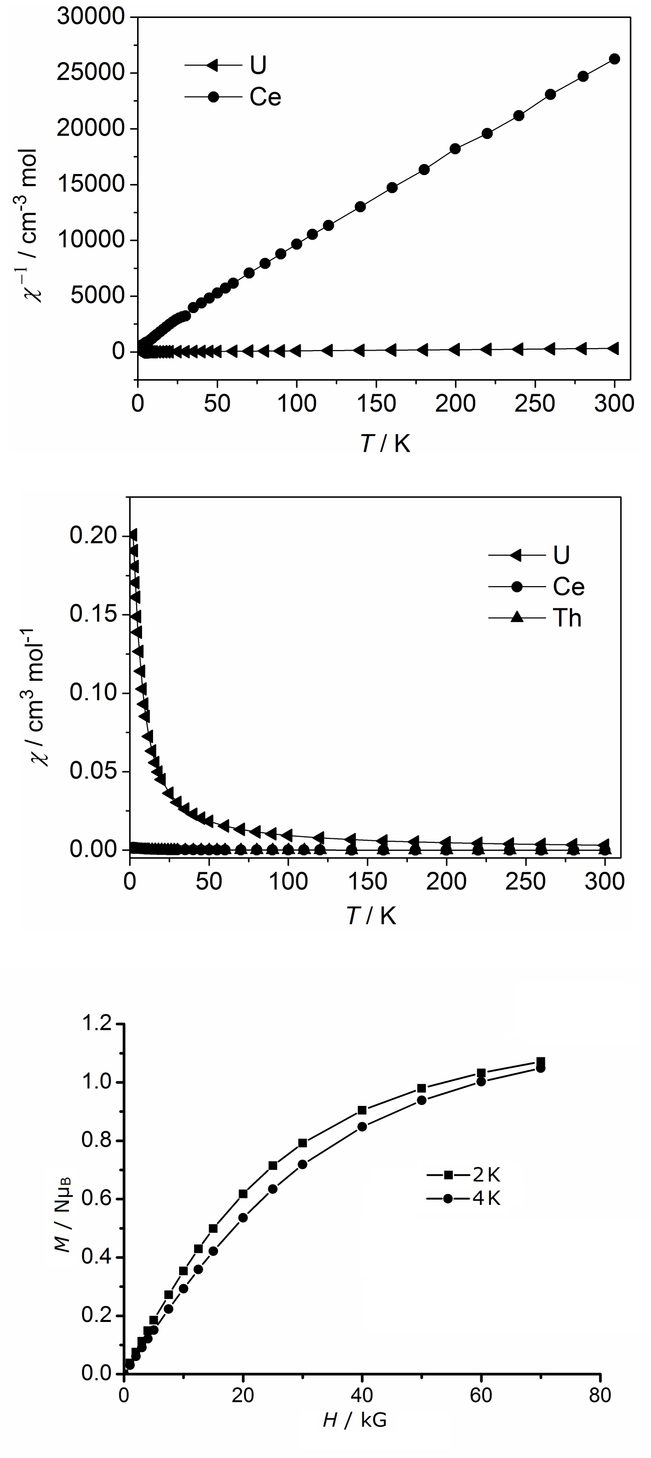

Supplementary Figure 4. Magnetic data for **3Ce**, **3U** and **3Th**. Top: χ^{-1} vs T. Middle: χ vs T. Bottom: molar magnetisation (M) vs applied magnetic field (H) data for **3U** at 2 and 4 K. Lines are a guide to the eye only.

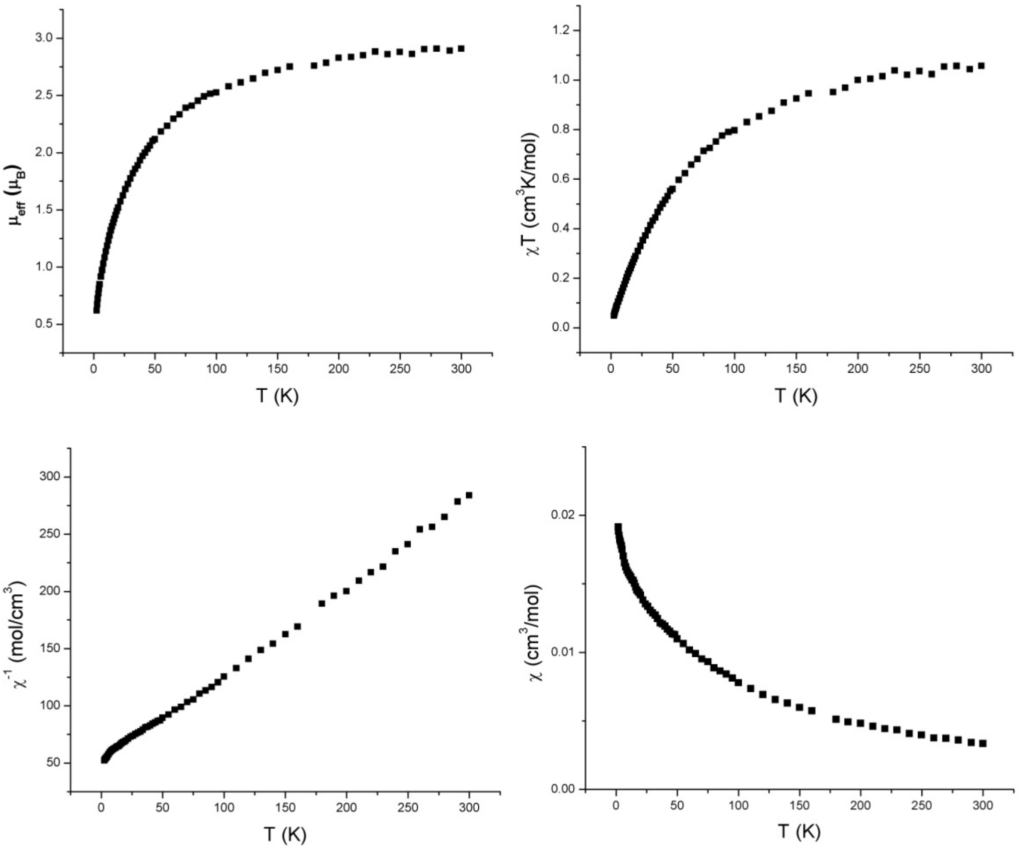

Supplementary Figure 5. Magnetic data for **5U**. Top left: μ_{eff} vs T. Top right: χT vs T. Bottom left: χ^{-1} vs T. Bottom right χ vs T.

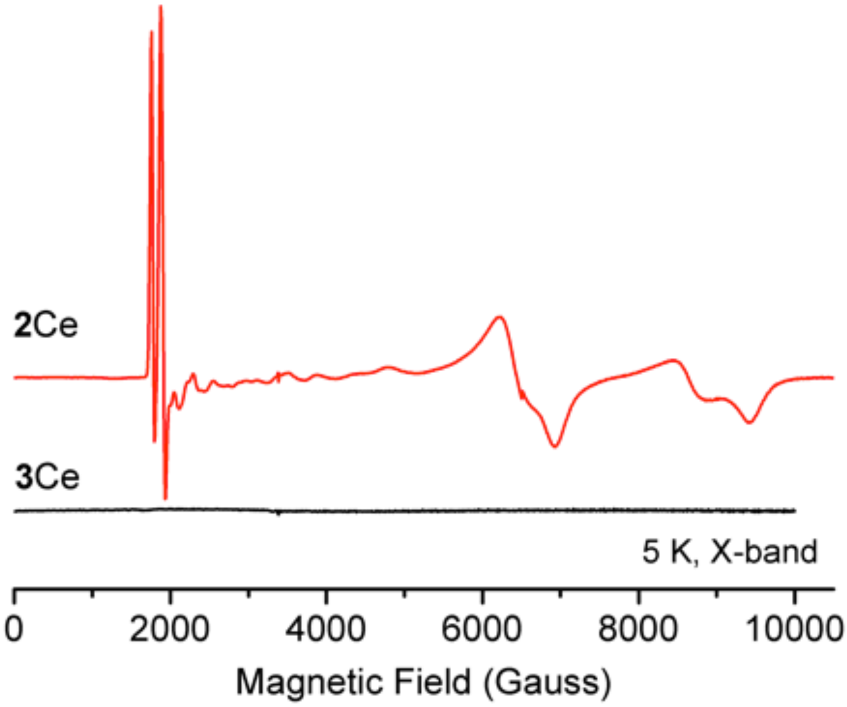

Supplementary Figure 6. X-band (9.4 GHz) EPR spectra of polycrystalline **2Ce** (red) and **3Ce** (black) at 5K.

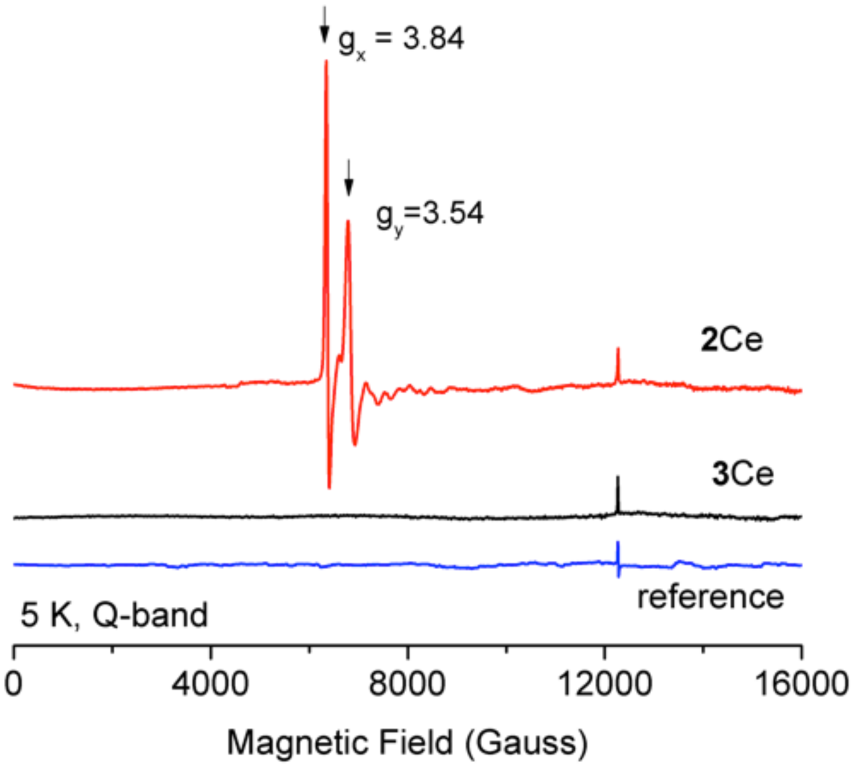

Supplementary Figure 7. Q-band (34 GHz) EPR spectra of polycrystalline **2Ce** (red), **3Ce** (black), and background (blue) at 5K. The very sharp linewidths, together with the very large g-anisotropy, lead to some spurious polycrystalline effects in the baseline at fields higher than g_z.

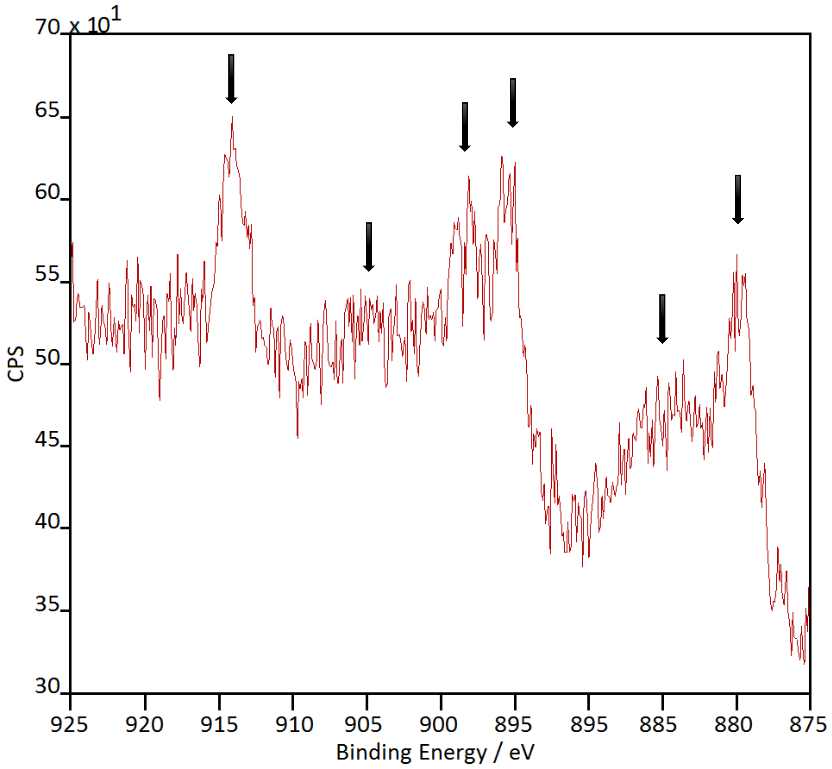

Supplementary Figure 8. XPS spectrum of **3Ce**. The six black arrows represent approximate positions of the six characteristic absorptions for CeO_2 and are to guide the eye only.

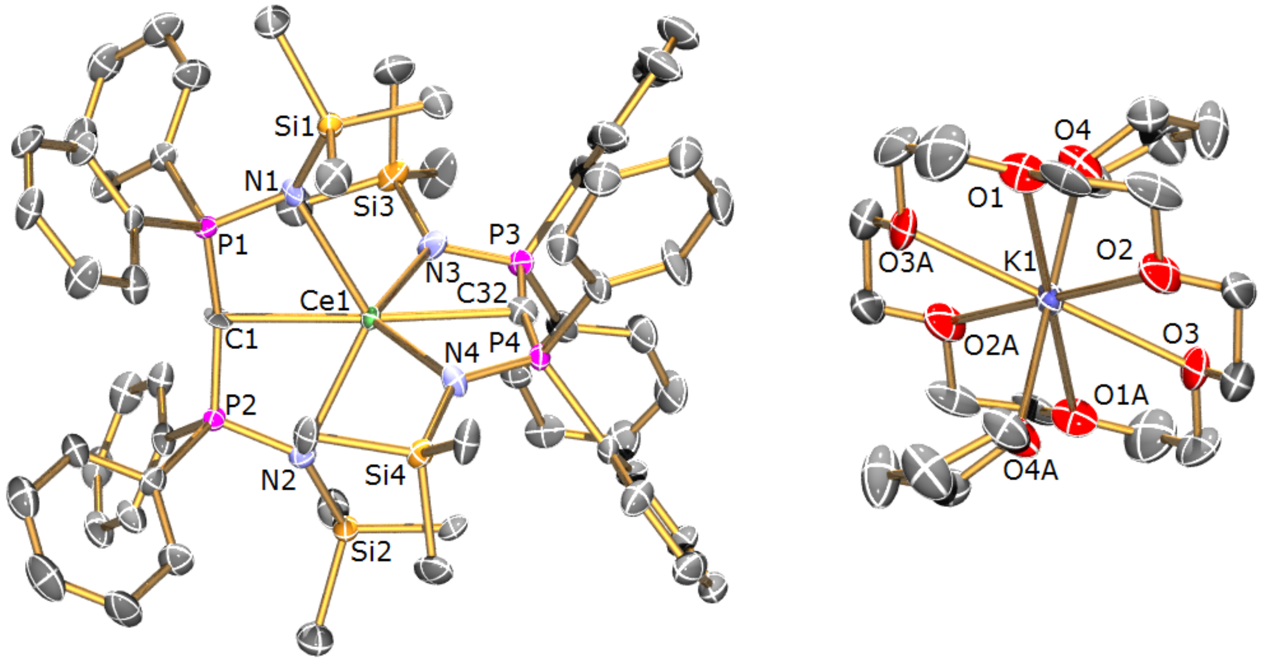

Supplementary Figure 9. Molecular structure of **2Ce**. Displacement ellipsoids set at 30% probability with hydrogen atoms and lattice solvent omitted for clarity.

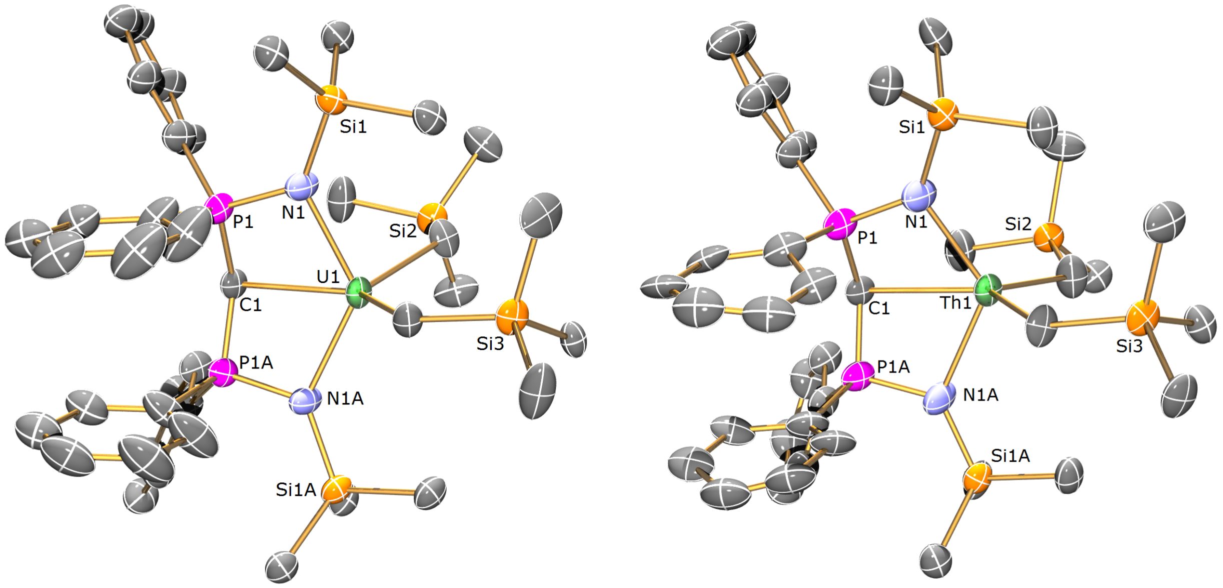

Supplementary Figure 10. Molecular structure of **5U** (left) and **5Th** (right). Displacement ellipsoids set at 30% probability with hydrogen atoms and lattice solvent omitted for clarity.

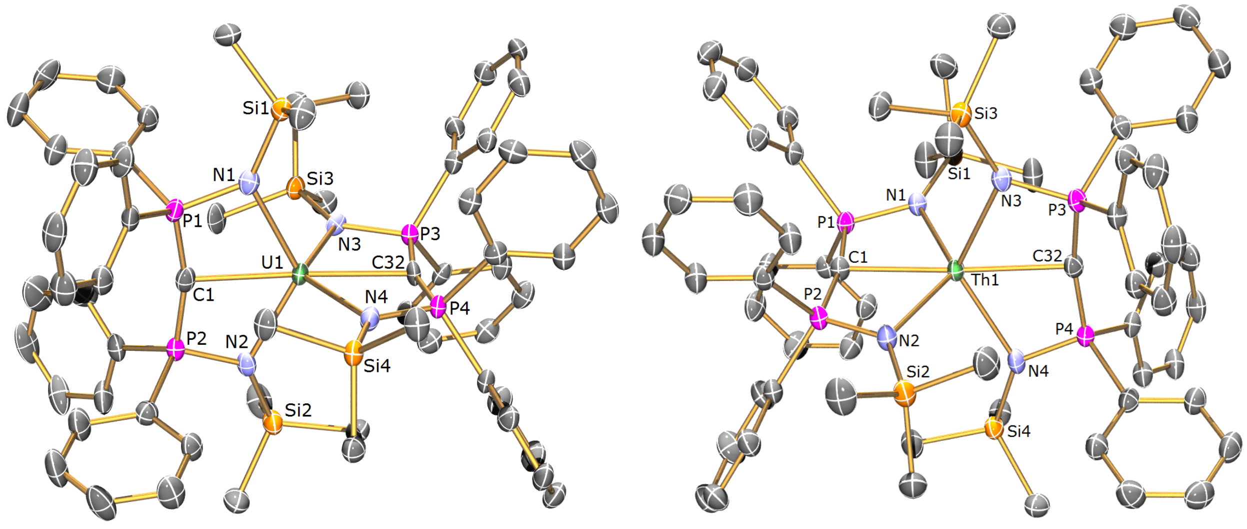

Supplementary Figure 11. Molecular structure of **3U** (left) and **3Th** (right). Displacement ellipsoids set at 30% probability with hydrogen atoms and lattice solvent omitted for clarity.

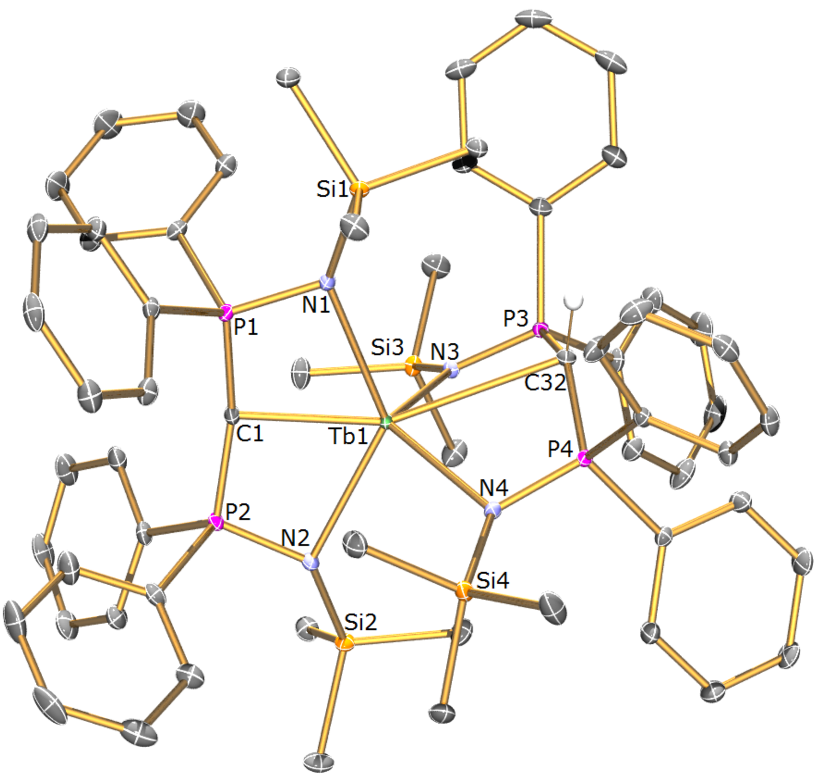

Supplementary Figure 12. Molecular structure of **1Tb**. Displacement ellipsoids set at 30% probability with hydrogen atoms and lattice solvent omitted for clarity.

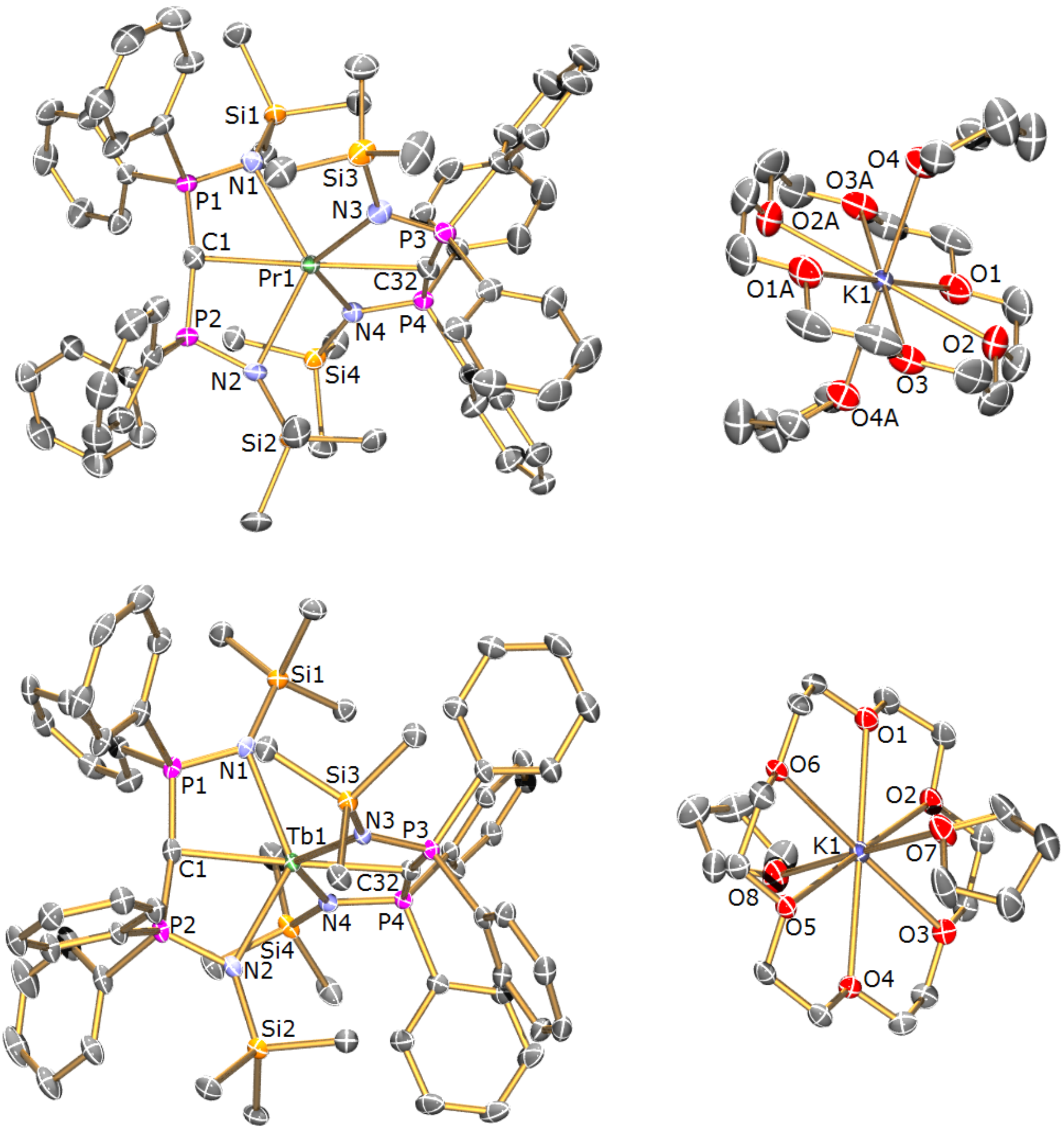

Supplementary Figure 13. Molecular structure of **2Pr** (top) and **2Tb** (bottom). Displacement ellipsoids set at 30% probability with hydrogen atoms and lattice solvent omitted for clarity.

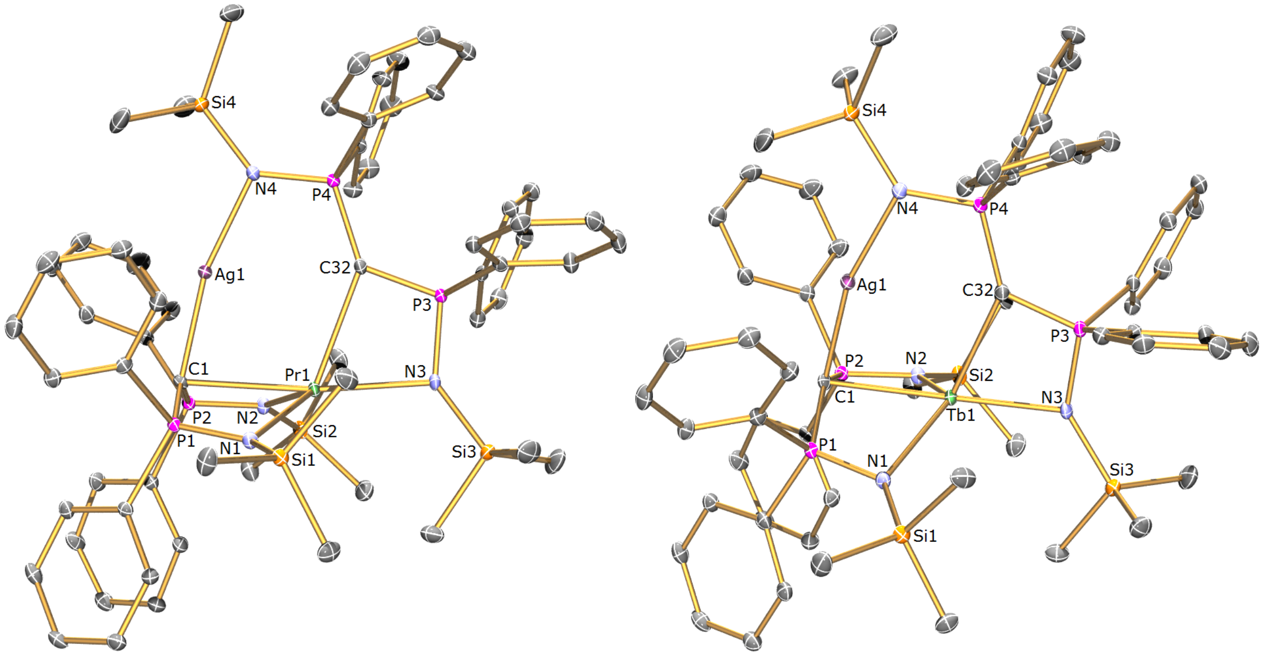

Supplementary Figure 14. Molecular structure of **6Pr** (left) and **6Tb** (right). Displacement ellipsoids set at 30% probability with hydrogen atoms and lattice solvent omitted for clarity.

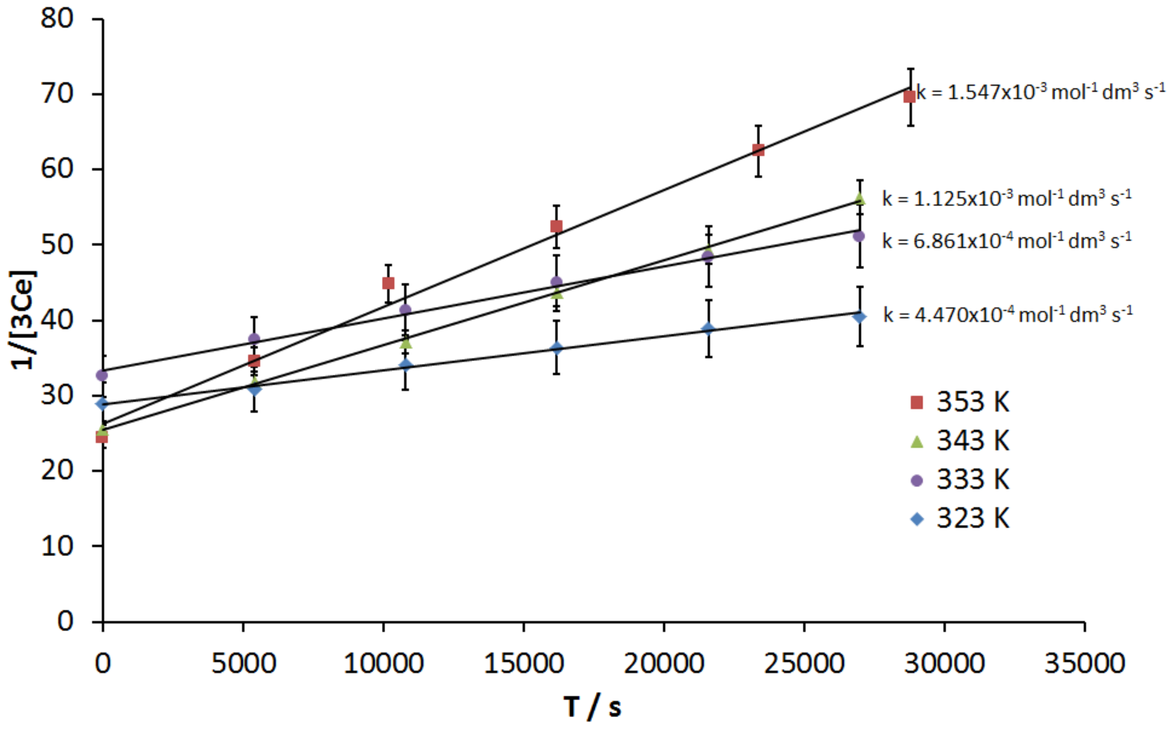

Supplementary Figure 15. Combined rate plots for the reactions of **3Ce** with PhCHO.

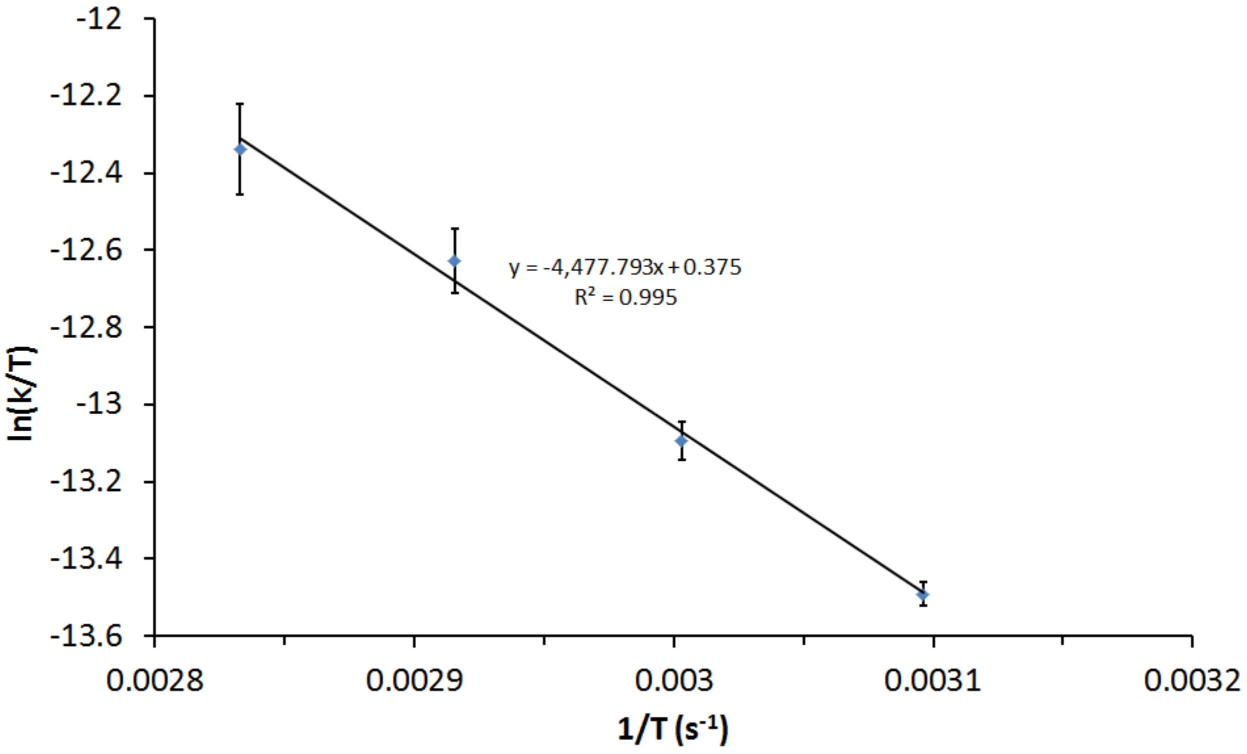

Supplementary Figure 16. Eyring plot for the reactions of **3Ce** with PhCHO.

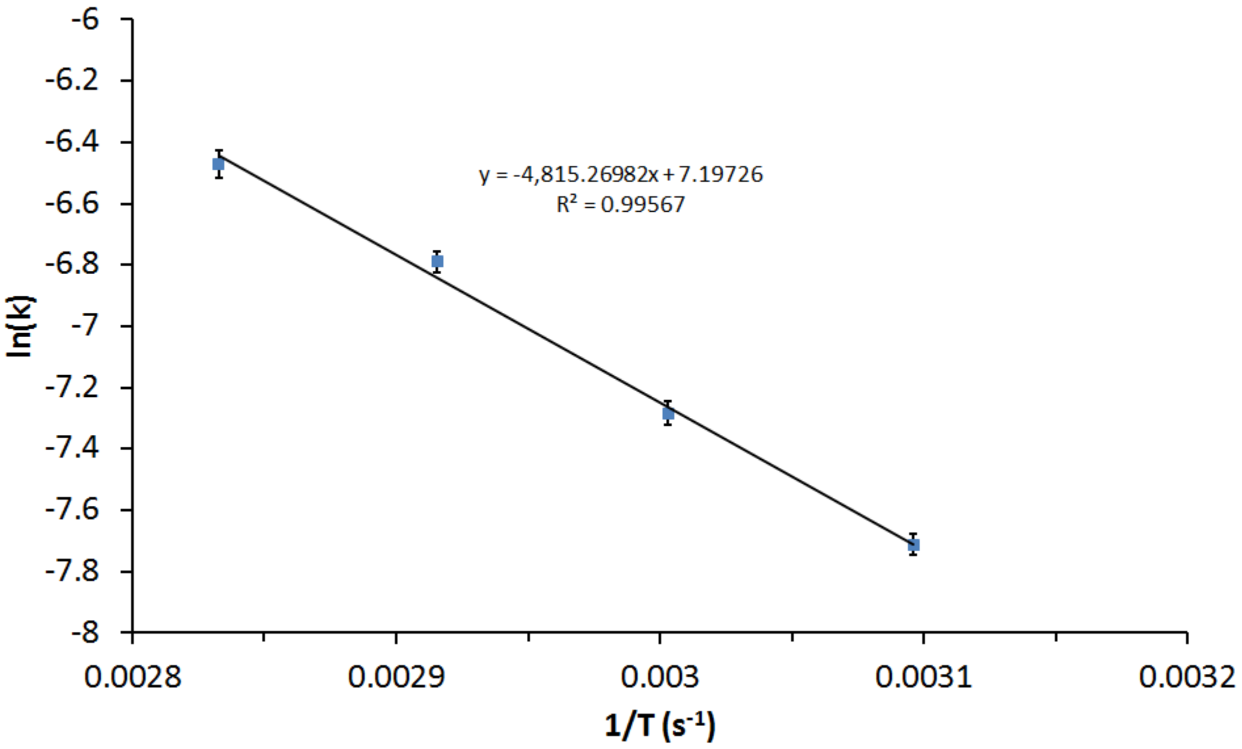

Supplementary Figure 17. Arrhenius plot for the reactions of **3Ce** with PhCHO.

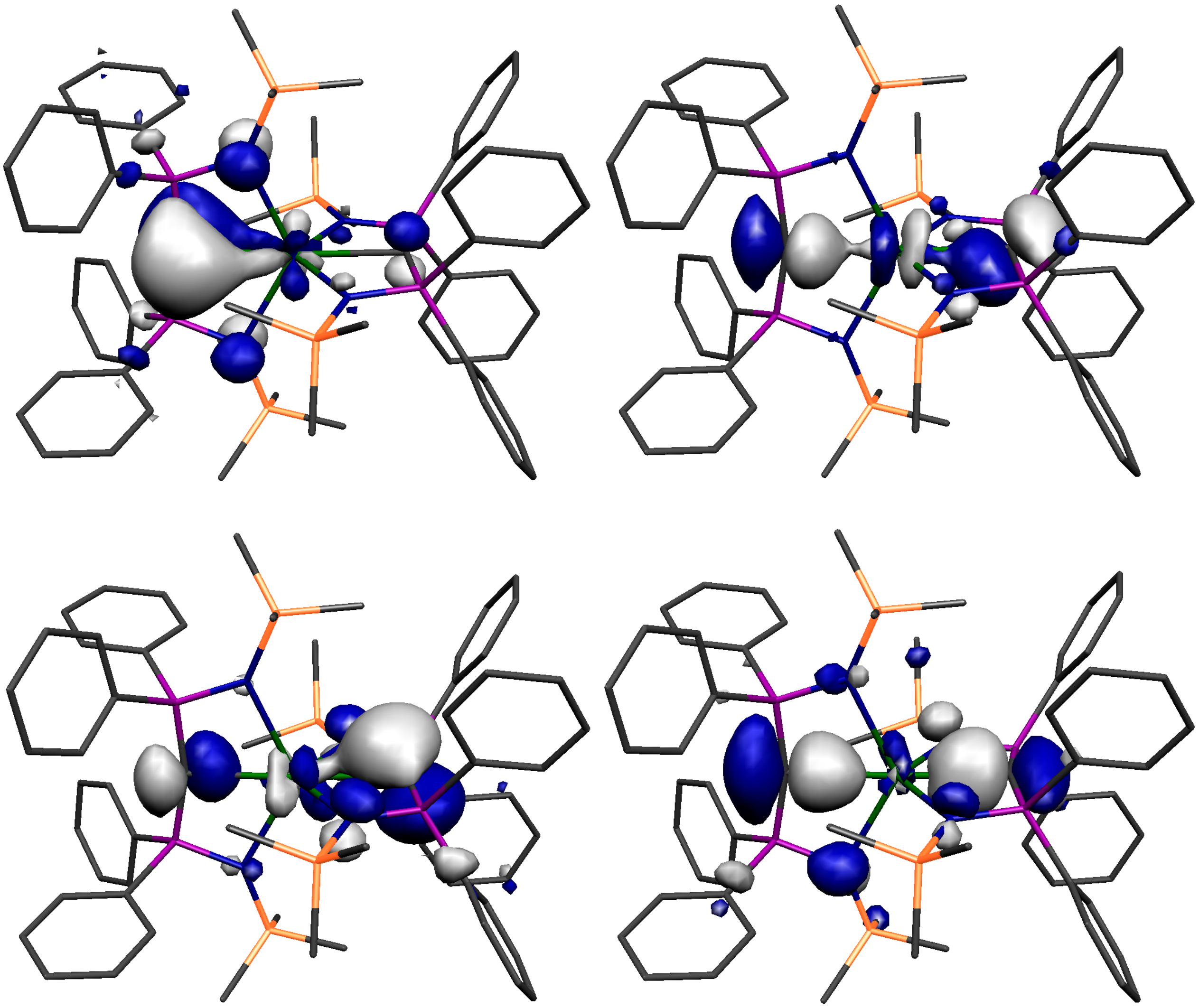

Supplementary Figure 18. Kohn Sham frontier molecular orbital representations for **3Ce**. Top left to bottom right: HOMO (325, −4.553 eV), HOMO−1 (324, −4.565 eV), HOMO−2 (323, −4.592 eV), and HOMO−4 (321, −5.476 eV).

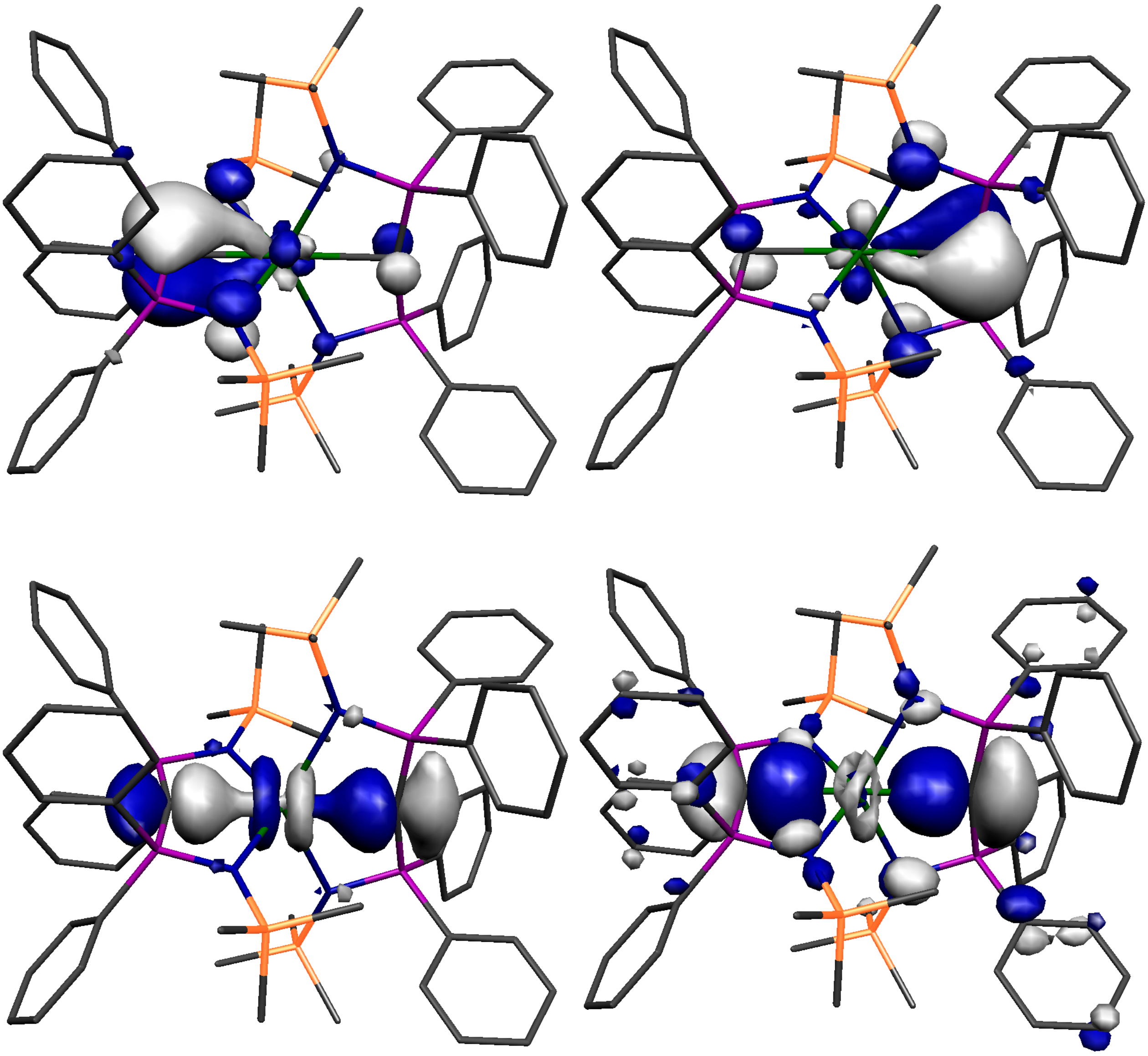

Supplementary Figure 19. Kohn Sham frontier molecular orbital representations for **3U**. HOMO (343a, −2.561 eV) and HOMO−1 (342a, −2.648 eV) are essentially pure, non-bonding 5f orbitals of $m_l = 2$ parentage and are consequently omitted. Top left to bottom right: HOMO−2 (341a, −4.633 eV), HOMO−3 (340a, −4.668 eV), HOMO−4 (339a, −4.718 eV), and HOMO−8 (335a, −5.662 eV).

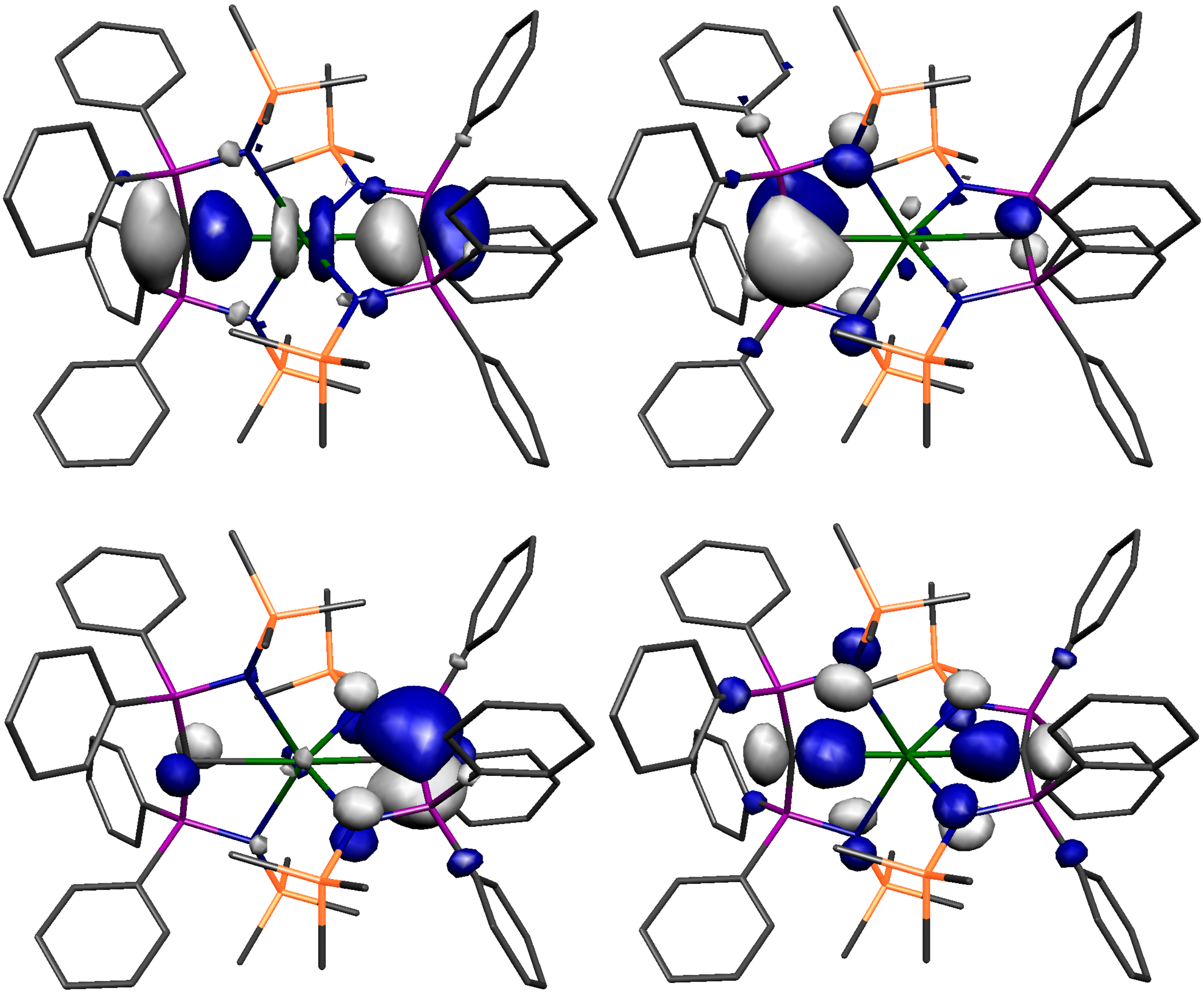

Supplementary Figure 20. Kohn Sham frontier molecular orbital representations for **3Th**. The σ*g* bonding combination is delocalised over more than one molecular orbital due to mixing with *N*-lone pair orbital coefficients of very similar energies, but the principal contribution is depicted in HOMO−3. Top left to bottom right: HOMO (341, −4.467 eV), HOMO−1 (340, −4.527 eV), HOMO−2 (339, −4.556 eV), and HOMO−3 (338, −5.396 eV).

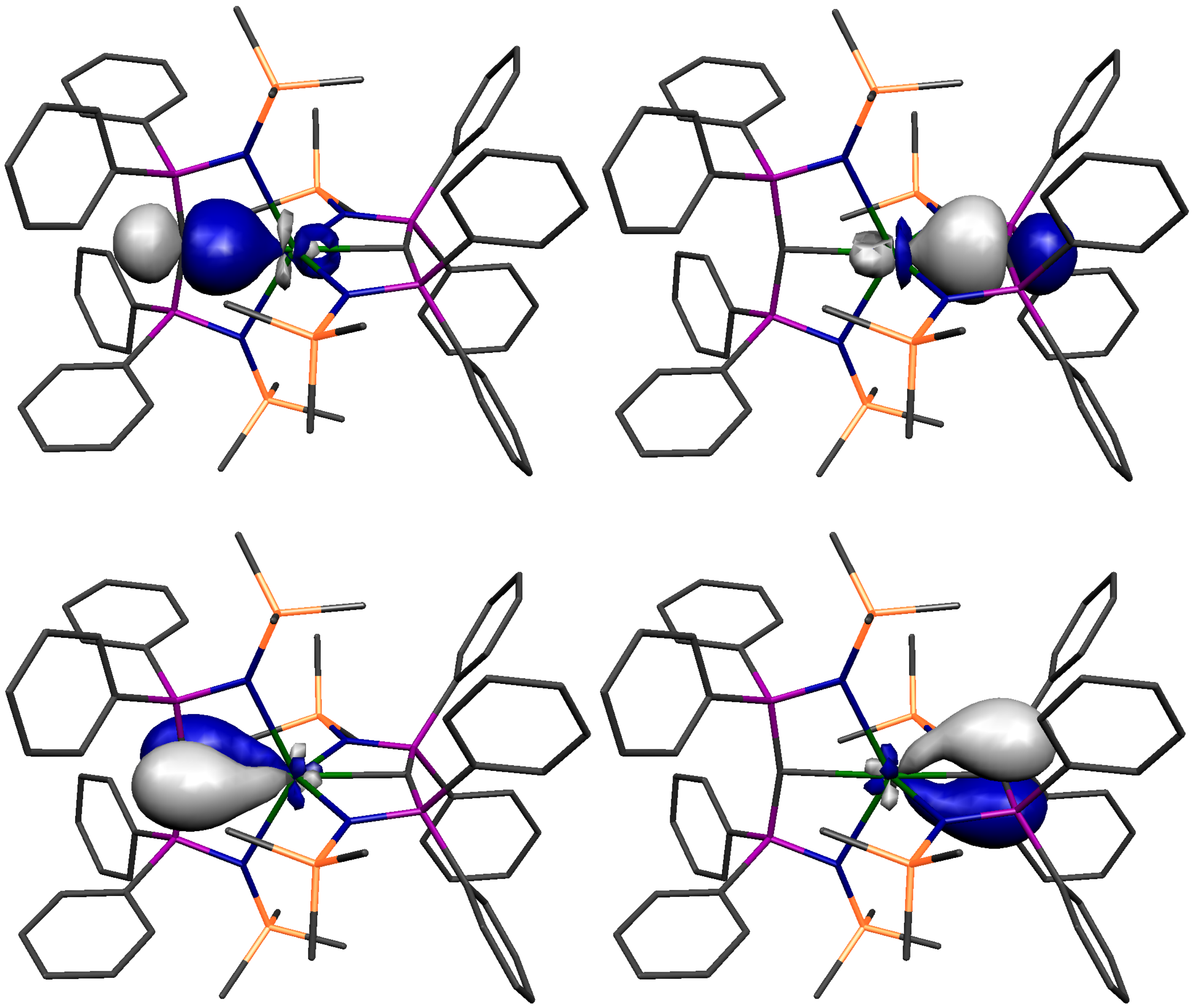

Supplementary Figure 21. NBO representations for **3Ce**. Top left to bottom right: σ- then π-bond interactions.

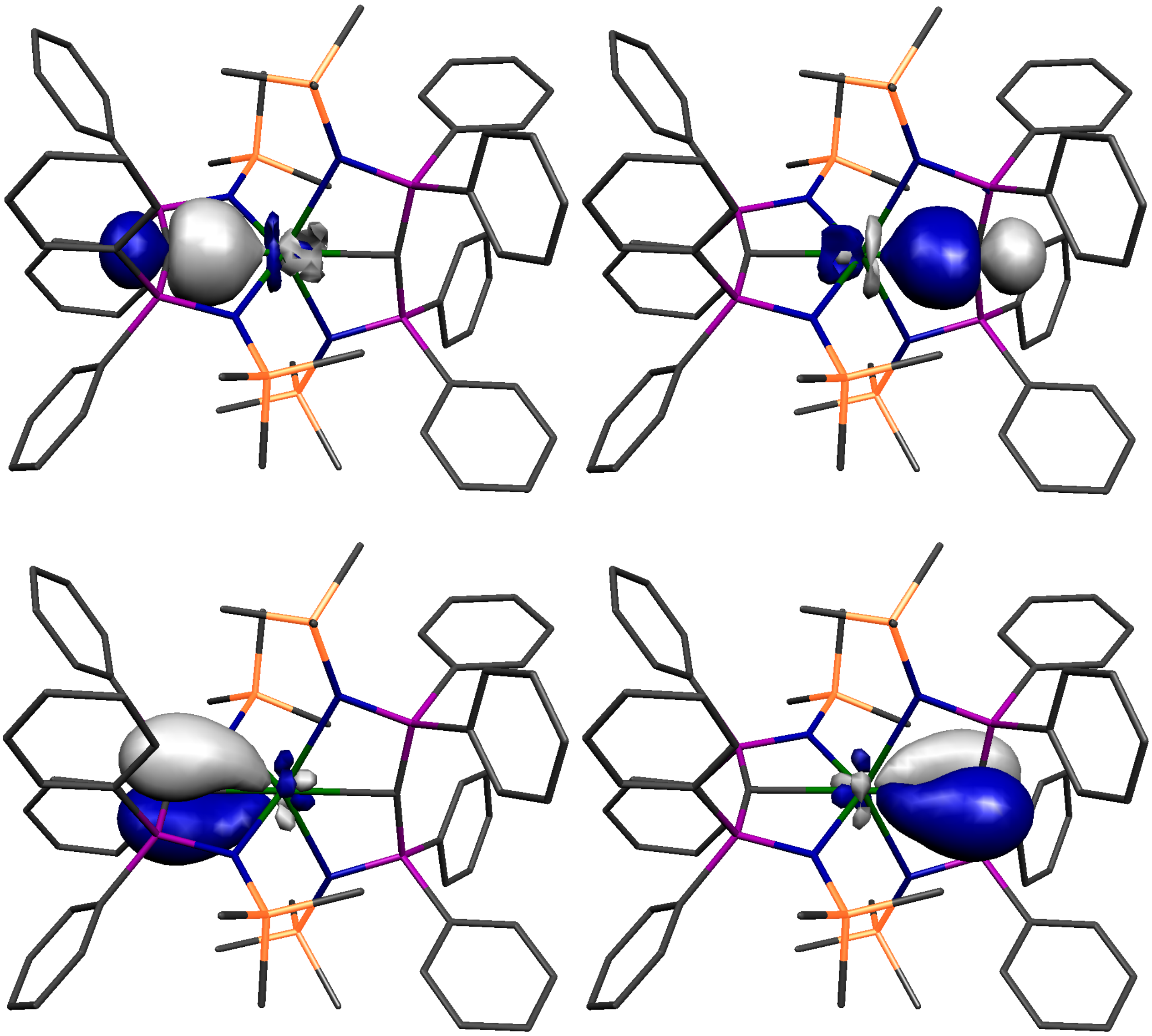

Supplementary Figure 22. NBO representations for **3U**. Top left to bottom right: σ- then π-bond interactions.

Supplementary Tables

Supplementary Table 1. Selected Crystallographic data (CCDC 1500929-1500939)

	**2Ce**	**3Ce**
Chemical formula	$C_{62}H_{76}CeN_4P_4Si_4\cdot 2(C_{10}H_{20}K_{0.5}O_4)\cdot 1(C_7H_8)$	$C_{62}H_{76}CeN_4P_4Si_4\cdot C_5H_{12}$
M_r	1793.37	1325.77
Crystal system, space group	Triclinic, *P-1*	Triclinic, *P-1*
Temperature (K)	120	120
a, *b*, *c* (Å)	16.9671 (6), 17.9463 (5), 19.1550 (6)	12.5621 (2), 13.9370 (3), 21.4408 (4)
α, β, γ (°)	71.281 (3), 64.086 (3), 65.421 (3)	95.8269 (16), 101.8739 (14), 109.0531 (17)
V (Å^3)	4701.2 (3)	3414.00 (12)
Z	2	2
Radiation type	Cu $K\alpha$	Cu $K\alpha$
μ (mm^{-1})	5.69	7.02
Crystal size (mm)	0.20 × 0.15 × 0.11	0.25 × 0.19 × 0.12
Diffractometer	SuperNova-Duo, Atlas diffractometer	SuperNovaII, Atlas diffractometer
Absorption correction	Gaussian *CrysAlis PRO*, Agilent Technologies, Version 1.171.37.34 (release 22-05-2014 CrysAlis171 .NET) (compiled May 22 2014,16:03:01) Numerical absorption correction based on gaussian integration over a multifaceted crystal model Empirical absorption correction using spherical harmonics, implemented in SCALE3 ABSPACK scaling algorithm.	Gaussian *CrysAlis PRO*, Agilent Technologies, Version 1.171.37.31 (release 14-01-2014 CrysAlis171 .NET) (compiled Jan 14 2014,18:38:05) Numerical absorption correction based on gaussian integration over a multifaceted crystal model Empirical absorption correction using spherical harmonics, implemented in SCALE3 ABSPACK scaling algorithm.
T_{min}, T_{max}	0.433, 0.659	0.345, 0.550
No. of measured, independent and observed [$I > 2\sigma(I)$] reflections	37567, 18660, 17879	25253, 13558, 13060
R_{int}	0.041	0.031
$(\sin \theta/\lambda)_{max}$ (Å^{-1})	0.625	0.625
$R[F^2 > 2\sigma(F^2)]$, $wR(F^2)$, S	0.042, 0.111, 1.02	0.029, 0.076, 1.02
No. of reflections	18660	13558
No. of parameters	1077	753
No. of restraints	177	42
H-atom treatment	H-atom parameters constrained	H-atom parameters constrained
$\Delta\rho_{max}$, $\Delta\rho_{min}$ (e Å^{-3})	0.93, −0.87	0.81, −0.58

	**3U**	**3Th**
Chemical formula	$C_{62}H_{76}N_4P_4Si_4U·C_6H_{14}$	$C_{62}H_{76}N_4P_4Si_4Th·0.5(C_7H_8)$
M_r	1437.70	1391.61
Crystal system, space group	Triclinic, *P-1*	Triclinic, *P-1*
Temperature (K)	90	120
a, *b*, *c* (Å)	12.6142 (6), 13.8834 (5), 21.4347 (8)	12.9528 (3), 14.0462 (5), 19.2134 (3)
α, β, γ (°)	95.968 (3), 101.233 (3), 109.178 (4)	95.067 (2), 92.9824 (18), 110.180 (3)
V (Å^3)	3420.0 (2)	3255.49 (16)
Z	2	2
Radiation type	Cu *K*α	Cu *K*α
μ (mm $^{-1}$)	8.55	9.34
Crystal size (mm)	0.36 × 0.23 × 0.08	0.21 × 0.13 × 0.09
Diffractometer	SuperNova, Single source at offset, Atlas diffractometer	SuperNovaII, Atlas diffractometer
Absorption correction	Gaussian *CrysAlis PRO*, Agilent Technologies, Version 1.171.37.35 (release 13-08-2014 CrysAlis171 .NET) (compiled Aug 13 2014,18:06:01) Numerical absorption correction based on gaussian integration over a multifaceted crystal model Empirical absorption correction using spherical harmonics, implemented in SCALE3 ABSPACK scaling algorithm.	Gaussian *CrysAlis PRO*, Agilent Technologies, Version 1.171.37.33 (release 27-03-2014 CrysAlis171 .NET) (compiled Mar 27 2014,17:12:48) Numerical absorption correction based on gaussian integration over a multifaceted crystal model Empirical absorption correction using spherical harmonics, implemented in SCALE3 ABSPACK scaling algorithm.
T_{min}, T_{max}	0.377, 0.758	0.135, 0.355
No. of measured, independent and observed [$I > 2\sigma(I)$] reflections	27751, 13610, 11971	23373, 12741, 12556
R_{int}	0.057	0.023
$(\sin \theta/\lambda)_{max}$ (Å^{-1})	0.628	0.622
$R[F^2 > 2\sigma(F^2)]$, $wR(F^2)$, S	0.053, 0.141, 1.04	0.023, 0.061, 1.05
No. of reflections	13610	12741
No. of parameters	688	751
No. of restraints	0	103
H-atom treatment	H-atom parameters constrained	H-atom parameters constrained
$\Delta\rho_{max}$, $\Delta\rho_{min}$ (e Å^{-3})	3.22, −2.04	1.71, −1.37
	**5U**	**5Th**
Chemical formula	$C_{39}H_{60}N_2P_2Si_4U$	$C_{39}H_{60}N_2P_2Si_4Th$
M_r	969.22	963.23
Crystal system, space	Orthorhombic, *Pnma*	Orthorhombic, *Pnma*

group		
Temperature (K)	90	90
a, *b*, *c* (Å)	19.1815 (18), 19.1250 (19), 12.1896 (12)	19.2611 (16), 19.2083 (18), 12.2300 (11)
α, β, γ (°)		
V (Å^3)	4471.7 (7)	4524.8 (7)
Z	4	4
Radiation type	Cu *K*α	Mo *K*α
μ (mm^{-1})	12.12	3.50
Crystal size (mm)	0.21 × 0.10 × 0.07	1.49 × 0.32 × 0.26
Diffractometer	SuperNova, Single source at offset), Atlas diffractometer	Bruker *SMART APEX* CCD area detector diffractometer
Absorption correction	Gaussian *CrysAlis PRO*, Agilent Technologies, Version 1.171.35.19 (release 27-10-2011 CrysAlis171 .NET) (compiled Oct 27 2011,15:02:11) Numerical absorption correction based on gaussian integration over a multifaceted crystal model	Multi-scan *SADABS2007*/2 (Bruker,2007) was used for absorption correction. wR2(int) was 0.1370 before and 0.0775 after correction. The Ratio of minimum to maximum transmission is 0.3202. The λ/2 correction factor is 0.0015.
T_{min}, T_{max}	0.264, 0.619	0.022, 0.068
No. of measured, independent and observed [$I > 2\sigma(I)$] reflections	31480, 4684, 3975	53724, 5417, 3996
R_{int}	0.103	0.085
$(\sin \theta/\lambda)_{max}$ (Å^{-1})	0.630	0.653
$R[F^2 > 2\sigma(F^2)]$, $wR(F^2)$, S	0.086, 0.236, 1.04	0.083, 0.239, 1.17
No. of reflections	4684	5417
No. of parameters	314	368
No. of restraints	348	204
H-atom treatment	H-atom parameters constrained	H-atom parameters constrained
$\Delta\rho_{max}$, $\Delta\rho_{min}$ (e Å^{-3})	12.25, −3.24	7.91, −4.11
	**1Tb**	**2Pr**
Chemical formula	$C_{62}H_{77}N_4P_4Si_4Tb$	$C_{62}H_{76}N_4P_4PrSi_4 \cdot 2(C_{10}H_{20}K_{0.5}O_4) \cdot C_7H_8$
M_r	1273.43	1794.16
Crystal system, space group	Monoclinic, *C*2/*c*	Triclinic, *P-1*
Temperature (K)	120	120
a, *b*, *c* (Å)	31.7332 (11), 25.7464 (4), 20.9820 (8)	16.9776 (6), 17.8949 (6), 19.1874 (7)
α, β, γ (°)	132.711 (6)	71.135 (3), 64.209 (4), 65.333 (3)
V (Å^3)	12596.2 (12)	4697.6 (3)
Z	8	2

Radiation type	Cu *K*α	Cu *K*α
μ (mm $^{-1}$)	7.52	5.93
Crystal size (mm)	0.26 × 0.16 × 0.15	0.28 × 0.19 × 0.11
Diffractometer	GV1000, Atlas diffractometer	GV1000, Atlas diffractometer
Absorption correction	Gaussian *CrysAlis PRO*, Agilent Technologies, Version 1.171.36.28a (release 18-03-2013 CrysAlis171 .NET) (compiled Mar 18 2013,11:47:30) Numerical absorption correction based on gaussian integration over a multifaceted crystal model	Gaussian *CrysAlis PRO*, Agilent Technologies, Version 1.171.37.33 (release 27-03-2014 CrysAlis171 .NET) (compiled Mar 27 2014,17:12:48) Numerical absorption correction based on gaussian integration over a multifaceted crystal model Empirical absorption correction using spherical harmonics, implemented in SCALE3 ABSPACK scaling algorithm.
T_{min}, T_{max}	0.860, 1.102	0.393, 0.610
No. of measured, independent and observed [$I > 2\sigma(I)$] reflections	26621, 12518, 11733	35419, 18557, 17525
R_{int}	0.023	0.052
$(\sin \theta/\lambda)_{max}$ (Å^{-1})	0.625	0.625
$R[F^2 > 2\sigma(F^2)]$, $wR(F^2)$, S	0.024, 0.062, 1.02	0.056, 0.155, 1.05
No. of reflections	12518	18557
No. of parameters	688	1077
No. of restraints	0	177
H-atom treatment	H atoms treated by a mixture of independent and constrained refinement	H-atom parameters constrained
$\Delta\rho_{max}$, $\Delta\rho_{min}$ (e Å^{-3})	0.49, −0.61	3.24, −1.40
	**2Tb**	**6Pr**
Chemical formula	$C_{62}H_{76}N_4P_4Si_4Tb \cdot C_{20}H_{40}KO_8 \cdot 2(C_4H_8O)$	$C_{62}H_{76}AgN_4P_4PrSi_4 \cdot C_7H_8$
M_r	1864.25	1454.42
Crystal system, space group	Triclinic, *P-1*	Monoclinic, *C*2/*c*
Temperature (K)	120	120
a, *b*, *c* (Å)	15.1657 (5), 15.2813 (4), 21.1867 (5)	45.8315 (14), 13.00211 (19), 24.0634 (5)
α, β, γ (°)	98.753 (2), 94.959 (2), 93.489 (2)	104.354 (3)
V (Å^3)	4821.0 (2)	13891.9 (6)
Z	2	8
Radiation type	Cu *K*α	Cu *K*α
μ (mm $^{-1}$)	5.53	9.43

Crystal size (mm)	0.17 × 0.11 × 0.10	0.09 × 0.07 × 0.03
Diffractometer	SuperNova-Duo, Atlas diffractometer	GV1000, Atlas diffractometer
Absorption correction	Gaussian *CrysAlis PRO*, Agilent Technologies, Version 1.171.37.33 (release 27-03-2014 CrysAlis171 .NET) (compiled Mar 27 2014,17:12:48) Numerical absorption correction based on gaussian integration over a multifaceted crystal model Empirical absorption correction using spherical harmonics, implemented in SCALE3 ABSPACK scaling algorithm.	Gaussian *CrysAlis PRO*, Agilent Technologies, Version 1.171.37.31 (release 14-01-2014 CrysAlis171 .NET) (compiled Jan 14 2014,18:38:05) Numerical absorption correction based on gaussian integration over a multifaceted crystal model Empirical absorption correction using spherical harmonics, implemented in SCALE3 ABSPACK scaling algorithm.
T_{min}, T_{max}	0.718, 0.809	0.922, 0.968
No. of measured, independent and observed [$I > 2\sigma(I)$] reflections	37366, 19123, 16494	26628, 13746, 11696
R_{int}	0.055	0.033
$(\sin \theta/\lambda)_{max}$ (Å^{-1})	0.625	0.626
$R[F^2 > 2\sigma(F^2)]$, $wR(F^2)$, S	0.059, 0.164, 1.02	0.034, 0.083, 1.02
No. of reflections	19123	13746
No. of parameters	1053	761
No. of restraints	123	0
H-atom treatment	H-atom parameters constrained	H-atom parameters constrained
$\Delta\rho_{max}$, $\Delta\rho_{min}$ (e Å^{-3})	1.70, −1.25	1.00, −0.83

	**6Tb**
Chemical formula	$C_{62}H_{76}AgN_4P_4Si_4Tb \cdot C_7H_8$
M_r	1472.43
Crystal system, space group	Monoclinic, *C*2/*c*
Temperature (K)	120
a, *b*, *c* (Å)	45.786 (3), 12.9185 (7), 24.1821 (12)
α, β, γ (°)	104.683 (5)
V (Å^3)	13836.2 (13)
Z	8
Radiation type	Cu *K*α
μ (mm^{-1})	9.09
Crystal size (mm)	0.34 × 0.24 × 0.22
Diffractometer	GV1000, Atlas diffractometer
Absorption correction	Gaussian *CrysAlis PRO*, Agilent

	Technologies, Version 1.171.37.33 (release 27-03-2014 CrysAlis171 .NET) (compiled Mar 27 2014,17:12:48) Numerical absorption correction based on gaussian integration over a multifaceted crystal model Empirical absorption correction using spherical harmonics, implemented in SCALE3 ABSPACK scaling algorithm.
T_{min}, T_{max}	0.163, 0.314
No. of measured, independent and observed [$I > 2\sigma(I)$] reflections	27389, 13618, 12320
R_{int}	0.038
$(\sin \theta/\lambda)_{max}$ (Å^{-1})	0.624
$R[F^2 > 2\sigma(F^2)]$, $wR(F^2)$, S	0.034, 0.087, 1.02
No. of reflections	13618
No. of parameters	761
No. of restraints	0
H-atom treatment	H-atom parameters constrained
$\Delta\rho_{max}$, $\Delta\rho_{min}$ (e Å^{-3})	0.71, −0.88

Supplementary Table 2. Selected Bond Lengths (Å) and Angles (°)

	**2Ce**	**3Ce**	**3U**	**3Th**	**5U**	**5Th**
C1-P1	1.634(3)	1.664(2)	1.671(7)	1.645(3)	1.670(6)	1.662(5)
C1-P2	1.638(3)	1.664(2)	1.648(7)	1.648(3)		
C32-P3	1.634(3)	1.665(5)	1.640(6)	1.655(3)		
C32-P4	1.628(3)	1.663(4)	1.664(6)	1.643(3)		
P1-N1	1.619(2)	1.6128(18)	1.616(5)	1.635(2)	1.627(11)	1.621(10)
P2-N2	1.620(2)	1.6174(19)	1.633(5)	1.639(2)		
P3-N3	1.609(2)	1.6247(18)	1.630(5)	1.640(2)		
P4-N4	1.620(3)	1.6202(18)	1.629(5)	1.634(2)		
M1-C1	2.605(3)	2.385(2)	2.410(6)	2.513(2)	2.412(14)	2.489(14)
M1-C32	2.600(3)	2.399(3)	2.421(6)	2.516(2)		
M1-N1	2.576(2)	2.4766(17)	2.433(5)	2.469(2)	2.288(10)	2.373(10)
M1-N2	2.549(2)	2.5122(17)	2.468(5)	2.492(2)		
M1-N3	2.578(2)	2.4726(18)	2.440(5)	2.494(2)		
M1-N4	2.571(2)	2.4966(16)	2.478(5)	2.469(2)		
P1-C1-P2	164.41(18)	164.31(15)	164.8(4)	167.92(18)	142.5(10)	143.6(9)
P3-C32-P4	171.37(19)	163.61(14)	164.2(4)	165.44(17)		
N1-M1-N2	122.58(7)	127.16(6)	126.62(17)	124.78(7)	127.5(5)	123.6(5)
N3-M1-N4	122.58(7)	127.36(6)	126.68(17)	124.61(7)		
C1-M1-C32	172.39(8)	176.98(7)	177.5(2)	176.27(7)		

	**1Tb**	**2Pr**	**2Tb**	**6Pr**	**6Tb**
C1-P1	1.6451(19)	1.628(4)	1.633(5)	1.688(3)	1.694(3)
C1-P2	1.6411(19)	1.648(4)	1.641(5)	1.695(3)	1.692(3)
C32-P3	1.7351(18)	1.632(4)	1.644(4)	1.697(3)	1.700(3)
C32-P4	1.7323(19)	1.628(4)	1.635(4)	1.710(3)	1.706(3)
P1-N1	1.6207(16)	1.618(3)	1.618(3)	1.617(3)	1.623(2)
P2-N2	1.6293(15)	1.611(3)	1.615(4)	1.613(3)	1.621(2)
P3-N3	1.6039(16)	1.618(3)	1.618(3)	1.625(3)	1.632(2)
P4-N4	1.6086(16)	1.618(3)	1.616(3)	1.612(3)	1.608(2)
M1-C1	2.3848(17)	2.579(4)	2.470(4)	2.683(3)	2.601(3)
M1-C32	2.9222(18)	2.572(4)	2.460(4)	2.494(3)	2.397(3)
M1-N1	2.4877(15)	2.579(3)	2.485(3)	2.495(3)	2.420(2)
M1-N2	2.4646(15)	2.540(3)	2.475(3)	2.468(3)	2.386(2)
M1-N3	2.4182(15)	2.567(3)	2.492(3)	2.411(3)	2.347(2)
M1-N4	2.4002(15)	2.564(3)	2.495(3)		
P1-C1-P2	163.71(13)	167.3(3)	167.9(3)	138.25(19)	2.347(2)
P3-C32-P4	138.12(11)	170.8(3)	168.3(3)	127.60(19)	128.14(17)
N1-M1-N2	129.05(5)	122.67(10)	127.62(11)	116.22(9)	117.81(8)
N3-M1-N4	109.86(5)	123.44(10)	127.60(11)		
C1-M1-C32	158.50(6)	172.45(12)	176.78(8)	108.76(9)	111.38(9)

Supplementary Table 3. Final coordinates and energy for the single point energy calculation on the final geometry optimised coordinates of 3Ce

1.C	2.819088	2.172468	-5.975818
2.C	-2.755545	-2.761718	-5.607697
3.C	3.251763	3.360833	-5.375865
4.C	2.323003	1.128185	-5.190224
5.C	-3.602704	3.400526	-5.056211
6.C	2.896192	-3.919102	-5.053657
7.C	-2.854765	-3.973525	-4.915264
8.C	-2.214597	3.274754	-4.949834
9.C	-2.481885	-1.576984	-4.917879
10.C	1.543994	-3.675107	-4.802505
11.C	3.858346	-2.987854	-4.640073
12.C	-4.435609	2.445852	-4.457589
13.C	-1.658909	2.212279	-4.227117
14.C	3.174029	3.503451	-3.986177
15.C	1.155774	-2.515546	-4.123005
16.C	3.466473	-1.828449	-3.967661
17.C	-3.878302	1.382357	-3.744773
18.C	2.257057	1.257461	-3.791472
19.C	2.109316	-1.588218	-3.686241
20.C	-2.686291	-3.994611	-3.525838
21.C	-2.483905	1.265384	-3.605131
22.C	-2.302815	-1.591564	-3.523835
23.C	2.679161	2.458541	-3.202523
24.C	-2.413234	-2.811275	-2.838534
25.C	-0.073760	0.001777	-2.459617
26.C	5.161624	0.752540	-1.599705
27.C	-0.293828	3.828803	-1.517698
28.C	-5.067658	-1.344602	-1.238160
29.C	0.646296	-3.915073	-1.003672
30.C	4.401260	-1.983162	-0.490384
31.C	-4.718036	1.548499	-0.332744
32.C	2.193561	4.383621	0.194792
33.C	-1.874155	-4.437453	0.653392
34.C	-0.592122	5.315525	1.078270
35.C	3.847309	0.578995	1.118876
36.C	-3.735402	-0.740596	1.414757
37.C	0.902216	-5.069237	1.772926
38.C	0.063168	0.165159	2.409258
39.C	-2.577668	2.732904	2.559134
40.C	2.716200	-2.359165	2.985467
41.C	-3.786325	3.108388	3.147965
42.C	-1.434834	2.509302	3.342705
43.C	3.885757	-2.700961	3.666310
44.C	1.422623	2.494021	3.602846
45.C	1.541467	-2.042845	3.684929
46.C	1.596371	3.871630	3.823455
47.C	-1.309052	-1.999454	3.866308
48.C	-1.580403	-3.353425	4.127406
49.C	2.275034	1.592456	4.254555
50.C	-3.868461	3.264883	4.536032

51.C	-2.081197	-1.019741	4.504345
52.C	2.609366	4.334895	4.665852
53.C	-1.522761	2.678789	4.735396
54.C	-2.616625	-3.716686	4.990911
55.C	3.896514	-2.732034	5.065390
56.C	1.560970	-2.075982	5.090667
57.C	3.282948	2.053395	5.108065
58.C	-2.733412	3.052404	5.326872
59.C	3.454941	3.424813	5.313257
60.C	-3.110657	-1.380279	5.379733
61.C	-3.385463	-2.729226	5.619559
62.C	2.731333	-2.418970	5.774499
63.H	2.880315	2.050710	-7.058601
64.H	-2.890549	-2.736965	-6.690455
65.H	3.653905	4.169548	-5.988166
66.H	-4.037667	4.237017	-5.606320
67.H	3.202370	-4.829381	-5.572445
68.H	-3.064906	-4.898088	-5.455437
69.H	2.002031	0.200912	-5.667157
70.H	-1.558938	4.008123	-5.421100
71.H	-2.402066	-0.637806	-5.467545
72.H	4.915167	-3.165956	-4.842269
73.H	0.783690	-4.387597	-5.124578
74.H	-5.519670	2.530599	-4.546276
75.H	-0.576467	2.116612	-4.134897
76.H	0.100990	-2.329653	-3.925435
77.H	4.223510	-1.105547	-3.665892
78.H	3.511360	4.425565	-3.509721
79.H	-4.536095	0.639003	-3.296860
80.H	-2.766029	-4.934046	-2.976848
81.H	5.211416	0.476661	-2.662968
82.H	-5.177294	-1.171808	-2.318725
83.H	2.632279	2.563809	-2.120428
84.H	-0.093012	4.774865	-2.045076
85.H	0.167898	3.019398	-2.099749
86.H	-2.278426	-2.825881	-1.758853
87.H	5.012697	1.840999	-1.554539
88.H	0.126053	-3.249919	-1.706785
89.H	4.467965	-2.455593	-1.481431
90.H	-1.381222	3.663932	-1.532430
91.H	0.571686	-4.937544	-1.406755
92.H	6.147148	0.535822	-1.154919
93.H	-5.063584	1.875592	-1.323219
94.H	-4.757260	-2.391823	-1.109208
95.H	-6.065919	-1.238674	-0.781567
96.H	1.708969	-3.631099	-1.012096
97.H	2.331687	5.314716	-0.378246
98.H	5.395979	-2.042961	-0.019590
99.H	2.799869	3.601023	-0.283227
100.H	-4.049477	2.331248	0.050901
101.H	-1.920623	-5.408494	0.134171
102.H	3.708472	-2.591085	0.108432

103.H	-5.596608	1.501317	0.331575
104.H	-2.523630	-3.735098	0.112249
105.H	-0.433606	6.233064	0.487094
106.H	2.606364	4.541457	1.202154
107.H	-1.673657	5.116869	1.096975
108.H	3.669928	1.663659	1.080787
109.H	0.750876	-6.075103	1.346614
110.H	-2.312176	-4.570856	1.653123
111.H	-3.266591	-1.732960	1.484105
112.H	4.818787	0.415421	1.611545
113.H	-2.507841	2.607142	1.479845
114.H	1.980683	-4.857070	1.741931
115.H	3.073997	0.145697	1.770249
116.H	-4.735701	-0.809910	1.871476
117.H	-0.283755	5.538068	2.109349
118.H	-3.142194	-0.049479	2.031206
119.H	2.704170	-2.340242	1.897503
120.H	-4.664841	3.274771	2.523135
121.H	0.614328	-5.115222	2.832921
122.H	4.787137	-2.948436	3.103773
123.H	0.932622	4.589399	3.344751
124.H	-0.980008	-4.131811	3.660095
125.H	2.142375	0.522437	4.094632
126.H	-1.877265	0.032375	4.305736
127.H	2.735075	5.407139	4.823577
128.H	-4.813309	3.553312	4.998954
129.H	-2.823883	-4.771578	5.176556
130.H	-0.643888	2.514811	5.360918
131.H	4.807590	-3.005498	5.600610
132.H	3.931336	1.334364	5.610707
133.H	0.657746	-1.837351	5.654224
134.H	-3.700934	-0.602569	5.866429
135.H	4.241380	3.785282	5.978774
136.H	-4.194604	-3.013184	6.294871
137.H	-2.789129	3.178186	6.409327
138.H	2.729188	-2.447050	6.865265
139.Ce	0.021249	0.021161	-0.018814
140.N	2.206137	-0.025357	-1.223185
141.N	-2.259095	-0.120655	-1.049909
142.N	0.121370	2.321037	0.961042
143.N	0.031899	-2.158857	1.253415
144.P	1.577653	-0.098453	-2.739361
145.P	-1.740879	-0.093598	-2.608078
146.P	0.077015	1.837353	2.528329
147.P	0.057215	-1.477346	2.744187
148.Si	3.840967	-0.175389	-0.604048
149.Si	-3.878264	-0.154390	-0.361115
150.Si	0.359567	3.904312	0.240826
151.Si	-0.078191	-3.827327	0.726685

Supplementary Table 4. Final coordinates and energy for the single point energy calculation on the final geometry optimised coordinates of 3U

1.H	-5.510519	0.612827	-5.772337
2.H	4.959356	-0.135514	-5.738320
3.H	-3.033526	0.300353	-5.687217
4.H	0.056060	-1.021524	-5.692770
5.H	5.817491	2.123610	-5.109606
6.C	-4.969597	0.767000	-4.837026
7.H	1.067309	-3.655578	-5.008005
8.C	4.748087	0.274393	-4.749518
9.H	1.534721	-0.268491	-5.048416
10.C	-3.581207	0.593543	-4.790437
11.C	0.516394	-0.592797	-4.787695
12.C	5.234359	1.539924	-4.394844
13.H	-0.047832	0.311749	-4.518992
14.H	-1.459108	3.213010	-4.230458
15.H	-6.740235	1.294020	-3.709515
16.H	3.636857	-1.455933	-4.122362
17.H	2.547366	-3.258658	-4.110185
18.C	1.460700	-3.392402	-4.012074
19.H	-0.972465	4.868158	-3.808714
20.C	-5.659075	1.149131	-3.681011
21.C	3.993703	-0.467004	-3.837779
22.H	-1.667550	-2.913813	-3.954784
23.C	-2.891363	0.807477	-3.595787
24.H	-1.811210	0.684019	-3.559550
25.C	-1.459756	3.957682	-3.420992
26.H	1.299635	-4.260638	-3.355952
27.C	4.972652	2.048921	-3.120964
28.Si	0.520183	-1.868393	-3.383882
29.H	-2.510226	4.209078	-3.214979
30.H	1.227661	2.159228	-3.318413
31.H	5.349856	3.029757	-2.828196
32.H	1.733080	3.832846	-2.992596
33.C	-1.242455	-2.439836	-3.055621
34.H	-5.720456	-2.857501	-2.773075
35.H	-1.908018	-1.612884	-2.771712
36.C	-4.966184	1.360926	-2.484975
37.C	3.704167	0.049185	-2.561780
38.C	1.253067	2.945902	-2.549762
39.C	-3.570652	1.195266	-2.431221
40.C	4.213849	1.304947	-2.211193
41.H	-6.221632	-5.095399	-1.785706
42.H	-1.274865	-3.178780	-2.240841
43.C	-5.242692	-3.168026	-1.842870
44.H	-5.514415	1.666361	-1.592730
45.Si	-0.470470	3.348739	-1.922134
46.H	6.824263	-3.669673	-1.295220
47.N	1.137647	-1.187049	-1.885145
48.H	1.906617	2.588168	-1.742137
49.H	5.569868	-1.546209	-1.510198
50.H	-4.126891	-1.327033	-1.639141

51.C	5.737168	-3.673457	-1.201062
52.C	-5.523080	-4.419831	-1.288565
53.H	5.607246	-5.813334	-0.902760
54.C	5.028679	-2.474463	-1.322211
55.C	5.054480	-4.875352	-0.979530
56.H	-3.373739	4.240457	-1.335266
57.H	4.011156	1.704527	-1.218875
58.C	-4.345955	-2.303282	-1.206514
59.C	3.625323	-2.462906	-1.227297
60.H	0.255911	5.605960	-1.126238
61.P	2.671770	-0.889858	-1.358079
62.C	3.659650	-4.869576	-0.866743
63.C	2.953827	-3.670485	-0.989323
64.N	-1.093194	1.891861	-1.167597
65.H	3.118631	-5.801344	-0.695727
66.H	1.868616	-3.665531	-0.916002
67.C	-0.347370	4.791424	-0.693367
68.P	-2.626476	1.384911	-0.856886
69.H	-1.332795	5.205916	-0.435122
70.C	-3.864742	3.910306	-0.421541
71.H	0.043566	-4.585673	-0.547977
72.C	-4.908103	-4.801560	-0.089338
73.H	-4.811359	5.821465	-0.095138
74.H	3.285884	5.468167	-0.109437
75.H	5.760200	5.443018	0.228416
76.H	-5.124072	-5.775838	0.352028
77.C	-3.725082	-2.675272	-0.006243
78.H	-1.509779	-5.159009	0.071969
79.C	-4.684443	4.802450	0.273521
80.C	-3.680217	2.597605	0.044733
81.C	3.772965	4.590015	0.316868
82.C	-2.375502	-0.050783	-0.025402
83.H	0.125809	4.485250	0.250814
84.C	5.159714	4.575486	0.507407
85.U	0.012635	-0.033175	-0.005246
86.C	2.404273	-0.106319	0.103537
87.C	-0.483044	-4.895453	0.366161
88.C	-4.022226	-3.931599	0.550138
89.H	1.931651	3.483889	0.518830
90.H	0.005767	-5.808263	0.743640
91.C	3.008576	3.477294	0.671094
92.H	6.851682	3.428500	1.216340
93.C	5.772029	3.445421	1.060349
94.P	-2.596239	-1.479883	0.824604
95.N	-1.054903	-2.006911	1.062496
96.C	-5.344550	4.388764	1.437676
97.C	-4.351727	2.191637	1.205124
98.H	-3.568934	-4.232491	1.492601
99.C	3.612485	2.335920	1.220032
100.C	5.004730	2.332087	1.415929
101.H	1.954868	-2.850896	1.433799
102.H	-5.984711	5.086275	1.981034

103.H	-4.214920	1.174745	1.572130
104.H	5.492531	1.455331	1.844070
105.P	2.598329	0.821182	1.489149
106.Si	-0.446339	-3.534074	1.687774
107.H	-5.449795	-1.540262	1.747484
108.C	-5.181763	3.079385	1.897533
109.H	3.991374	-1.753034	1.654842
110.H	-1.356837	3.270749	1.920902
111.N	1.050038	1.203696	1.899645
112.C	1.327950	-3.256170	2.239111
113.H	-5.692066	2.744691	2.801874
114.C	-3.422755	-1.226858	2.450687
115.C	-4.821226	-1.252448	2.591695
116.H	1.781442	-4.202829	2.573765
117.C	4.092215	-1.279891	2.632033
118.H	-1.993958	1.784226	2.649627
119.H	1.300330	4.431413	2.862575
120.H	-2.407710	-4.472407	3.051393
121.C	-1.335667	2.646695	2.826211
122.C	3.528108	-0.014417	2.840552
123.H	1.375126	-2.547669	3.079409
124.C	-1.359840	-4.185918	3.218252
125.Si	0.407775	2.112277	3.266254
126.H	5.198338	-2.923117	3.490461
127.H	-0.830644	-5.086868	3.571731
128.H	-1.775001	3.232849	3.649333
129.H	-1.552594	-0.842565	3.440513
130.C	1.382653	3.689648	3.670871
131.H	-6.500963	-0.922515	3.905377
132.C	-2.635450	-0.859520	3.552223
133.C	4.770509	-1.935020	3.666543
134.C	-5.414412	-0.903205	3.809228
135.H	2.455066	3.535213	3.858340
136.H	-1.348710	-3.452609	4.038021
137.C	3.681320	0.599894	4.095824
138.H	3.280737	1.597711	4.267832
139.H	0.948866	4.141972	4.578098
140.C	-3.226063	-0.515010	4.769120
141.C	-4.619436	-0.530922	4.899384
142.H	-0.098262	0.089918	4.697237
143.C	4.888363	-1.331007	4.921739
144.C	0.311623	1.094592	4.868872
145.C	4.347448	-0.055940	5.132785
146.H	1.286666	0.965737	5.358668
147.H	5.402220	-1.848053	5.734485
148.H	-2.597724	-0.227772	5.613183
149.H	-5.083052	-0.254714	5.847722
150.H	-0.353264	1.613151	5.578725
151.H	4.444215	0.427271	6.106427

Supplementary Table 5. Final coordinates and energy for the single point energy calculation on the final geometry optimised coordinates of 3Th

1.C	3.082637	2.543343	-5.499101
2.C	3.262037	3.849722	-5.025475
3.C	-0.449261	0.282705	-4.915063
4.C	1.462418	-2.097983	-4.784132
5.C	2.844173	1.502170	-4.600140
6.C	-5.706119	0.202051	-3.823027
7.C	-4.351595	0.541265	-3.919578
8.C	3.211146	4.103083	-3.651434
9.C	2.767166	1.751650	-3.218779
10.C	-1.136883	-2.162274	-3.230900
11.C	6.192806	-1.112446	-2.850036
12.C	2.964512	3.058094	-2.753189
13.C	6.028449	-2.490207	-2.664808
14.C	5.104402	-0.248683	-2.701169
15.C	-6.296421	0.058749	-2.561957
16.C	-3.597765	0.736414	-2.760647
17.C	4.768819	-2.998909	-2.328624
18.C	3.834080	-0.751095	-2.365312
19.C	3.681888	-2.133758	-2.180882
20.C	-4.512057	-4.074558	-1.923311
21.C	-4.295997	-5.444586	-1.751512
22.C	-3.106948	4.165374	-1.669420
23.C	-5.537414	0.247220	-1.402650
24.C	-4.176072	0.588851	-1.490575
25.C	-0.123781	3.856066	-1.363716
26.C	-3.808600	-3.150473	-1.144013
27.C	-3.382424	-5.887444	-0.786230
28.C	0.875034	-4.708430	-0.812855
29.C	2.325993	0.895488	-0.431753
30.C	-2.885802	-3.582075	-0.182016
31.C	-2.689138	-4.962091	-0.002528
32.C	-2.352472	-0.744215	0.405894
33.C	-5.054148	2.469163	1.059145
34.C	-1.855948	4.358663	1.123547
35.C	4.824905	-0.419974	1.200574
36.C	4.396421	3.593471	1.185163
37.C	2.464347	-3.043453	1.207056
38.C	-4.256565	1.332083	1.277545
39.C	4.702582	4.952915	1.293680
40.C	3.071389	3.145915	1.338177
41.C	3.689545	5.884357	1.553385
42.C	2.063622	4.089708	1.583824
43.C	5.918941	-1.069734	1.782648
44.C	2.367062	5.449241	1.692373
45.C	-5.931916	2.922752	2.046084
46.C	4.110046	0.551083	1.912618
47.C	0.396968	-4.996554	2.197403
48.C	-4.372982	0.646631	2.494489
49.C	-2.506892	-2.774716	2.539595
50.C	-3.803720	-3.239758	2.821718

51.C	6.311113	-0.751817	3.085589
52.C	-6.032587	2.236685	3.263314
53.C	4.525503	0.880716	3.215207
54.C	-5.255104	1.095878	3.483183
55.C	-1.618190	-2.565110	3.604751
56.C	5.613601	0.230683	3.800697
57.C	-1.075883	1.071124	3.722814
58.C	-4.194575	-3.491691	4.140252
59.C	1.375614	2.612144	4.582898
60.C	1.457896	-0.445409	4.573310
61.C	-2.006475	-2.814554	4.922675
62.C	-3.297400	-3.280886	5.194468
63.H	3.130955	2.336053	-6.569508
64.H	3.446920	4.664910	-5.727709
65.H	-1.049910	-0.211168	-5.696873
66.H	0.939942	-2.418469	-5.701061
67.H	0.323587	0.884369	-5.413335
68.H	2.725522	0.488102	-4.979242
69.H	2.386372	-1.587759	-5.091433
70.H	-6.300138	0.054798	-4.726795
71.H	-3.881705	0.657770	-4.897259
72.H	-1.106591	0.981430	-4.378881
73.H	1.761743	-3.007711	-4.243187
74.H	-1.584656	-2.639524	-4.117577
75.H	3.360584	5.115668	-3.274125
76.H	7.170171	-0.707519	-3.116667
77.H	6.877635	-3.165006	-2.787192
78.H	5.242834	0.823317	-2.849700
79.H	-7.353741	-0.197869	-2.478603
80.H	-5.225758	-3.717700	-2.667043
81.H	-4.839943	-6.166854	-2.363167
82.H	-2.546309	1.010312	-2.830629
83.H	-1.943158	-1.617118	-2.717903
84.H	-0.798602	-2.964325	-2.559129
85.H	-3.169551	3.626209	-2.626136
86.H	4.630351	-4.071732	-2.187610
87.H	-0.092950	3.485828	-2.399260
88.H	-2.843484	5.212581	-1.894743
89.H	2.924082	3.248786	-1.680238
90.H	2.701541	-2.530736	-1.924379
91.H	1.172923	-4.056581	-1.647000
92.H	0.025197	4.947065	-1.396872
93.H	-3.971427	-2.081344	-1.279836
94.H	-4.113266	4.176838	-1.227876
95.H	-3.213519	-6.955552	-0.640345
96.H	-0.083599	-5.171555	-1.089846
97.H	-6.008706	0.131369	-0.425427
98.H	0.738120	3.424045	-0.834619
99.H	1.626086	-5.510796	-0.729313
100.H	-5.002804	2.995221	0.107416
101.H	4.523454	-0.663416	0.182122
102.H	-1.996749	-5.321760	0.756377

103.H	2.786851	-2.282716	0.480973
104.H	-1.722898	5.442583	0.975009
105.H	5.194978	2.874756	0.995276
106.H	6.460963	-1.824915	1.212046
107.H	5.737195	5.283876	1.187691
108.H	3.221921	-3.843192	1.192229
109.H	-2.822307	4.201931	1.625411
110.H	-6.543645	3.807467	1.863100
111.H	3.931586	6.944145	1.652910
112.H	1.036825	3.749068	1.701372
113.H	-1.071372	4.015735	1.813479
114.H	1.571035	6.167047	1.896427
115.H	-0.598795	-5.459489	2.157301
116.H	-4.509587	-3.411490	2.007597
117.H	1.139600	-5.808955	2.130379
118.H	2.494824	-2.578666	2.203840
119.H	-3.772514	-0.248231	2.658004
120.H	0.497882	-4.538460	3.192047
121.H	-1.523919	0.224174	3.181571
122.H	7.160896	-1.262074	3.543050
123.H	-1.497631	1.993042	3.296224
124.H	-0.612628	-2.209292	3.390500
125.H	-6.722553	2.587322	4.033488
126.H	4.002727	1.655041	3.774830
127.H	0.878269	3.520657	4.213430
128.H	1.191934	-1.381430	4.061858
129.H	-5.334432	0.549023	4.423460
130.H	-5.201507	-3.859721	4.343617
131.H	2.458186	2.798159	4.550173
132.H	5.920048	0.492186	4.814725
133.H	-1.415040	1.002184	4.768488
134.H	2.553630	-0.431491	4.669377
135.H	1.093801	2.490930	5.642242
136.H	1.028655	-0.480141	5.587644
137.H	-1.298180	-2.650698	5.736199
138.H	-3.601112	-3.484549	6.223144
139.N	0.982770	-0.356738	-2.294888
140.N	-1.824477	1.701959	-0.330616
141.N	-0.350412	-2.392679	0.712522
142.N	1.234983	1.028843	1.934879
143.Si	0.272447	-1.035421	-3.757509
144.Si	-1.763635	3.450169	-0.537149
145.Si	0.763021	-3.747780	0.817345
146.Si	0.804418	1.069274	3.639719
147.P	2.430918	0.395354	-2.018680
148.P	-3.093560	0.692663	-0.000257
149.P	-1.998904	-2.318912	0.825704
150.P	2.647948	1.362007	1.137151
151.Th	0.006361	0.013349	-0.000105

Supplementary Table 6. Final coordinates and energy for the single point energy calculation on the final geometry optimised coordinates of 3Ce with 5p core

1.C	2.633806	2.219720	-6.020314
2.C	-2.765773	-2.765730	-5.680485
3.C	3.084831	3.405958	-5.428843
4.C	2.204349	1.155958	-5.222342
5.C	-3.515919	3.328321	-5.199161
6.C	2.993612	-3.848741	-5.158260
7.C	-2.953797	-3.970146	-4.993098
8.C	-2.132705	3.217393	-5.030769
9.C	-2.439989	-1.600227	-4.980859
10.C	1.638229	-3.648630	-4.884721
11.C	3.936571	-2.909888	-4.719039
12.C	-4.365867	2.379482	-4.615116
13.C	-1.599161	2.171706	-4.268301
14.C	3.095176	3.525359	-4.034725
15.C	1.226010	-2.522869	-4.163233
16.C	3.521089	-1.782573	-4.007307
17.C	-3.830160	1.333101	-3.861547
18.C	2.221257	1.263923	-3.820040
19.C	2.160785	-1.583211	-3.710938
20.C	-2.813337	-4.003096	-3.600860
21.C	-2.441445	1.226546	-3.668617
22.C	-2.298440	-1.623814	-3.581941
23.C	2.664858	2.460773	-3.238618
24.C	-2.488403	-2.837748	-2.903735
25.C	-0.058882	-0.055049	-2.496093
26.C	5.195554	0.721653	-1.602031
27.C	-0.284194	3.804658	-1.482243
28.C	-5.119553	-1.260748	-1.251991
29.C	0.573063	-3.921412	-0.987871
30.C	4.373972	-2.001603	-0.474577
31.C	-4.619278	1.636627	-0.395895
32.C	2.184536	4.340108	0.248199
33.C	-1.928497	-4.381548	0.706028
34.C	-0.599941	5.329700	1.091821
35.C	3.858756	0.575349	1.106674
36.C	-3.730775	-0.673835	1.378549
37.C	0.856841	-5.105207	1.775397
38.C	0.067605	0.157660	2.472951
39.C	-2.582429	2.785038	2.676118
40.C	2.718340	-2.391325	3.053008
41.C	-3.764911	3.200266	3.291702
42.C	-1.437822	2.498727	3.435213
43.C	3.882215	-2.730136	3.746263
44.C	1.416635	2.503206	3.630585
45.C	1.546090	-2.043073	3.741375
46.C	1.544958	3.882034	3.874419
47.C	-1.306306	-2.021923	3.902062
48.C	-1.528633	-3.376416	4.205512
49.C	2.332104	1.622845	4.222115
50.C	-3.816592	3.337592	4.683500

51.C	-2.128933	-1.053430	4.491784
52.C	2.578051	4.368966	4.677765
53.C	-1.497977	2.639293	4.832842
54.C	-2.563239	-3.752085	5.065248
55.C	3.888672	-2.726638	5.146008
56.C	1.562153	-2.039399	5.147491
57.C	3.361781	2.106907	5.036509
58.C	-2.680142	3.056720	5.451327
59.C	3.490119	3.480354	5.262128
60.C	-3.158293	-1.425474	5.362824
61.C	-3.381656	-2.775473	5.647417
62.C	2.725811	-2.379979	5.843665
63.H	2.627822	2.116536	-7.106427
64.H	-2.877930	-2.730493	-6.765222
65.H	3.433253	4.231282	-6.052210
66.H	-3.932971	4.148171	-5.786920
67.H	3.316146	-4.730369	-5.715023
68.H	-3.213122	-4.878425	-5.539728
69.H	1.866234	0.231332	-5.692942
70.H	-1.462880	3.946680	-5.488754
71.H	-2.297089	-0.666317	-5.526527
72.H	4.996279	-3.054880	-4.935137
73.H	0.894143	-4.368198	-5.228922
74.H	-5.446369	2.453804	-4.748498
75.H	-0.520645	2.084338	-4.133875
76.H	0.168734	-2.369291	-3.949767
77.H	4.262412	-1.051005	-3.688393
78.H	3.446939	4.445336	-3.565824
79.H	-4.500281	0.591510	-3.427958
80.H	-2.962559	-4.936559	-3.056314
81.H	5.274259	0.413151	-2.654333
82.H	-5.254346	-1.075919	-2.327740
83.H	2.680237	2.548576	-2.153974
84.H	-0.038225	4.733709	-2.020225
85.H	0.160367	2.971813	-2.044722
86.H	-2.382264	-2.862720	-1.821072
87.H	5.040659	1.810280	-1.594687
88.H	0.058510	-3.239060	-1.678812
89.H	4.456344	-2.485729	-1.458659
90.H	-1.376076	3.680025	-1.518280
91.H	0.458430	-4.939938	-1.391292
92.H	6.171038	0.522888	-1.127388
93.H	-4.874285	1.998461	-1.401973
94.H	-4.832241	-2.316571	-1.139496
95.H	-6.102279	-1.133080	-0.768140
96.H	1.643522	-3.672214	-1.018835
97.H	2.337834	5.287655	-0.292819
98.H	5.352658	-2.080581	0.025556
99.H	2.770079	3.562345	-0.262779
100.H	-3.927901	2.367645	0.045740
101.H	-2.009363	-5.373997	0.233818
102.H	3.648864	-2.584152	0.111306

103.H	-5.540544	1.637320	0.209182
104.H	-2.544145	-3.681365	0.123478
105.H	-0.404868	6.242146	0.503292
106.H	2.605346	4.453641	1.258083
107.H	-1.685959	5.155538	1.081812
108.H	3.668942	1.657742	1.072590
109.H	0.667487	-6.106198	1.352559
110.H	-2.377775	-4.447349	1.707676
111.H	-3.295690	-1.680259	1.459136
112.H	4.836336	0.423463	1.590556
113.H	-2.532726	2.683702	1.593464
114.H	1.939767	-4.921799	1.718085
115.H	3.097874	0.131151	1.764812
116.H	-4.727294	-0.703760	1.847034
117.H	-0.313079	5.547311	2.130226
118.H	-3.109579	0.004651	1.980868
119.H	2.709309	-2.400692	1.964942
120.H	-4.643836	3.419301	2.683611
121.H	0.594321	-5.144983	2.842221
122.H	4.782096	-3.003449	3.193522
123.H	0.829232	4.581088	3.444470
124.H	-0.888780	-4.145663	3.776632
125.H	2.230893	0.551727	4.046595
126.H	-1.961446	-0.001253	4.262563
127.H	2.668343	5.442121	4.853284
128.H	-4.737279	3.666474	5.168339
129.H	-2.728187	-4.808059	5.285406
130.H	-0.617231	2.425270	5.440564
131.H	4.794258	-2.998938	5.690975
132.H	4.061346	1.405336	5.492911
133.H	0.660716	-1.774244	5.702162
134.H	-3.786834	-0.655372	5.812642
135.H	4.294196	3.859807	5.895323
136.H	-4.188377	-3.068007	6.322035
137.H	-2.711751	3.167469	6.536273
138.H	2.720596	-2.380305	6.934727
139.Ce	0.016931	0.010467	-0.008778
140.N	2.227531	-0.009569	-1.246795
141.N	-2.262594	-0.156096	-1.104718
142.N	0.068327	2.324640	1.022438
143.N	0.052910	-2.171934	1.301545
144.P	1.595622	-0.109063	-2.757052
145.P	-1.724210	-0.134905	-2.653377
146.P	0.056625	1.828741	2.585039
147.P	0.064812	-1.486735	2.790149
148.Si	3.852950	-0.184031	-0.615148
149.Si	-3.873303	-0.107094	-0.406600
150.Si	0.342945	3.893238	0.287584
151.Si	-0.114267	-3.829829	0.760637

Supplementary Table 7. Final coordinates and energy for the single point energy calculation on the final geometry optimised coordinates of 3U with 6p core

1.H	-5.425068	0.254749	-5.835376
2.H	5.680890	-0.963513	-5.368073
3.H	-3.032436	0.960512	-5.892781
4.H	0.158164	-0.330221	-5.635042
5.H	6.071721	1.502159	-5.317356
6.C	-4.896085	0.476502	-4.906802
7.H	0.956522	-3.263138	-5.327817
8.C	5.209453	-0.349221	-4.599366
9.H	1.712796	0.143341	-4.905433
10.C	-3.556020	0.872490	-4.939727
11.C	0.669007	-0.124358	-4.680939
12.C	5.429686	1.033475	-4.569191
13.H	0.192616	0.753557	-4.220696
14.H	-1.327988	3.762619	-4.002021
15.H	-6.605541	0.057864	-3.644494
16.H	4.252029	-2.030525	-3.654560
17.H	2.507366	-2.904609	-4.548840
18.C	1.440982	-3.081406	-4.353330
19.H	-0.920327	5.321254	-3.259227
20.C	-5.560011	0.367438	-3.677128
21.C	4.393031	-0.949697	-3.638123
22.H	-1.644536	-2.399043	-4.364023
23.C	-2.882089	1.160357	-3.747785
24.H	-1.837063	1.462295	-3.765512
25.C	-1.411908	4.350590	-3.076263
26.H	1.350955	-4.007150	-3.766251
27.C	4.835593	1.808358	-3.568273
28.Si	0.559678	-1.619688	-3.526602
29.H	-2.476420	4.554410	-2.897706
30.H	1.389368	2.700730	-3.002932
31.H	5.010981	2.884188	-3.527629
32.H	1.672613	4.348345	-2.394692
33.C	-1.242526	-2.135309	-3.372984
34.H	-4.978074	-3.536396	-2.926721
35.H	-1.862269	-1.319645	-2.975685
36.C	-4.886390	0.658875	-2.489932
37.C	3.774147	-0.173884	-2.640221
38.C	1.267835	3.358535	-2.130544
39.C	-3.538361	1.059963	-2.515170
40.C	4.014476	1.206453	-2.609823
41.H	-6.051774	-5.322883	-1.550504
42.H	-1.365807	-3.007981	-2.715867
43.C	-4.810993	-3.575825	-1.849387
44.H	-5.410968	0.577448	-1.536875
45.Si	-0.534106	3.518322	-1.615518
46.H	6.615077	-3.646661	-0.042875
47.N	1.135422	-1.225995	-1.909096
48.H	1.886265	2.959833	-1.314471
49.H	5.423548	-1.538736	-0.568589
50.H	-3.537724	-1.831780	-1.841077

51.C	5.570305	-3.678822	-0.354958
52.C	-5.412554	-4.574780	-1.077806
53.H	5.432595	-5.836517	-0.236726
54.C	4.898554	-2.491586	-0.650400
55.C	4.905349	-4.907993	-0.462684
56.H	-4.226487	3.665427	-2.028696
57.H	3.562105	1.807992	-1.824132
58.C	-3.994958	-2.616504	-1.242071
59.C	3.551372	-2.516450	-1.053869
60.H	-0.125585	5.622921	-0.321636
61.P	2.667324	-0.935442	-1.378605
62.C	3.566336	-4.940658	-0.862502
63.C	2.894104	-3.748653	-1.153926
64.N	-1.117232	1.902811	-1.224106
65.H	3.041994	-5.893390	-0.949296
66.H	1.849720	-3.765873	-1.458179
67.C	-0.639313	4.669937	-0.117772
68.P	-2.652182	1.380523	-0.934254
69.H	-1.682186	4.897002	0.151351
70.C	-4.365998	3.649955	-0.947666
71.H	-0.182877	-4.220301	-0.774747
72.C	-5.204484	-4.602480	0.306778
73.H	-5.644806	5.386580	-0.960337
74.H	3.075128	5.864065	0.986447
75.H	5.470653	5.794413	0.292138
76.H	-5.682698	-5.368978	0.918482
77.C	-3.766150	-2.644644	0.140338
78.H	-1.706964	-4.904265	-0.173933
79.C	-5.176463	4.615735	-0.346406
80.C	-3.751179	2.649075	-0.172437
81.C	3.592993	4.907993	0.897013
82.C	-2.426143	-0.001133	-0.010916
83.H	-0.163594	4.206093	0.758669
84.C	4.934952	4.868270	0.507647
85.U	0.003615	-0.007180	-0.005344
86.C	2.433612	-0.011851	0.001550
87.C	-0.664052	-4.682467	0.099538
88.C	-4.392329	-3.640869	0.912486
89.H	1.862981	3.742346	1.471417
90.H	-0.155949	-5.638142	0.304813
91.C	2.909747	3.719286	1.175771
92.H	6.638749	3.596913	0.092856
93.C	5.591738	3.635028	0.396910
94.P	-2.663905	-1.383166	0.909092
95.N	-1.133560	-1.914023	1.205963
96.C	-5.394818	4.587504	1.036624
97.C	-3.990031	2.620676	1.208193
98.H	-4.261123	-3.655257	1.994582
99.C	3.558596	2.482977	1.072677
100.C	4.908621	2.450989	0.678772
101.H	1.863295	-2.982864	1.316747
102.H	-6.031925	5.339521	1.505877

103.H	-3.541136	1.832822	1.809354
104.H	5.427605	1.494928	0.595735
105.P	2.663246	0.905926	1.386369
106.Si	-0.561277	-3.532731	1.598913
107.H	-5.421027	-0.565078	1.499583
108.C	-4.804766	3.583927	1.811089
109.H	3.535690	-1.844202	1.831970
110.H	-1.367302	2.994785	2.700800
111.N	1.129400	1.202883	1.907773
112.C	1.237568	-3.381923	2.126951
113.H	-4.978684	3.544720	2.887352
114.C	-3.556258	-1.061347	2.486197
115.C	-4.902043	-0.653444	2.455145
116.H	1.636945	-4.373979	2.390854
117.C	3.987841	-1.247644	2.621526
118.H	-1.875167	1.309642	2.959928
119.H	1.348055	3.979572	3.774337
120.H	-2.517204	-4.559919	2.867374
121.C	-1.252541	2.122065	3.359423
122.C	3.757854	0.134445	2.653059
123.H	1.355177	-2.727211	3.002225
124.C	-1.452365	-4.363508	3.052384
125.Si	0.545986	1.596316	3.522635
126.H	4.965250	-2.935139	3.542027
127.H	-0.967961	-5.337973	3.234459
128.H	-1.657776	2.389286	4.348207
129.H	-1.863956	-1.477013	3.744081
130.C	1.430697	3.051203	4.358274
131.H	-6.624436	-0.048676	3.602809
132.C	-2.907380	-1.169997	3.721955
133.C	4.798175	-1.857926	3.583798
134.C	-5.580700	-0.363748	3.639866
135.H	2.495220	2.868414	4.558372
136.H	-1.369619	-3.779513	3.980741
137.C	4.376725	0.903230	3.656528
138.H	4.244007	1.985094	3.674674
139.H	0.942405	3.231734	5.331057
140.C	-3.586336	-0.884007	4.911292
141.C	-4.924140	-0.481161	4.872670
142.H	0.164490	-0.776555	4.208968
143.C	5.392360	-1.089722	4.589787
144.C	0.641907	0.098128	4.674186
145.C	5.182859	0.294527	4.621306
146.H	1.683344	-0.174359	4.903842
147.H	6.025824	-1.564881	5.341109
148.H	-3.068233	-0.977807	5.866723
149.H	-5.456882	-0.259987	5.799136
150.H	0.126547	0.303897	5.625854
151.H	5.654454	0.903571	5.394080

Supplementary Table 8. Final coordinates and energy for the single point energy calculation on the final geometry optimised coordinates of 3Th with 6p core

1.C	3.105888	2.559729	-5.492482
2.C	3.279416	3.865206	-5.014353
3.C	-0.432819	0.316818	-4.904498
4.C	1.453768	-2.090337	-4.796009
5.C	2.870882	1.514491	-4.597319
6.C	-5.735538	0.213405	-3.818916
7.C	-4.385119	0.567721	-3.914817
8.C	3.226327	4.114004	-3.639716
9.C	2.792266	1.759698	-3.215034
10.C	-1.133992	-2.120649	-3.223845
11.C	6.215525	-1.119288	-2.845102
12.C	2.984379	3.065242	-2.744687
13.C	6.042842	-2.497721	-2.673645
14.C	5.131526	-0.250834	-2.692590
15.C	-6.319032	0.040425	-2.558291
16.C	-3.628648	0.750372	-2.755530
17.C	4.779384	-3.002737	-2.346936
18.C	3.857267	-0.750269	-2.367830
19.C	3.696524	-2.133210	-2.195715
20.C	-4.519183	-4.096202	-1.927773
21.C	-4.305268	-5.465567	-1.747748
22.C	-3.108757	4.163873	-1.685655
23.C	-5.558161	0.218406	-1.398877
24.C	-4.201772	0.578319	-1.486405
25.C	-0.133438	3.824196	-1.364632
26.C	-3.817397	-3.168974	-1.150323
27.C	-3.393652	-5.904560	-0.778854
28.C	0.867268	-4.695121	-0.815331
29.C	2.367808	0.897834	-0.431957
30.C	-2.898935	-3.597051	-0.182588
31.C	-2.701453	-4.976607	0.002598
32.C	-2.393336	-0.760946	0.409350
33.C	-5.068418	2.468659	1.064927
34.C	-1.867803	4.346271	1.113872
35.C	4.850192	-0.412209	1.207940
36.C	4.412082	3.602137	1.157048
37.C	2.447042	-3.019504	1.198199
38.C	-4.275578	1.327960	1.283511
39.C	4.713670	4.963476	1.250610
40.C	3.090547	3.151196	1.328843
41.C	3.699948	5.893225	1.513147
42.C	2.081551	4.092756	1.577734
43.C	5.947050	-1.056768	1.790680
44.C	2.381097	5.454427	1.671257
45.C	-5.938179	2.929651	2.055552
46.C	4.131913	0.556782	1.919660
47.C	0.406298	-4.996062	2.200358
48.C	-4.387723	0.647312	2.503530
49.C	-2.533665	-2.782880	2.542796
50.C	-3.834876	-3.237282	2.822701

51.C	6.337047	-0.736624	3.093787
52.C	-6.034773	2.248819	3.276083
53.C	4.543844	0.887921	3.223165
54.C	-5.261653	1.104916	3.495663
55.C	-1.644100	-2.580809	3.608403
56.C	5.634306	0.242119	3.809022
57.C	-1.061436	1.065748	3.708258
58.C	-4.228092	-3.488299	4.140428
59.C	1.375051	2.614199	4.587594
60.C	1.466847	-0.445574	4.570446
61.C	-2.035085	-2.830629	4.925785
62.C	-3.329541	-3.287786	5.195434
63.H	3.155775	2.356095	-6.563422
64.H	3.460846	4.683597	-5.713698
65.H	-1.031130	-0.170799	-5.691643
66.H	0.921696	-2.395125	-5.712720
67.H	0.338839	0.926423	-5.394903
68.H	2.755753	0.500934	-4.980259
69.H	2.385802	-1.593976	-5.102819
70.H	-6.331558	0.076999	-4.722785
71.H	-3.920294	0.705993	-4.891832
72.H	-1.094268	1.006185	-4.360848
73.H	1.737277	-3.008272	-4.260417
74.H	-1.598284	-2.582564	-4.110068
75.H	3.370722	5.125993	-3.258979
76.H	7.196148	-0.717214	-3.104093
77.H	6.888520	-3.175843	-2.799427
78.H	5.276010	0.822005	-2.831141
79.H	-7.372836	-0.229921	-2.475489
80.H	-5.230691	-3.742470	-2.675065
81.H	-4.848664	-6.190670	-2.356542
82.H	-2.580307	1.035705	-2.825462
83.H	-1.929309	-1.570388	-2.698309
84.H	-0.801963	-2.935833	-2.564842
85.H	-3.153385	3.640845	-2.652030
86.H	4.634771	-4.075922	-2.216024
87.H	-0.098055	3.455182	-2.400461
88.H	-2.841025	5.214680	-1.888489
89.H	2.943672	3.252263	-1.671050
90.H	2.712851	-2.527580	-1.947484
91.H	1.157442	-4.035874	-1.646431
92.H	0.020985	4.914562	-1.397544
93.H	-3.980774	-2.100396	-1.290116
94.H	-4.122435	4.166056	-1.261013
95.H	-3.225484	-6.972051	-0.628399
96.H	-0.087573	-5.165868	-1.092374
97.H	-6.023354	0.080826	-0.421370
98.H	0.726078	3.391635	-0.831125
99.H	1.626319	-5.490461	-0.738216
100.H	-5.019353	2.992238	0.110893
101.H	4.549387	-0.656402	0.189391
102.H	-2.010921	-5.333961	0.765024

103.H	2.764562	-2.258723	0.469441
104.H	-1.732102	5.429475	0.962938
105.H	5.211370	2.884508	0.963259
106.H	6.493547	-1.809176	1.220649
107.H	5.745352	5.297549	1.129222
108.H	3.209631	-3.814574	1.181015
109.H	-2.833937	4.192917	1.617593
110.H	-6.546333	3.816882	1.872607
111.H	3.938715	6.954714	1.599711
112.H	1.057130	3.750105	1.709721
113.H	-1.082188	4.002485	1.802419
114.H	1.584946	6.171065	1.877899
115.H	-0.587437	-5.464320	2.167221
116.H	-4.541744	-3.402414	2.007517
117.H	1.153737	-5.803826	2.124654
118.H	2.481174	-2.554167	2.194383
119.H	-3.791767	-0.251075	2.666219
120.H	0.514201	-4.538069	3.194295
121.H	-1.510214	0.216547	3.170576
122.H	7.189558	-1.242273	3.551246
123.H	-1.486240	1.986178	3.281284
124.H	-0.635636	-2.231349	3.396256
125.H	-6.717408	2.606297	4.049308
126.H	4.017527	1.660432	3.783208
127.H	0.870945	3.519500	4.219505
128.H	1.189440	-1.380521	4.062575
129.H	-5.337924	0.561652	4.438440
130.H	-5.237821	-3.848703	4.342790
131.H	2.456607	2.808045	4.557166
132.H	5.938882	0.504716	4.823383
133.H	-1.401667	0.998403	4.753745
134.H	2.563012	-0.439248	4.660196
135.H	1.091513	2.485739	5.645867
136.H	1.042480	-0.474905	5.586991
137.H	-1.325984	-2.674230	5.739925
138.H	-3.635401	-3.492041	6.222947
139.N	1.012130	-0.350559	-2.299898
140.N	-1.854702	1.690112	-0.333327
141.N	-0.374698	-2.399995	0.723410
142.N	1.261348	1.037004	1.937672
143.Si	0.286879	-1.013209	-3.761761
144.Si	-1.782163	3.436688	-0.545136
145.Si	0.751777	-3.745206	0.819773
146.Si	0.819810	1.071512	3.639235
147.P	2.460945	0.400628	-2.018471
148.P	-3.122055	0.679982	0.003812
149.P	-2.026015	-2.329690	0.829928
150.P	2.674357	1.366062	1.137463
151.Th	0.006385	0.008440	0.001672

Supplementary Table 9. Final coordinates and energy for the single point energy calculation on the final geometry optimised coordinates of 3Ce with 5p valence

1.C	2.767232	2.186704	-5.984239
2.C	-2.780686	-2.751335	-5.626230
3.C	3.199850	3.376796	-5.387339
4.C	2.294235	1.135618	-5.193527
5.C	-3.583132	3.368239	-5.097603
6.C	2.930604	-3.891015	-5.088166
7.C	-2.899876	-3.964427	-4.939077
8.C	-2.195873	3.248576	-4.974764
9.C	-2.483893	-1.575247	-4.931065
10.C	1.576117	-3.653685	-4.843316
11.C	3.887603	-2.966209	-4.649103
12.C	-4.418960	2.418678	-4.495603
13.C	-1.644047	2.195910	-4.235518
14.C	3.147507	3.513465	-3.995620
15.C	1.179626	-2.506967	-4.146735
16.C	3.487557	-1.818513	-3.961868
17.C	-3.865472	1.364622	-3.766530
18.C	2.250367	1.259767	-3.793208
19.C	2.127974	-1.584588	-3.689460
20.C	-2.724244	-3.995551	-3.550658
21.C	-2.472432	1.252373	-3.614344
22.C	-2.301302	-1.598866	-3.537404
23.C	2.674946	2.461631	-3.207146
24.C	-2.427812	-2.820617	-2.858395
25.C	-0.067159	-0.011451	-2.464478
26.C	5.178226	0.737787	-1.603317
27.C	-0.289092	3.837147	-1.511096
28.C	-5.085540	-1.321244	-1.245673
29.C	0.634914	-3.937326	-0.995038
30.C	4.399280	-1.990045	-0.479006
31.C	-4.687968	1.570798	-0.346781
32.C	2.190386	4.374264	0.213040
33.C	-1.889457	-4.424836	0.662984
34.C	-0.596680	5.321741	1.082851
35.C	3.865009	0.582955	1.114418
36.C	-3.748835	-0.732089	1.406471
37.C	0.883625	-5.082940	1.784023
38.C	0.063611	0.162072	2.421081
39.C	-2.578447	2.756623	2.589811
40.C	2.715511	-2.370041	3.000378
41.C	-3.775990	3.152999	3.187904
42.C	-1.435028	2.508377	3.364502
43.C	3.882811	-2.714284	3.683924
44.C	1.418003	2.498112	3.603103
45.C	1.541146	-2.046322	3.697312
46.C	1.577458	3.876834	3.825760
47.C	-1.307987	-2.006190	3.872500
48.C	-1.570199	-3.360466	4.139935
49.C	2.286063	1.604687	4.244794
50.C	-3.845577	3.307190	4.577056

51.C	-2.088479	-1.029083	4.503826
52.C	2.592059	4.350483	4.660059
53.C	-1.511185	2.672126	4.758639
54.C	-2.604167	-3.727685	5.004024
55.C	3.891523	-2.741241	5.083146
56.C	1.558581	-2.075684	5.102973
57.C	3.296114	2.075631	5.090519
58.C	-2.710047	3.067643	5.359680
59.C	3.453800	3.448585	5.297764
60.C	-3.116483	-1.392825	5.379877
61.C	-3.380970	-2.742647	5.627440
62.C	2.726684	-2.421297	5.789706
63.H	2.811081	2.069269	-7.068030
64.H	-2.919868	-2.718088	-6.707772
65.H	3.584228	4.191096	-6.004102
66.H	-4.014544	4.195323	-5.664218
67.H	3.242397	-4.791064	-5.621495
68.H	-3.131650	-4.881828	-5.482601
69.H	1.972913	0.207116	-5.667681
70.H	-1.537621	3.978902	-5.447765
71.H	-2.391649	-0.634831	-5.476161
72.H	4.946750	-3.140076	-4.844764
73.H	0.820234	-4.361656	-5.185720
74.H	-5.502696	2.499372	-4.595808
75.H	-0.562362	2.103606	-4.132253
76.H	0.122900	-2.326216	-3.953450
77.H	4.240520	-1.099653	-3.641683
78.H	3.485300	4.436210	-3.521876
79.H	-4.525218	0.623559	-3.317810
80.H	-2.819110	-4.935789	-3.005894
81.H	5.235051	0.451921	-2.663367
82.H	-5.201407	-1.142653	-2.324526
83.H	2.645152	2.562971	-2.123971
84.H	-0.072703	4.781618	-2.035299
85.H	0.168123	3.023549	-2.091318
86.H	-2.289901	-2.843293	-1.779217
87.H	5.029474	1.826815	-1.570049
88.H	0.119235	-3.274577	-1.704243
89.H	4.477837	-2.466780	-1.466866
90.H	-1.378286	3.685398	-1.534891
91.H	0.550886	-4.963256	-1.387631
92.H	6.161333	0.525582	-1.150924
93.H	-5.009045	1.910554	-1.341155
94.H	-4.783136	-2.371742	-1.123631
95.H	-6.079694	-1.208832	-0.781921
96.H	1.700044	-3.663237	-1.007954
97.H	2.334343	5.310152	-0.350737
98.H	5.385857	-2.054426	0.007794
99.H	2.791246	3.592565	-0.273548
100.H	-4.008452	2.336197	0.052399
101.H	-1.945793	-5.402571	0.157540
102.H	3.692803	-2.590144	0.111528

103.H	-5.577755	1.539712	0.303189
104.H	-2.525733	-3.721565	0.107236
105.H	-0.421953	6.240186	0.497769
106.H	2.604057	4.519029	1.221919
107.H	-1.680015	5.132471	1.085143
108.H	3.681613	1.666680	1.073996
109.H	0.715995	-6.090064	1.366956
110.H	-2.335200	-4.537247	1.661919
111.H	-3.288924	-1.728402	1.479577
112.H	4.841075	0.426664	1.600382
113.H	-2.516615	2.636202	1.509423
114.H	1.964580	-4.884679	1.742754
115.H	3.098868	0.147565	1.773131
116.H	-4.751868	-0.792474	1.858547
117.H	-0.301584	5.537808	2.119048
118.H	-3.152683	-0.045277	2.025268
119.H	2.704347	-2.355014	1.912341
120.H	-4.654856	3.339880	2.569154
121.H	0.604409	-5.117248	2.846712
122.H	4.784282	-2.966536	3.123960
123.H	0.900897	4.587443	3.354679
124.H	-0.963795	-4.136741	3.677329
125.H	2.163903	0.533612	4.082732
126.H	-1.891941	0.023287	4.299617
127.H	2.706087	5.423808	4.819127
128.H	-4.781044	3.615180	5.047271
129.H	-2.802774	-4.783599	5.193544
130.H	-0.631063	2.489994	5.377399
131.H	4.800965	-3.016262	5.620148
132.H	3.957518	1.363405	5.585987
133.H	0.655265	-1.832395	5.664595
134.H	-3.713336	-0.616435	5.861046
135.H	4.242084	3.816543	5.957029
136.H	-4.188367	-3.028919	6.303836
137.H	-2.756029	3.191270	6.442750
138.H	2.723545	-2.446058	6.880571
139.Ce	0.021214	0.016288	-0.016124
140.N	2.217701	-0.024258	-1.224809
141.N	-2.260466	-0.136803	-1.059770
142.N	0.103511	2.323536	0.971613
143.N	0.038594	-2.167282	1.263900
144.P	1.585952	-0.100566	-2.737845
145.P	-1.734486	-0.106922	-2.613828
146.P	0.069380	1.834588	2.536585
147.P	0.059123	-1.481483	2.751985
148.Si	3.850926	-0.179075	-0.604874
149.Si	-3.879040	-0.146464	-0.371082
150.Si	0.353604	3.904692	0.252019
151.Si	-0.087291	-3.833735	0.736429

Supplementary Table 10. Final coordinates and energy for the single point energy calculation on the final geometry optimised coordinates of 3U with 6p valence

1.H	-5.502358	0.416081	-5.783642
2.H	5.222250	-0.616059	-5.658152
3.H	-3.029270	0.745458	-5.837113
4.H	-0.004180	-0.658081	-5.650987
5.H	6.063350	1.690395	-5.203460
6.C	-4.954949	0.612053	-4.859946
7.H	0.825576	-3.517048	-5.130808
8.C	4.933304	-0.098108	-4.742500
9.H	1.594936	-0.163799	-5.042056
10.C	-3.569313	0.796952	-4.890682
11.C	0.556743	-0.375565	-4.745834
12.C	5.406273	1.195699	-4.485787
13.H	0.131709	0.562495	-4.360341
14.H	-1.054137	3.499053	-4.182878
15.H	-6.721424	0.548731	-3.608993
16.H	3.759022	-1.749349	-4.019527
17.H	2.365248	-3.222480	-4.300985
18.C	1.279837	-3.301999	-4.148852
19.H	-0.775647	5.118952	-3.516803
20.C	-5.639731	0.687038	-3.639836
21.C	4.099041	-0.733403	-3.820506
22.H	-1.809474	-2.509181	-4.126415
23.C	-2.871288	1.053855	-3.706441
24.H	-1.792882	1.195224	-3.724623
25.C	-1.238057	4.139514	-3.307814
26.H	1.100219	-4.177469	-3.507774
27.C	5.042362	1.845108	-3.303066
28.Si	0.455187	-1.746607	-3.444174
29.H	-2.323037	4.298370	-3.237917
30.H	1.474006	2.440990	-3.038566
31.H	5.412621	2.848375	-3.088012
32.H	1.843556	4.092130	-2.494118
33.C	-1.345243	-2.210272	-3.173258
34.H	-5.381484	-3.150814	-2.873086
35.H	-1.929961	-1.372170	-2.771425
36.C	-4.941861	0.950245	-2.459518
37.C	3.712662	-0.079391	-2.635838
38.C	1.370651	3.142380	-2.197959
39.C	-3.546847	1.135300	-2.481591
40.C	4.199025	1.209946	-2.384997
41.H	-6.020641	-5.261890	-1.701604
42.H	-1.446396	-3.052419	-2.473312
43.C	-5.014515	-3.354211	-1.866181
44.H	-5.484377	1.017331	-1.515130
45.Si	-0.430922	3.426577	-1.746872
46.H	6.724100	-3.634649	-0.596718
47.N	1.094669	-1.213266	-1.897415
48.H	1.947082	2.738001	-1.355207
49.H	5.496827	-1.533378	-1.059309
50.H	-3.907730	-1.502243	-1.744811

51.C	5.640309	-3.657832	-0.720196
52.C	-5.372136	-4.534709	-1.209274
53.H	5.490620	-5.798428	-0.433184
54.C	4.947963	-2.473202	-0.979936
55.C	4.947344	-4.872097	-0.628012
56.H	-3.722722	4.018548	-1.770027
57.H	3.921775	1.713980	-1.460875
58.C	-4.181473	-2.424214	-1.234616
59.C	3.550706	-2.486066	-1.147278
60.H	0.047846	5.626584	-0.653527
61.P	2.631707	-0.909246	-1.396526
62.C	3.558815	-4.893348	-0.792820
63.C	2.866868	-3.705416	-1.048567
64.N	-1.084547	1.884844	-1.218705
65.H	3.012126	-5.835060	-0.725689
66.H	1.786456	-3.715407	-1.174818
67.C	-0.525374	4.727891	-0.374973
68.P	-2.623004	1.393060	-0.908656
69.H	-1.560789	5.036351	-0.167223
70.C	-4.071267	3.828856	-0.755283
71.H	-0.113448	-4.384925	-0.532284
72.C	-4.902811	-4.777518	0.088406
73.H	-5.183443	5.674439	-0.659651
74.H	3.116043	5.786917	0.708865
75.H	5.593011	5.693707	0.419848
76.H	-5.185077	-5.692245	0.611682
77.C	-3.698157	-2.661353	0.058586
78.H	-1.577115	-5.062180	0.196924
79.C	-4.905342	4.759989	-0.133476
80.C	-3.696891	2.646243	-0.090597
81.C	3.641128	4.833246	0.779808
82.C	-2.388693	-0.008753	-0.015547
83.H	-0.108955	4.335830	0.564368
84.C	5.028966	4.780380	0.617414
85.U	0.001375	-0.009732	-0.003213
86.C	2.390991	-0.032634	0.014320
87.C	-0.541779	-4.757207	0.409547
88.C	-4.078600	-3.843891	0.719946
89.H	1.841558	3.698115	1.162763
90.H	0.022517	-5.654730	0.709352
91.C	2.921727	3.662095	1.039038
92.H	6.777370	3.502649	0.593069
93.C	5.694319	3.551024	0.715332
94.P	-2.630276	-1.406416	0.881838
95.N	-1.093843	-1.897578	1.205649
96.C	-5.389530	4.515331	1.158412
97.C	-4.194440	2.407314	1.196875
98.H	-3.740745	-4.032891	1.738482
99.C	3.578041	2.428440	1.145132
100.C	4.975178	2.383934	0.980290
101.H	1.935328	-2.766474	1.382870
102.H	-6.044981	5.240854	1.643615

103.H	-3.925192	1.485450	1.709774
104.H	5.502627	1.432617	1.066529
105.P	2.628827	0.871358	1.407650
106.Si	-0.451008	-3.435270	1.761749
107.H	-5.503310	-1.081640	1.478274
108.C	-5.036714	3.335437	1.819171
109.H	3.928740	-1.742192	1.489402
110.H	-1.449582	3.058711	2.417190
111.N	1.091982	1.204729	1.889613
112.C	1.347953	-3.155619	2.225120
113.H	-5.414776	3.130827	2.821793
114.C	-3.564425	-1.140916	2.447236
115.C	-4.962544	-0.982868	2.420896
116.H	1.810810	-4.104477	2.539626
117.C	4.188673	-1.239197	2.419605
118.H	-1.941670	1.387735	2.751189
119.H	1.085215	4.195346	3.463379
120.H	-2.363568	-4.276566	3.243389
121.C	-1.353251	2.231557	3.135266
122.C	3.686078	0.044324	2.668356
123.H	1.447639	-2.443452	3.057109
124.C	-1.278851	-4.120310	3.324226
125.Si	0.443292	1.764021	3.422960
126.H	5.405030	-2.869560	3.137222
127.H	-0.822410	-5.097680	3.554821
128.H	-1.817694	2.553383	4.080891
129.H	-1.810185	-1.138044	3.691174
130.C	1.271366	3.325624	4.110494
131.H	-6.749847	-0.587863	3.561052
132.C	-2.891101	-1.017942	3.670146
133.C	5.023066	-1.869998	3.348924
134.C	-5.665718	-0.705510	3.595066
135.H	2.358178	3.250396	4.254679
136.H	-1.103958	-3.466306	4.191043
137.C	4.047184	0.696855	3.861612
138.H	3.693563	1.708135	4.059959
139.H	0.823652	3.548327	5.093678
140.C	-3.593820	-0.745741	4.847894
141.C	-4.983012	-0.588307	4.812981
142.H	0.101229	-0.530299	4.369142
143.C	5.363606	-1.221234	4.538990
144.C	0.521166	0.412964	4.747424
145.C	4.874508	0.066954	4.793321
146.H	1.552150	0.202687	5.068094
147.H	6.013963	-1.712640	5.265045
148.H	-3.055027	-0.660369	5.792684
149.H	-5.534626	-0.380108	5.731453
150.H	-0.057366	0.711271	5.636341
151.H	5.144007	0.583928	5.715493

Supplementary Table 11. Final coordinates and energy for the single point energy calculation on the final geometry optimised coordinates of 3Th with 6p valence

1.C	3.090178	2.545766	-5.493032
2.C	3.269553	3.851242	-5.017223
3.C	-0.445342	0.295210	-4.907273
4.C	1.454914	-2.093229	-4.783555
5.C	2.848402	1.503679	-4.596120
6.C	-5.711291	0.207616	-3.818455
7.C	-4.358131	0.551089	-3.916684
8.C	3.216107	4.102928	-3.643195
9.C	2.768418	1.751834	-3.214262
10.C	-1.140743	-2.147855	-3.227857
11.C	6.191567	-1.113185	-2.847528
12.C	2.967559	3.057237	-2.746253
13.C	6.025684	-2.491276	-2.667637
14.C	5.104348	-0.248660	-2.695262
15.C	-6.297443	0.052933	-2.556830
16.C	-3.600913	0.739738	-2.758797
17.C	4.765863	-3.000015	-2.332688
18.C	3.833520	-0.751789	-2.362081
19.C	3.679696	-2.134577	-2.181434
20.C	-4.510297	-4.077176	-1.923187
21.C	-4.296134	-5.446933	-1.746999
22.C	-3.102175	4.161960	-1.673758
23.C	-5.536044	0.236841	-1.398513
24.C	-4.176264	0.584362	-1.488331
25.C	-0.123091	3.845449	-1.366035
26.C	-3.805223	-3.151549	-1.146692
27.C	-3.382212	-5.888238	-0.781470
28.C	0.873017	-4.698288	-0.813535
29.C	2.326063	0.892522	-0.429965
30.C	-2.882793	-3.581714	-0.183611
31.C	-2.686927	-4.962034	-0.000850
32.C	-2.352462	-0.745203	0.405629
33.C	-5.052883	2.468219	1.058067
34.C	-1.856301	4.349755	1.119106
35.C	4.826538	-0.418392	1.200536
36.C	4.395351	3.590222	1.175161
37.C	2.463427	-3.032186	1.202809
38.C	-4.255182	1.330941	1.277939
39.C	4.702222	4.949628	1.280152
40.C	3.070495	3.143753	1.333884
41.C	3.690637	5.882043	1.540916
42.C	2.063677	4.088422	1.580406
43.C	5.924099	-1.063499	1.781541
44.C	2.368604	5.448082	1.685207
45.C	-5.930446	2.922421	2.044847
46.C	4.109378	0.550548	1.913518
47.C	0.402677	-4.988734	2.196683
48.C	-4.372132	0.645841	2.495065
49.C	-2.509855	-2.771546	2.538188
50.C	-3.808617	-3.234005	2.817146

51.C	6.316495	-0.743764	3.083909
52.C	-6.030856	2.237714	3.262793
53.C	4.525049	0.882313	3.215935
54.C	-5.254098	1.096572	3.483264
55.C	-1.622097	-2.564705	3.604458
56.C	5.616206	0.236136	3.799905
57.C	-1.075360	1.067884	3.717929
58.C	-4.201204	-3.487367	4.134724
59.C	1.371536	2.608166	4.580002
60.C	1.458789	-0.446883	4.568363
61.C	-2.012514	-2.817131	4.921465
62.C	-3.304540	-3.281640	5.190221
63.H	3.140897	2.339595	-6.563448
64.H	3.455970	4.667188	-5.718044
65.H	-1.042761	-0.198447	-5.691373
66.H	0.927703	-2.405324	-5.700564
67.H	0.328107	0.899037	-5.401955
68.H	2.729775	0.490019	-4.977254
69.H	2.381516	-1.586948	-5.090588
70.H	-6.307245	0.064927	-4.721483
71.H	-3.891563	0.675235	-4.894805
72.H	-1.106163	0.990975	-4.371523
73.H	1.748984	-3.007062	-4.246826
74.H	-1.592162	-2.617154	-4.116970
75.H	3.365146	5.114973	-3.264427
76.H	7.169225	-0.708217	-3.113134
77.H	6.873903	-3.166304	-2.793226
78.H	5.243606	0.823824	-2.841180
79.H	-7.353556	-0.207769	-2.472035
80.H	-5.223914	-3.721747	-2.667614
81.H	-4.840948	-6.170584	-2.356302
82.H	-2.550312	1.016396	-2.830855
83.H	-1.944322	-1.603273	-2.709954
84.H	-0.804624	-2.956763	-2.563212
85.H	-3.159197	3.631487	-2.635367
86.H	4.626754	-4.073110	-2.195256
87.H	-0.090580	3.478341	-2.402612
88.H	-2.836188	5.210857	-1.887806
89.H	2.927198	3.246980	-1.673199
90.H	2.698924	-2.532050	-1.926790
91.H	1.168272	-4.047500	-1.649317
92.H	0.023862	4.936943	-1.396528
93.H	-3.969116	-2.082885	-1.284625
94.H	-4.110628	4.169727	-1.237099
95.H	-3.214070	-6.956118	-0.633271
96.H	-0.085320	-5.163600	-1.087813
97.H	-6.003573	0.113625	-0.420262
98.H	0.739124	3.414673	-0.836442
99.H	1.625469	-5.499395	-0.730505
100.H	-5.002163	2.993526	0.105319
101.H	4.524781	-0.662526	0.182412
102.H	-1.994933	-5.321230	0.759110

103.H	2.787120	-2.272177	0.476853
104.H	-1.723372	5.433400	0.968271
105.H	5.193346	2.870563	0.984074
106.H	6.468914	-1.816692	1.210909
107.H	5.736384	5.279686	1.169725
108.H	3.219206	-3.833828	1.186803
109.H	-2.823947	4.194170	1.619302
110.H	-6.541883	3.807194	1.860642
111.H	3.933473	6.941799	1.637094
112.H	1.036820	3.749707	1.702690
113.H	-1.072865	4.008606	1.811144
114.H	1.574160	6.166998	1.890418
115.H	-0.592831	-5.452665	2.159218
116.H	-4.513573	-3.404290	2.001498
117.H	1.146175	-5.800307	2.123855
118.H	2.496485	-2.568791	2.199935
119.H	-3.774096	-0.250832	2.658788
120.H	0.508023	-4.532599	3.191656
121.H	-1.526461	0.221460	3.178818
122.H	7.168981	-1.250533	3.540245
123.H	-1.497780	1.990474	3.293292
124.H	-0.615454	-2.209884	3.392772
125.H	-6.719609	2.589821	4.033072
126.H	4.001209	1.656018	3.776402
127.H	0.872857	3.516755	4.212984
128.H	1.190975	-1.383973	4.059731
129.H	-5.333624	0.549930	4.423792
130.H	-5.208719	-3.854327	4.336357
131.H	2.454208	2.795266	4.547770
132.H	5.923048	0.499238	4.813413
133.H	-1.411278	0.999880	4.764765
134.H	2.554584	-0.432017	4.661480
135.H	1.089927	2.482172	5.639024
136.H	1.031765	-0.479113	5.583749
137.H	-1.304793	-2.657080	5.736101
138.H	-3.609955	-3.487599	6.217548
139.N	0.983373	-0.356358	-2.288937
140.N	-1.823442	1.694506	-0.332415
141.N	-0.350696	-2.384958	0.714462
142.N	1.234383	1.026310	1.931536
143.Si	0.272280	-1.027637	-3.754891
144.Si	-1.764045	3.442876	-0.541176
145.Si	0.763862	-3.740181	0.817283
146.Si	0.804051	1.066458	3.636839
147.P	2.433279	0.394902	-2.017236
148.P	-3.096119	0.690124	0.000852
149.P	-2.000905	-2.319525	0.826225
150.P	2.649532	1.360719	1.137792
151.Th	0.006288	0.011184	0.001011

Supplementary Table 12. Coordinates of model system 3Ce

Ce	0.000000	0.000000	0.000000
C	0.000000	0.000000	-2.391999
C	0.000000	0.000000	2.391999
N	2.231387	0.000000	-1.101606
N	-2.231387	0.000000	-1.101606
N	0.000000	2.231387	1.101606
N	0.000000	-2.231387	1.101606
P	0.000000	1.649344	2.612362
P	0.000000	-1.649344	2.612362
P	1.649344	0.000000	-2.612362
P	-1.649344	0.000000	-2.612362
Si	3.832432	0.000000	-0.462206
Si	-3.832432	0.000000	-0.462206
Si	0.000000	3.832432	0.462206
Si	0.000000	-3.832432	0.462206
H	2.164427	1.083812	-3.370234
H	-2.164427	-1.083812	-3.370234
H	2.164427	-1.083812	-3.370234
H	-2.164427	1.083812	-3.370234
H	1.083812	2.164427	3.370234
H	-1.083812	-2.164427	3.370234
H	1.083812	-2.164427	3.370234
H	-1.083812	2.164427	3.370234
H	4.062429	1.200410	0.388559
H	-4.062429	-1.200410	0.388559
H	4.062429	-1.200410	0.388559
H	-4.062429	1.200410	0.388559
H	4.855523	0.000000	-1.549646
H	-4.855523	0.000000	-1.549646
H	1.200410	4.062429	-0.388559
H	-1.200410	-4.062429	-0.388559
H	1.200410	-4.062429	-0.388559
H	-1.200410	4.062429	-0.388559
H	0.000000	4.855523	1.549646
H	0.000000	-4.855523	1.549646

Supplementary Table 13. Coordinates of model system 3U

U	0.000000	0.000000	0.000000
C	0.000000	0.000000	-2.410999
C	0.000000	0.000000	2.410999
N	2.199672	0.000000	-1.098839
N	-2.199672	0.000000	-1.098839
N	0.000000	2.199672	1.098839
N	0.000000	-2.199672	1.098839
P	0.000000	1.640727	2.627879
P	0.000000	-1.640727	2.627879
P	1.640727	0.000000	-2.627879
P	-1.640727	0.000000	-2.627879
Si	3.813569	0.000000	-0.464734
Si	-3.813569	0.000000	-0.464734
Si	0.000000	3.813569	0.464734
Si	0.000000	-3.813569	0.464734
H	2.179551	1.083811	-3.368523
H	-2.179551	-1.083811	-3.368523
H	2.179551	-1.083811	-3.368523
H	-2.179551	1.083811	-3.368523
H	1.083811	2.179551	3.368523
H	-1.083811	-2.179551	3.368523
H	1.083811	-2.179551	3.368523
H	-1.083811	2.179551	3.368523
H	4.048941	1.200963	0.383165
H	-4.048941	-1.200963	0.383165
H	4.048941	-1.200963	0.383165
H	-4.048941	1.200963	0.383165
H	4.825399	0.000000	-1.562390
H	-4.825399	0.000000	-1.562390
H	1.200963	4.048941	-0.383165
H	-1.200963	-4.048941	-0.383165
H	1.200963	-4.048941	-0.383165
H	-1.200963	4.048941	-0.383165
H	0.000000	4.825399	1.562390
H	0.000000	-4.825399	1.562390

Supplementary Table 14. Coordinates of model system 3Th

Th	0.000000	0.000000	0.000000
C	0.000000	0.000000	-2.514999
C	0.000000	0.000000	2.514999
N	2.198769	0.000000	-1.147172
N	-2.198769	0.000000	-1.147172
N	0.000000	2.198769	1.147172
N	0.000000	-2.198769	1.147172
P	0.000000	1.639151	2.685546
P	0.000000	-1.639151	2.685546
P	1.639151	0.000000	-2.685546
P	-1.639151	0.000000	-2.685546
Si	3.814971	0.000000	-0.513464
Si	-3.814971	0.000000	-0.513464
Si	0.000000	3.814971	0.513464
Si	0.000000	-3.814971	0.513464
H	2.209466	1.092048	-3.417209
H	-2.209466	-1.092048	-3.417209
H	2.209466	-1.092048	-3.417209
H	-2.209466	1.092048	-3.417209
H	1.092048	2.209466	3.417209
H	-1.092048	-2.209466	3.417209
H	1.092048	-2.209466	3.417209
H	-1.092048	2.209466	3.417209
H	4.049115	1.207837	0.342466
H	-4.049115	-1.207837	0.342466
H	4.049115	-1.207837	0.342466
H	-4.049115	1.207837	0.342466
H	4.837379	0.000000	-1.613001
H	-4.837379	0.000000	-1.613001
H	1.207837	4.049115	-0.342466
H	-1.207837	-4.049115	-0.342466
H	1.207837	-4.049115	-0.342466
H	-1.207837	4.049115	-0.342466
H	0.000000	4.837379	1.613001
H	0.000000	-4.837379	1.613001

Supplementary Table 15. QTAIM-calculated properties of truncated Ce(BIPM^{TMS})$_2$ complexes, derived from CASSCF- and RASSCF-calculated densities. *q*(M) = atomic charge, ρ_{BCP} = charge density at the bond critical point, ε_{BCP} = bond ellipticity at the bond critical point, δ(M,L) = delocalisation index. Values in parentheses are derived from RASSCF-calculated densities.

All values are in atomic units.

**Ion: State**	***q*(M)**	***q*(C)**	**M-C**			**M-N**		
			ρ_{BCP}	ε_{BCP}	δ(M,C)	ρ_{BCP}	ε_{BCP}	δ(M,N)
**Ce: $^1A_1$**	+2.65<br>(+2.62)	-2.57<br>(-2.60)	0.086<br>(0.086)	0.400<br>(0.408)	0.620<br>(0.612)	0.058<br>(0.059)	0.177<br>(0.186)	0.378<br>(0.375)
**Th: $^1A_1$**	+2.91<br>(+2.88)	-2.67<br>(-2.69)	0.071<br>(0.071)	0.424<br>(0.425)	0.474<br>(0.475)	0.065<br>(0.066)	0.207<br>(0.213)	0.414<br>(0.409)
**U: $^3B_1/^3B_2$**	+2.77<br>(+2.72)	-2.63<br>(-2.64)	0.087<br>(0.084)	0.374<br>(0.379)	0.567<br>(0.563)	0.066<br>(0.068)	0.237<br>(0.234)	0.406<br>(0.415)
**U: $^3A_1$**	-<br>(+2.73)	-<br>(-2.64)	-<br>(0.083)	-<br>(0.423)	-<br>(0.557)	-<br>(0.066)	-<br>(0.113)	-<br>(0.416)
**U: $^3A_2$**	-<br>(+2.73)	-<br>(-2.64)	-<br>(0.084)	-<br>(0.398)	-<br>(0.565)	-<br>(0.067)	-<br>(0.150)	-<br>(0.413)

Supplementary Table 16. Final coordinates and energy for the single point energy calculation on the final geometry optimised coordinates of 3Pr with 5p core

1.C	2.751768	2.193714	-5.984841
2.C	-2.786356	-2.750317	-5.633817
3.C	3.189572	3.381200	-5.386286
4.C	2.280279	1.141513	-5.195019
5.C	-3.573582	3.353468	-5.117083
6.C	2.937439	-3.876983	-5.108446
7.C	-2.918124	-3.962564	-4.947110
8.C	-2.186829	3.238800	-4.985351
9.C	-2.479432	-1.577578	-4.937907
10.C	1.582410	-3.643851	-4.862358
11.C	3.893213	-2.954309	-4.662360
12.C	-4.410298	2.404320	-4.515440
13.C	-1.636245	2.189497	-4.240055
14.C	3.144649	3.514060	-3.993951
15.C	1.183682	-2.502619	-4.157667
16.C	3.491182	-1.811831	-3.967932
17.C	-3.858248	1.354771	-3.779047
18.C	2.245215	1.261549	-3.794163
19.C	2.131073	-1.581946	-3.694730
20.C	-2.744832	-3.996126	-3.558555
21.C	-2.465685	1.245672	-3.620813
22.C	-2.300009	-1.603376	-3.543760
23.C	2.675181	2.460354	-3.206071
24.C	-2.438169	-2.824093	-2.865251
25.C	-0.065783	-0.020262	-2.474133
26.C	5.186058	0.728762	-1.608023
27.C	-0.283464	3.836947	-1.508014
28.C	-5.093521	-1.317831	-1.245371
29.C	0.627343	-3.945645	-0.992419
30.C	4.397571	-1.996904	-0.478679
31.C	-4.680838	1.577906	-0.356439
32.C	2.191555	4.371376	0.224162
33.C	-1.898138	-4.417262	0.668221
34.C	-0.598331	5.327949	1.082757
35.C	3.873930	0.579794	1.111744
36.C	-3.752084	-0.723937	1.404945
37.C	0.874971	-5.091557	1.787690
38.C	0.064848	0.161772	2.434851
39.C	-2.578832	2.767117	2.601359
40.C	2.717216	-2.370911	3.005633
41.C	-3.771808	3.175177	3.200993
42.C	-1.437490	2.506159	3.374946
43.C	3.884373	-2.716003	3.689295
44.C	1.413422	2.499970	3.607909
45.C	1.544221	-2.043563	3.703017
46.C	1.566260	3.879182	3.832616
47.C	-1.305056	-2.008570	3.876206
48.C	-1.562925	-3.363288	4.145590
49.C	2.288054	1.609670	4.244746
50.C	-3.838820	3.327283	4.590529

51.C	-2.090243	-1.033121	4.503997
52.C	2.581151	4.356561	4.664352
53.C	-1.511413	2.665872	4.769751
54.C	-2.596390	-3.732602	5.009339
55.C	3.893586	-2.740176	5.088609
56.C	1.562222	-2.069489	5.108725
57.C	3.298686	2.084398	5.087774
58.C	-2.705717	3.072738	5.372116
59.C	3.450225	3.457860	5.296669
60.C	-3.117921	-1.399113	5.379458
61.C	-3.377318	-2.749175	5.629939
62.C	2.729813	-2.416212	5.795334
63.H	2.789481	2.079528	-7.069230
64.H	-2.925401	-2.714988	-6.715268
65.H	3.570554	4.197184	-6.002614
66.H	-4.004306	4.176602	-5.690021
67.H	3.250979	-4.772180	-5.648571
68.H	-3.158864	-4.877290	-5.491397
69.H	1.953564	0.215270	-5.670092
70.H	-1.527874	3.969236	-5.457241
71.H	-2.378833	-0.637761	-5.482386
72.H	4.952773	-3.125216	-4.858896
73.H	0.828066	-4.350820	-5.210283
74.H	-5.493555	2.481740	-4.621160
75.H	-0.554828	2.100045	-4.131814
76.H	0.126683	-2.324979	-3.963011
77.H	4.242589	-1.093286	-3.643330
78.H	3.485036	4.435439	-3.519355
79.H	-4.518208	0.613724	-3.330220
80.H	-2.850815	-4.935778	-3.014799
81.H	5.242899	0.438946	-2.666977
82.H	-5.210275	-1.140152	-2.324354
83.H	2.651766	2.557104	-2.122371
84.H	-0.060126	4.778014	-2.035530
85.H	0.171789	3.018820	-2.083302
86.H	-2.303060	-2.847445	-1.785809
87.H	5.036635	1.817808	-1.578779
88.H	0.112251	-3.282569	-1.701715
89.H	4.478739	-2.474772	-1.465815
90.H	-1.373370	3.690774	-1.535559
91.H	0.539688	-4.972032	-1.383254
92.H	6.169437	0.518505	-1.155407
93.H	-4.986249	1.922540	-1.354216
94.H	-4.791282	-2.368324	-1.122638
95.H	-6.087140	-1.204550	-0.780905
96.H	1.693357	-3.674881	-1.007468
97.H	2.341796	5.308831	-0.335335
98.H	5.381047	-2.066730	0.013637
99.H	2.789083	3.588156	-0.264212
100.H	-3.998736	2.336139	0.052285
101.H	-1.962643	-5.397181	0.167997
102.H	3.684306	-2.592696	0.108172

103.H	-5.578561	1.553924	0.282845
104.H	-2.526582	-3.710911	0.107390
105.H	-0.417898	6.247234	0.500829
106.H	2.603658	4.509631	1.234649
107.H	-1.682038	5.140432	1.076712
108.H	3.685942	1.662736	1.071273
109.H	0.698955	-6.099730	1.376822
110.H	-2.346893	-4.519454	1.666947
111.H	-3.291858	-1.719833	1.481330
112.H	4.852213	0.427938	1.594816
113.H	-2.517723	2.649485	1.520732
114.H	1.956894	-4.899955	1.739526
115.H	3.111877	0.141488	1.773000
116.H	-4.754697	-0.782849	1.858149
117.H	-0.310570	5.541298	2.121618
118.H	-3.155816	-0.034757	2.020915
119.H	2.704193	-2.358961	1.917668
120.H	-4.648670	3.374084	2.583092
121.H	0.601822	-5.118898	2.852189
122.H	4.785024	-2.971634	3.129515
123.H	0.884001	4.586851	3.365094
124.H	-0.953322	-4.138142	3.684830
125.H	2.170603	0.538150	4.081840
126.H	-1.897017	0.019591	4.298643
127.H	2.689827	5.430290	4.825360
128.H	-4.769794	3.646347	5.062100
129.H	-2.790910	-4.788992	5.200974
130.H	-0.632947	2.473753	5.387750
131.H	4.802635	-3.016403	5.625725
132.H	3.965087	1.374698	5.579898
133.H	0.659626	-1.824169	5.670332
134.H	-3.717975	-0.624187	5.858949
135.H	4.239099	3.828846	5.953627
136.H	-4.183953	-3.036788	6.306667
137.H	-2.749665	3.195180	6.455288
138.H	2.726771	-2.439124	6.886189
139.Pr	0.021939	0.014767	-0.014539
140.N	2.227573	-0.021359	-1.230102
141.N	-2.268161	-0.144908	-1.067016
142.N	0.095394	2.332132	0.980999
143.N	0.043779	-2.175900	1.272364
144.P	1.591777	-0.101779	-2.737733
145.P	-1.736766	-0.113383	-2.615201
146.P	0.065399	1.838207	2.540807
147.P	0.061830	-1.486880	2.755345
148.Si	3.857549	-0.183562	-0.607596
149.Si	-3.883053	-0.144299	-0.375396
150.Si	0.353103	3.908370	0.258054
151.Si	-0.091967	-3.838702	0.741009

Supplementary Table 17. Final coordinates and energy for the single point energy calculation on the final geometry optimised coordinates of 3Tb with 5p core

1.C	2.721137	2.181741	-5.956937
2.C	-2.778498	-2.731292	-5.611853
3.C	3.161101	3.374046	-5.369162
4.C	2.265266	1.130790	-5.156837
5.C	-3.559250	3.343485	-5.106384
6.C	2.943273	-3.858599	-5.100111
7.C	-2.912050	-3.948212	-4.933644
8.C	-2.173418	3.224965	-4.968693
9.C	-2.475288	-1.562851	-4.907179
10.C	1.589049	-3.628683	-4.846734
11.C	3.899706	-2.941542	-4.644775
12.C	-4.401191	2.402217	-4.499805
13.C	-1.630067	2.180972	-4.211039
14.C	3.131759	3.512194	-3.977216
15.C	1.192472	-2.496549	-4.126836
16.C	3.499425	-1.808984	-3.933032
17.C	-3.855709	1.358385	-3.750171
18.C	2.245922	1.256129	-3.755485
19.C	2.140533	-1.582449	-3.652058
20.C	-2.742782	-3.990486	-3.545034
21.C	-2.464464	1.247088	-3.582948
22.C	-2.301351	-1.597093	-3.511805
23.C	2.675584	2.459901	-3.178991
24.C	-2.439188	-2.822678	-2.842921
25.C	-0.064093	-0.022609	-2.451909
26.C	5.162363	0.728881	-1.589826
27.C	-0.281761	3.846757	-1.542770
28.C	-5.072587	-1.304001	-1.233344
29.C	0.628948	-3.962651	-1.023894
30.C	4.387004	-1.990960	-0.456638
31.C	-4.658701	1.583664	-0.341409
32.C	2.188789	4.352859	0.212063
33.C	-1.899871	-4.402843	0.654190
34.C	-0.599019	5.299075	1.065248
35.C	3.890863	0.590722	1.146021
36.C	-3.767199	-0.727092	1.435329
37.C	0.871297	-5.068754	1.769042
38.C	0.060077	0.158905	2.413585
39.C	-2.579940	2.767272	2.581251
40.C	2.714290	-2.370781	2.981345
41.C	-3.767150	3.176107	3.192408
42.C	-1.431686	2.506375	3.343388
43.C	3.878819	-2.710990	3.672458
44.C	1.414121	2.500251	3.565668
45.C	1.536237	-2.046488	3.670524
46.C	1.561137	3.877796	3.804332
47.C	-1.309968	-2.018427	3.837193
48.C	-1.565765	-3.371494	4.117635
49.C	2.288376	1.608414	4.201459
50.C	-3.821117	3.328748	4.582307

51.C	-2.087957	-1.040133	4.470250
52.C	2.570582	4.351943	4.644737
53.C	-1.493368	2.664565	4.739622
54.C	-2.588954	-3.735764	4.995885
55.C	3.880608	-2.731294	5.071865
56.C	1.547544	-2.066124	5.077037
57.C	3.294561	2.079563	5.051724
58.C	-2.680874	3.073025	5.353325
59.C	3.441020	3.451832	5.272445
60.C	-3.108017	-1.401083	5.356723
61.C	-3.364968	-2.749611	5.617748
62.C	2.712066	-2.407317	5.771006
63.H	2.743726	2.063958	-7.041526
64.H	-2.912401	-2.689410	-6.693840
65.H	3.530134	4.189409	-5.993905
66.H	-3.985015	4.162303	-5.689683
67.H	3.255227	-4.746135	-5.653697
68.H	-3.150081	-4.859585	-5.484904
69.H	1.935536	0.201909	-5.624335
70.H	-1.509720	3.947518	-5.445772
71.H	-2.372001	-0.619865	-5.445952
72.H	4.958560	-3.108461	-4.847784
73.H	0.833579	-4.330385	-5.202705
74.H	-5.483718	2.480760	-4.612519
75.H	-0.549481	2.086031	-4.101120
76.H	0.135702	-2.318941	-3.931157
77.H	4.251957	-1.094587	-3.603244
78.H	3.471967	4.437563	-3.510539
79.H	-4.520530	0.622851	-3.300115
80.H	-2.848288	-4.933922	-3.007819
81.H	5.208989	0.436367	-2.648352
82.H	-5.177772	-1.123607	-2.313019
83.H	2.659005	2.563650	-2.096264
84.H	-0.057117	4.797603	-2.051955
85.H	0.174626	3.038021	-2.128836
86.H	-2.303988	-2.853262	-1.764081
87.H	5.020803	1.818865	-1.562591
88.H	0.119408	-3.306812	-1.742655
89.H	4.470968	-2.456402	-1.449540
90.H	-1.371581	3.701001	-1.572833
91.H	0.537782	-4.995028	-1.398111
92.H	6.148630	0.513937	-1.145628
93.H	-4.952365	1.922358	-1.344525
94.H	-4.781922	-2.357738	-1.110254
95.H	-6.070026	-1.182793	-0.778796
96.H	1.696127	-3.696048	-1.038078
97.H	2.340745	5.296329	-0.336744
98.H	5.370720	-2.062611	0.034920
99.H	2.795216	3.576630	-0.275958
100.H	-3.981573	2.344335	0.069863
101.H	-1.970036	-5.386936	0.163019
102.H	3.675815	-2.597660	0.121050

103.H	-5.563910	1.563204	0.287259
104.H	-2.535991	-3.701090	0.096795
105.H	-0.407838	6.223796	0.495362
106.H	2.589227	4.483727	1.228233
107.H	-1.684069	5.120803	1.050107
108.H	3.698488	1.672917	1.105224
109.H	0.683766	-6.081064	1.373487
110.H	-2.336189	-4.497513	1.659120
111.H	-3.312133	-1.725132	1.513664
112.H	4.879644	0.442461	1.608679
113.H	-2.528843	2.646643	1.500844
114.H	1.954788	-4.888908	1.712495
115.H	3.142130	0.151566	1.820348
116.H	-4.777982	-0.783422	1.870457
117.H	-0.317244	5.499761	2.108098
118.H	-3.176978	-0.043102	2.061691
119.H	2.707660	-2.358098	1.893771
120.H	-4.649987	3.374754	2.583078
121.H	0.605190	-5.079543	2.835527
122.H	4.782909	-2.965634	3.117819
123.H	0.877077	4.587427	3.343135
124.H	-0.960018	-4.149764	3.657748
125.H	2.171992	0.537569	4.033613
126.H	-1.895069	0.011789	4.260410
127.H	2.673422	5.424459	4.816276
128.H	-4.747429	3.648417	5.062595
129.H	-2.778769	-4.790791	5.198850
130.H	-0.610175	2.469468	5.349904
131.H	4.786879	-3.004050	5.615714
132.H	3.960629	1.368156	5.541516
133.H	0.642578	-1.819032	5.633733
134.H	-3.703355	-0.623221	5.837140
135.H	4.226240	3.820465	5.935096
136.H	-4.164497	-3.033925	6.304538
137.H	-2.714259	3.194140	6.437319
138.H	2.703539	-2.426234	6.862028
139.Tb	0.019154	0.013953	-0.014456
140.N	2.199544	-0.020258	-1.171748
141.N	-2.236357	-0.144632	-1.016402
142.N	0.091249	2.296746	0.929424
143.N	0.044760	-2.151106	1.217296
144.P	1.592462	-0.103995	-2.693318
145.P	-1.732000	-0.112148	-2.576626
146.P	0.063938	1.831955	2.500962
147.P	0.057284	-1.489205	2.715520
148.Si	3.842403	-0.176747	-0.569545
149.Si	-3.864368	-0.140981	-0.343578
150.Si	0.350563	3.885959	0.224158
151.Si	-0.091812	-3.825100	0.705358

Supplementary Table 18. Final coordinates and energy for the single point energy calculation on the final geometry optimised coordinates of 3Pr with 5p valence

1.C	2.752517	2.192792	-5.981837
2.C	-2.785653	-2.749485	-5.628428
3.C	3.186178	3.382141	-5.384244
4.C	2.282273	1.140364	-5.191545
5.C	-3.573764	3.353882	-5.114047
6.C	2.934614	-3.877629	-5.103601
7.C	-2.915056	-3.961852	-4.941800
8.C	-2.186974	3.239329	-4.983193
9.C	-2.479562	-1.576205	-4.932803
10.C	1.579558	-3.643040	-4.858850
11.C	3.890702	-2.955542	-4.656813
12.C	-4.410003	2.404693	-4.511762
13.C	-1.635876	2.190439	-4.237527
14.C	3.139104	3.516339	-3.992142
15.C	1.181144	-2.501404	-4.154394
16.C	3.488842	-1.812678	-3.962721
17.C	-3.857459	1.355508	-3.775271
18.C	2.244686	1.261708	-3.790805
19.C	2.128673	-1.581469	-3.690370
20.C	-2.740236	-3.995166	-3.553512
21.C	-2.464794	1.246850	-3.617027
22.C	-2.298249	-1.601898	-3.538794
23.C	2.671601	2.462173	-3.203678
24.C	-2.434161	-2.822949	-2.860386
25.C	-0.065386	-0.017096	-2.459933
26.C	5.184551	0.727720	-1.602354
27.C	-0.284951	3.839090	-1.511497
28.C	-5.090941	-1.317850	-1.242912
29.C	0.630958	-3.942295	-0.995985
30.C	4.394667	-1.998442	-0.476746
31.C	-4.680785	1.576559	-0.351095
32.C	2.190541	4.372972	0.220201
33.C	-1.893476	-4.420510	0.665388
34.C	-0.599155	5.324418	1.082259
35.C	3.871362	0.576508	1.116268
36.C	-3.750118	-0.726376	1.407819
37.C	0.880520	-5.088462	1.783961
38.C	0.064635	0.160853	2.418161
39.C	-2.578958	2.760462	2.597312
40.C	2.716898	-2.367941	3.004020
41.C	-3.772086	3.166371	3.197989
42.C	-1.435661	2.504345	3.369544
43.C	3.883255	-2.713466	3.688906
44.C	1.414584	2.497048	3.602471
45.C	1.542383	-2.042431	3.699592
46.C	1.567184	3.876055	3.828989
47.C	-1.306439	-2.005433	3.870598
48.C	-1.565209	-3.359821	4.140826
49.C	2.288242	1.606290	4.240199
50.C	-3.837187	3.322127	4.587195

51.C	-2.089668	-1.029039	4.499540
52.C	2.580639	4.352722	4.662818
53.C	-1.507933	2.666955	4.764200
54.C	-2.597134	-3.727950	5.006796
55.C	3.890033	-2.740551	5.088062
56.C	1.558337	-2.070249	5.105394
57.C	3.297411	2.080345	5.085596
58.C	-2.702238	3.072721	5.367577
59.C	3.448579	3.453615	5.296004
60.C	-3.116753	-1.393900	5.376242
61.C	-3.377110	-2.743730	5.627228
62.C	2.724814	-2.418390	5.793265
63.H	2.792549	2.077323	-7.066094
64.H	-2.925862	-2.714456	-6.709818
65.H	3.565677	4.198520	-6.001108
66.H	-4.005061	4.176874	-5.686777
67.H	3.247932	-4.773276	-5.643081
68.H	-3.155126	-4.876829	-5.485883
69.H	1.958732	0.213001	-5.666433
70.H	-1.528354	3.969452	-5.455873
71.H	-2.381095	-0.636164	-5.477705
72.H	4.950224	-3.127248	-4.852250
73.H	0.825047	-4.349285	-5.207304
74.H	-5.493308	2.481570	-4.617107
75.H	-0.554304	2.101686	-4.130119
76.H	0.123925	-2.323747	-3.960933
77.H	4.240825	-1.095208	-3.637407
78.H	3.476856	4.438973	-3.518146
79.H	-4.517406	0.614618	-3.326236
80.H	-2.844109	-4.935072	-3.009519
81.H	5.242148	0.438653	-2.661199
82.H	-5.208234	-1.138754	-2.321477
83.H	2.647297	2.559959	-2.120175
84.H	-0.064427	4.781782	-2.037090
85.H	0.172176	3.023118	-2.088116
86.H	-2.297746	-2.846346	-1.781148
87.H	5.035669	1.816477	-1.571787
88.H	0.114557	-3.278830	-1.703857
89.H	4.477624	-2.474972	-1.464585
90.H	-1.374380	3.689739	-1.538046
91.H	0.546082	-4.968094	-1.388617
92.H	6.166772	0.515883	-1.148264
93.H	-4.991069	1.919662	-1.348020
94.H	-4.788192	-2.368198	-1.121575
95.H	-6.084291	-1.205554	-0.777524
96.H	1.696122	-3.668251	-1.009813
97.H	2.339038	5.310871	-0.338926
98.H	5.377533	-2.069626	0.017036
99.H	2.789669	3.591131	-0.268149
100.H	-3.998551	2.336931	0.053665
101.H	-1.955893	-5.400387	0.164861
102.H	3.680603	-2.596139	0.107763

103.H	-5.575924	1.552035	0.291876
104.H	-2.524811	-3.715706	0.105928
105.H	-0.420042	6.244621	0.501356
106.H	2.602279	4.511838	1.230727
107.H	-1.682810	5.136298	1.077539
108.H	3.687775	1.660107	1.075994
109.H	0.706519	-6.096668	1.372359
110.H	-2.340712	-4.524535	1.664630
111.H	-3.289253	-1.722016	1.482124
112.H	4.847448	0.419994	1.602349
113.H	-2.519301	2.640257	1.516921
114.H	1.962084	-4.894788	1.736396
115.H	3.105097	0.140727	1.774455
116.H	-4.752269	-0.786189	1.861733
117.H	-0.310152	5.536826	2.121056
118.H	-3.153200	-0.037804	2.023811
119.H	2.705789	-2.354105	1.916092
120.H	-4.650814	3.360327	2.581272
121.H	0.606760	-5.116755	2.848309
122.H	4.785071	-2.967039	3.130073
123.H	0.885959	4.584424	3.361071
124.H	-0.956069	-4.135492	3.680399
125.H	2.171847	0.534855	4.076521
126.H	-1.895910	0.023619	4.294742
127.H	2.689375	5.426350	4.824568
128.H	-4.768588	3.639124	5.059389
129.H	-2.791414	-4.783995	5.199835
130.H	-0.628332	2.477922	5.381500
131.H	4.798210	-3.017240	5.626447
132.H	3.963185	1.370267	5.578045
133.H	0.655241	-1.825154	5.666228
134.H	-3.715873	-0.618676	5.855888
135.H	4.236676	3.824300	5.954097
136.H	-4.183886	-3.030453	6.304153
137.H	-2.744734	3.197999	6.450748
138.H	2.720434	-2.443082	6.883920
139.Pr	0.022140	0.015250	-0.015535
140.N	2.224060	-0.021770	-1.222883
141.N	-2.264647	-0.142808	-1.059776
142.N	0.096566	2.328339	0.972029
143.N	0.043607	-2.173442	1.264628
144.P	1.591702	-0.101117	-2.731870
145.P	-1.736480	-0.111472	-2.608896
146.P	0.066749	1.837609	2.532726
147.P	0.061022	-1.486972	2.748240
148.Si	3.854833	-0.185885	-0.602562
149.Si	-3.880912	-0.144052	-0.371117
150.Si	0.352459	3.907110	0.253897
151.Si	-0.088731	-3.837390	0.736822

Supplementary Table 19. Final coordinates and energy for the single point energy calculation on the final geometry optimised coordinates of 3Tb with 5p valence

1.C	2.694907	2.189320	-5.949990
2.C	-2.784967	-2.730594	-5.606549
3.C	3.138786	3.380917	-5.363460
4.C	2.249034	1.135486	-5.148122
5.C	-3.551891	3.325198	-5.124070
6.C	2.958654	-3.839452	-5.113124
7.C	-2.925752	-3.947411	-4.929667
8.C	-2.166470	3.209043	-4.979511
9.C	-2.474258	-1.564969	-4.900701
10.C	1.603259	-3.613677	-4.861717
11.C	3.912498	-2.926712	-4.643908
12.C	-4.395400	2.387649	-4.514033
13.C	-1.625254	2.170718	-4.212673
14.C	3.124099	3.515270	-3.970818
15.C	1.202461	-2.489342	-4.131458
16.C	3.508399	-1.801778	-3.922255
17.C	-3.851881	1.349259	-3.755235
18.C	2.242404	1.258024	-3.746263
19.C	2.148177	-1.578787	-3.644458
20.C	-2.755574	-3.992171	-3.541188
21.C	-2.461235	1.240227	-3.581827
22.C	-2.300821	-1.601251	-3.505252
23.C	2.676859	2.460491	-3.170895
24.C	-2.445140	-2.826717	-2.837826
25.C	-0.062191	-0.027524	-2.443189
26.C	5.161957	0.729811	-1.577912
27.C	-0.272459	3.846405	-1.550871
28.C	-5.072535	-1.297437	-1.227085
29.C	0.619755	-3.970610	-1.032800
30.C	4.381344	-1.992929	-0.446955
31.C	-4.649378	1.594056	-0.346532
32.C	2.188057	4.347229	0.218707
33.C	-1.904656	-4.397178	0.655147
34.C	-0.606224	5.294183	1.056515
35.C	3.895739	0.586639	1.159069
36.C	-3.774573	-0.714470	1.441732
37.C	0.869041	-5.068026	1.761300
38.C	0.059090	0.156880	2.408628
39.C	-2.578914	2.773772	2.586107
40.C	2.711829	-2.379388	2.978962
41.C	-3.761316	3.190074	3.201668
42.C	-1.429175	2.507476	3.343646
43.C	3.875013	-2.718835	3.672805
44.C	1.415138	2.500253	3.557291
45.C	1.533602	-2.050925	3.665500
46.C	1.556390	3.877813	3.798986
47.C	-1.311467	-2.022295	3.829498
48.C	-1.565788	-3.375465	4.110649
49.C	2.295920	1.610950	4.187552
50.C	-3.808658	3.344652	4.591557

51.C	-2.089128	-1.044799	4.464305
52.C	2.566251	4.354549	4.637403
53.C	-1.484149	2.667156	4.740100
54.C	-2.586547	-3.740629	4.991455
55.C	3.875082	-2.734288	5.072181
56.C	1.542855	-2.066438	5.072229
57.C	3.303145	2.084469	5.035230
58.C	-2.666920	3.082508	5.358369
59.C	3.443416	3.456952	5.259573
60.C	-3.106191	-1.406430	5.353768
61.C	-3.361535	-2.755120	5.615744
62.C	2.705873	-2.407129	5.768765
63.H	2.707232	2.073882	-7.035035
64.H	-2.919805	-2.686107	-6.688324
65.H	3.499728	4.198611	-5.990009
66.H	-3.975882	4.139530	-5.714842
67.H	3.273347	-4.720122	-5.676284
68.H	-3.170298	-4.856616	-5.481751
69.H	1.916866	0.206822	-5.614827
70.H	-1.501229	3.928593	-5.459209
71.H	-2.365665	-0.622255	-5.438752
72.H	4.972383	-3.090187	-4.844590
73.H	0.849779	-4.312185	-5.227998
74.H	-5.477595	2.464340	-4.631481
75.H	-0.544998	2.077289	-4.098664
76.H	0.144597	-2.315027	-3.937846
77.H	4.259271	-1.090343	-3.582429
78.H	3.467718	4.439792	-3.504811
79.H	-4.517850	0.615475	-3.303931
80.H	-2.866762	-4.935481	-3.005222
81.H	5.207261	0.443326	-2.638117
82.H	-5.175924	-1.121760	-2.307641
83.H	2.670212	2.561176	-2.087639
84.H	-0.042473	4.798059	-2.056255
85.H	0.186408	3.038389	-2.135855
86.H	-2.310934	-2.859106	-1.759021
87.H	5.021240	1.819776	-1.544565
88.H	0.108659	-3.318111	-1.753423
89.H	4.479680	-2.453851	-1.440610
90.H	-1.362318	3.702982	-1.588246
91.H	0.525695	-5.005163	-1.400400
92.H	6.148689	0.512267	-1.136062
93.H	-4.934355	1.932157	-1.352408
94.H	-4.784524	-2.351251	-1.098689
95.H	-6.070171	-1.171907	-0.774294
96.H	1.687298	-3.705940	-1.051657
97.H	2.344963	5.290638	-0.328832
98.H	5.357258	-2.069336	0.059242
99.H	2.795869	3.570036	-0.266097
100.H	-3.970873	2.352128	0.067460
101.H	-1.980256	-5.381880	0.166060
102.H	3.659641	-2.600889	0.116031

103.H	-5.558816	1.580060	0.276233
104.H	-2.540518	-3.694078	0.099114
105.H	-0.406678	6.221046	0.492952
106.H	2.583231	4.476897	1.237067
107.H	-1.691367	5.117887	1.027338
108.H	3.697213	1.667829	1.122264
109.H	0.673049	-6.081493	1.372894
110.H	-2.337439	-4.487990	1.661989
111.H	-3.324769	-1.714411	1.525483
112.H	4.888633	0.443038	1.614332
113.H	-2.532721	2.652371	1.505619
114.H	1.952998	-4.894209	1.694637
115.H	3.154452	0.141048	1.836981
116.H	-4.787594	-0.764488	1.872509
117.H	-0.336779	5.489310	2.103729
118.H	-3.183947	-0.030944	2.068076
119.H	2.706481	-2.370436	1.891370
120.H	-4.645203	3.394258	2.595725
121.H	0.611993	-5.071366	2.830028
122.H	4.779628	-2.976555	3.120263
123.H	0.867208	4.585502	3.342216
124.H	-0.960517	-4.153390	3.649734
125.H	2.183531	0.539985	4.017778
126.H	-1.897384	0.007310	4.254339
127.H	2.663664	5.427174	4.811619
128.H	-4.730587	3.671494	5.075187
129.H	-2.774861	-4.795943	5.194448
130.H	-0.599374	2.468728	5.346919
131.H	4.780415	-3.005877	5.618213
132.H	3.974852	1.374880	5.519974
133.H	0.637273	-1.816287	5.626695
134.H	-3.700497	-0.628836	5.835982
135.H	4.228846	3.827718	5.920857
136.H	-4.158816	-3.039700	6.305100
137.H	-2.695004	3.206273	6.441941
138.H	2.695824	-2.422369	6.859823
139.Tb	0.018771	0.013017	-0.013021
140.N	2.198161	-0.018243	-1.159823
141.N	-2.232851	-0.145776	-1.009081
142.N	0.086579	2.291968	0.923464
143.N	0.046836	-2.149393	1.207878
144.P	1.595135	-0.104338	-2.682714
145.P	-1.730440	-0.116483	-2.569859
146.P	0.062704	1.830295	2.495992
147.P	0.056524	-1.492049	2.708507
148.Si	3.841651	-0.177151	-0.558152
149.Si	-3.862715	-0.134162	-0.339621
150.Si	0.349217	3.882028	0.219989
151.Si	-0.095008	-3.824461	0.698169

Supplementary Methods

General

All manipulations were carried out using Schlenk techniques or an MBraun UniLab glovebox, under an atmosphere of dry N_2. Solvents were dried variously by passage through activated alumina towers, or distillation from alkali metals, and degassed before use. Solvents were stored over K-mirrors (except for THF which was stored over activated 4 Å molecular sieves). Deuterated solvents were distilled from K, degassed by three freeze-pump-thaw cycles and stored under dinitrogen. Benzophenone and 9-anthracene carboxaldehyde were dried under vacuum for 4 hours prior to use. Benzaldehyde was dried over activated 4Å molecular sieves prior to use. 18C6 was dissolved in THF, stored over activated 4Å sieves overnight, then decanted and THF removed under reduced pressure. The compounds [Ag(BPh_4)],[1] and [M($BIPM^{TMS}$)($BIPM^{TMS}$H)] (M = Ce, **1Ce**; M = Pr, **1Pr**),[2] [Tb(CH_2Ph)$_3$(THF)$_3$],[2] and [U($BIPM^{TMS}$)(Cl)$_3$(Li)(THF)$_2$][3] were prepared using published procedures. The synthesis of [U($BIPM^{TMS}$)(CH_2SiMe_3)$_2$] has been reported elsewhere without a crystal structure,[4] we report the structure here along with its synthesis for completeness.

^{1}H, ^{13}C, ^{29}Si and ^{31}P NMR spectra were recorded on a Bruker DPX400 or AV400 spectrometer operating at 400.2, 100.6, 79.5 and 162.0 MHz, respectively; chemical shifts are quoted in ppm and are relative to TMS (^{1}H, ^{13}C and ^{29}Si), and external 85% H_3PO_4/D_2O (^{31}P). FTIR spectra were recorded on a Bruker Tensor 27 spectrometer. Solution magnetic moments were recorded at 298 K using the Evans method.[5] Static variable-temperature magnetic moment data were recorded in an applied dc field of 0.1 T on a Quantum Design MPMS XL7 superconducting quantum interference device (SQUID) magnetometer using doubly recrystallised powdered samples. Care was taken to ensure complete thermalisation of the sample before each data point was measured and samples were immobilised in an eicosane matrix to prevent sample reorientation during measurements. Diamagnetic corrections were applied for using tabulated Pascal constants and measurements were corrected for the effect of the blank sample holders (flame sealed Wilmad NMR tube and straw) and

eicosane matrix. Low-temperature EPR spectra were measured at X- and Q-band microwave frequencies (ca. 9.4 and 34 GHz, respectively) on a Bruker EMX 300 EPR spectrometer equipped with a 1.8 T electromagnet. Polycrystalline samples were flame-sealed under vacuum in 1mm i.d. quartz tubing prior being used for Q-band EPR measurements. The same sealed samples were used for X-band measurements, with the only difference that an additional 3.5 mm i.d. quartz tube was used to support them inside the resonator cavity. UV/Vis/NIR spectra were recorded on a Perkin Elmer Lambda 750 spectrometer. Data were collected in 1mm path length cuvettes loaded in an MBraun UniLab glovebox and were run versus the appropriate reference solvent. Cyclic voltammetry experiments were performed on a Metrohm Autolab UK Ltd. PGSTAT20 potentiostat with a three-electrode arrangement in a single compartment cell under nitrogen. Pt wire working and secondary electrodes and a Ag/AgCl reference electrode. Sample solutions were 2mM in concentration of complex with 0.1 M dry $[N(Pr)_4][BAr^F{}_4]$ supporting electrolyte in dry THF, loaded into the cell in the glovebox. Cerium L_{III}-edge XANES measurements were performed using a Si(111) double-crystal monochromator on the Rossendorf Beamline at the European Synchrotron Radiation Facility (Grenoble, France). Higher harmonics were rejected by two Si coated mirrors. The spectra were collected using ionisation chambers filled with nitrogen and a 13-element Ge fluorescence detector. The samples were measured at 15 K in a closed-cycle He cryostat. The reference samples spectra of 0.01 M Ce(III) nitrate in H_2O and solid CeO_2 were measured at room temperature in transmission mode. Elemental microanalyses were carried out by Stephen Boyer at London Metropolitan University and Dr Tong Liu at The University of Nottingham.

Mechanistic Studies

Detailed mechanistic studies of the reactivity of **3Ce**, **3U**, and **3Th** were conducted with PhCHO. Since it is the least reactive, **3Ce** was found to be most amenable to a kinetic study and a full analysis was possible yielding data that can be fitted well to a second order reaction, assuming first order with respect to both **3Ce** and PCHO. These data yield: E^a = 40.034 ± 1.975 kJ mol^{-1}; $\Delta H^{\ddagger}$ =

37.228 ± 1.972 kJ mol^{-1}; $\Delta S^{\ddagger}$ = −194.423 ± 5.762 J mol^{-1} K^{-1}; $\Delta G^{\ddagger}$ (298K) = 95.166 ± 1.972 kJ mol^{-1}; *k* (298 K) = 1.28 $x10^{-4}$ ± 0.255 $x10^{-4}$ mol^{-1} dm^3 s^{-1}. For **3U**, the reaction is rapid (>80% of **3U** consumed within 15 minutes) and combined with the paramagnetic nature of **3U** accurate integration is difficult and has to be considered unreliable given the rapid consumption. For **3Th** the reaction is complete before any scans could be accumulated on the NMR spectrometer.

EPR Studies

The X-band EPR spectrum of **2Ce** at low temperature displays three major features, centred at effective g-values of g = 3.7, 1.0, and 0.8. These arise from the lowest Kramers doublet of the cerium(III) ion. In fact, each of these peaks is doubled which suggests two very slightly inequivalent molecules in the lattice at this temperature. Spectra at Q-band confirm these g-values, with the low-field doublet resolving as g_{1a} = 3.84 and g_{1b} = 3.54; the other g-values are beyond the magnetic field limit of the spectrometer at this frequency. The $^2F_{5/2}$ ground state of cerium(III) is split by the crystal field into three Kramer doublets. In a purely axial crystal field, these are described by $|m_J|$ = 1/2, 3/2 and 5/2, with characteristic effective g-values of: $g_{x,y}$ = 2.57, g_z = 0.85 ($|m_J|$ = 1/2), $g_{x,y}$ = 0, g_z = 2.57 ($|m_J|$ = 3/2), $g_{x,y}$ = 0, g_z = 4.29 ($|m_J|$ = 5/2). For **2Ce**, the observed sense of the g-anisotropy, and very large g_z, in the low temperature EPR spectra agree best with a $|m_J|$ = 1/2 ground state. This is consistent with a crystal field dominated by the negative charge concentrated on the C=Ce=C axis (with relatively short Ce-C distances) which should stabilise the $|m_J|$ = 5/2 doublet which has an oblate charge density distribution.[6] In contrast to **2Ce**, complex **3Ce** is EPR silent at both X- and Q-band frequencies, consistent with the compound being diamagnetic, as shown by SQUID magnetometry. In fact the vanishingly small measured χT value for **3Ce** at low temperature (0.003 cm^3Kcm^{-1}) represents just ca 0.6% of the calculated value for cerium(III) based on the effective g-values for **2Ce** [powder χT = 0.48 cm^3Kcm^{-1} estimated from $\mu_{eff}^2 = 1/4(g_1^2 + g_2^2 + g_3^2) = 7.997(\chi T)$].[7] Typical values for cerium(III) are above 0.4 cm^3Kcm^{-1}.[8] Therefore, **3Ce** is

clearly cerium(IV) with only very minor contamination by cerium(III) decomposition products, which are so small that they cannot even be detected by EPR which is a very sensitive technique.

General DFT and NBO Details for Full Models of 3Ce, 3U, 3Th, 3Pr, and 3Tb

Unrestricted or restricted geometry optimisations were performed for the full models of **3Ce**, **3U**, **3Th**, **3Pr**, and **3Tb** as appropriate using coordinates derived from the X-ray crystal structure. No constraints were imposed on the structures during the geometry optimisations. The calculations were performed using the Amsterdam Density Functional (ADF) suite version 2012.01.[9,10] The DFT geometry optimisations employed Slater type orbital (STO) triple-ζ-plus polarisation all-electron basis sets (from the ZORA/TZP database of the ADF suite). Scalar relativistic approaches were used within the ZORA Hamiltonian for the inclusion of relativistic effects and the local density approximation (LDA) with the correlation potential due to Vosko et al[11] was used in all of the calculations. Gradient corrections were performed using the functionals of Becke[12] and Perdew.[13] MOLEKEL[14] was used to prepare the three-dimensional plot of the electron density. Natural Bond Order (NBO) analyses were carried out with NBO 5.0.[15]

The NBO calculation localises the carbene lone pairs on the carbenes with no thorium contribution for **3Th**. This suggests that the thorium contribution is below the 5% default cut-off for the NBO program, but although well-suited to the treatment of covalent bonding interactions NBO tends to present a more ionic bonding picture.

Truncated Model Systems

Optimisation of the terminating hydrogen positions was performed at the density functional level of theory using version 6.4 of the TURBOMOLE software package,[16] employing the hybrid-GGA PBE0 functional,[17] which contains a Hartree-Fock contribution of 25% to the exchange energy. Ahlrichs basis sets[18] of polarised triple-zeta quality (def-TZVP for Ce, Th, U; def2-TZVP for all

other atoms) were used for partial DFT optimisations. CASSCF and RASSCF calculations on the truncated complexes employed version 7.6 of the MOLCAS software package.[19,20] Throughout, ANO-RCC basis sets[21-23] of approximate polarised triple-zeta quality were employed, with scalar relativistic effects incorporated via the 2nd order Douglas-Kroll-Hess Hamiltonian.[24,25] RASSCF calculations employed three active spaces. RAS1 contained only occupied orbitals from the monodeterminantal reference wavefunction, RAS2 contained both occupied and virtual orbitals, and RAS3 only virtual orbitals. Full configuration interaction (CI) was performed in RAS2, while truncated CI was performed between the RAS1, RAS2 and RAS3 subspaces. Orbital optimisation was performed in all subspaces. The choice of active subspaces ensured that all M=C and M-N interactions were accurately modelled by a set RASSCF(n,2,2;12,7,12) calculations. The number of correlated electrons, n, was 24 for the Ce and Th complexes and 26 for the U complex. Occupation numbers of formally unoccupied d-orbitals was effectively 0, indicating that the inclusion of these orbitals in the active subspaces is not required. In all cases, code restrictions required calculations to be performed in C_{2v} symmetry, leading to the possibility of a $^3A_1$, $^3A_2$ $^3B_1$ or $^3B_2$ ground state for the open-shell uranium complex. State-averaged RASSCF calculations (six state averages for all irreps excepting A_1, in which only three 5f^2 triplet configurations can be defined) indicated a degenerate $^3B_1$/$^3B_2$ ground state, corresponding to a state of E symmetry in the full idealised D_{2d} point group. This state lies a few hundredths of eV below the lowest energy $^3A_1$ and $^3A_2$ states. RASSCF-calculated natural orbital occupation numbers were used in order to identify appropriate complete active spaces. Topological and integrated atomic properties were obtained using the quantum theory of atoms in molecules[26] (QTAIM). All QTAIM calculations were performed using version 13.11.04 of the AIMAll software package.[27]

Supplementary References

1. Popovych, O. Conductometric determination of solubility and solubility products of silver salts in a medium of low dielectric constant. *Anal. Chem.* **38**, 117-119 (1966).
2. Wooles, A. J., Mills, D. P., Lewis, W., Blake, A. J. & Liddle, S. T. Lanthanide Tri-Benzyl Complexes: Structural Variations and Useful Precursors to Phosphorus-Stabilised Lanthanide Carbenes. *Dalton Trans.* **39**, 500-510 (2010).
3. Cooper, O. J. *et al*. Uranium-Carbon Multiple Bonding: Facile Access to the Pentavalent Uranium Carbene [U{C(PPh$_2$NSiMe$_3$)$_2$}(Cl)$_2$(I)] and Comparison of U^V=C and U^{IV}=C Double Bonds. *Angew. Chem. Int. Ed.* **50**, 2383-2386 (2011).
4. Lu, E. *et al*. Uranium-Carbene-Imido Metalla-Allenes: Ancillary-Ligand-Controlled *Cis*-/*Trans*-Isomerisation and Assessment of *Trans*-Influence in the $R_2C=U^{IV}=NR'$ Unit (R = Ph$_2$PNSiMe$_3$; R' = CPh$_3$). *Chem. Eur. J.* **22**, 11559-11563 (2016).
5. Evans, D. F. The determination of the paramagnetic susceptibility of substances in solution by nuclear magnetic resonance. *J. Chem. Soc.* 2003-2005 (1959).
6. Rinehart, J. D. & Long, J. R. Exploiting single-ion anisotropy in the design of f-element single-molecule magnets. *Chem. Sci.* **2**, 2078-2085 (2011).
7. Abraham, A. Bleaney, B. *Electron Paramagnetic Resonance of Transition Ions*: Clarendon Press: Oxford, 1970.
8. Walter, M. D. Booth, C. H. Lukens, W. W. & Andersen, R. A. Cerocene revisited: The electronic structure of and interconversion between $Ce_2(C_8H_8)_3$ and $Ce(C_8H_8)_2$. *Organometallics* **28**, 698-707 (2009).
9. Fonseca-Guerra, C., Snijders, J. G., Velde, G. Te & Baerends, E. J. Towards an order-N DFT method. *Theor. Chem. Acc.* **99**, 391-403 (1998).
10. Velde, G. te, Bickelhaupt, F. M., Van Gisbergen, S. J. A., Fonseca-Guerra, C., Baerends, E. J., Snijders, J. G., Ziegler, T. Chemistry with ADF. *J. Comput. Chem.* **22**, 931-967 (2001).

11. Vosko, S. H., Wilk, L. & Nusair, M. Accurate spin-dependent electron liquid correlation energies for local spin density calculations: A critical analysis. *Can. J. Phys.* **58**, 1200-1211 (1980).

12. Becke, A. D. Density-functional exchange-energy approximation with correct asymptotic behavior. *Phys. Rev. A* **38**, 3098-3100 (1988).

13. Perdew, J. P. Density-functional approximation for the correlation energy of the inhomogeneous electron gas. *Phys. Rev. B* **33**, 8822-8824 (1986).

14. Portmann, S. & Luthi, H. P. MOLEKEL: An interactive molecular graphics tool. *Chimia* **54**, 766-770 (2000).

15. NBO 5.0: Glendening, E. D., Badenhoop, J. K., Reed, A. E., Carpenter, J. E., Bohmann, J. A., Morales, C. M., Weinhold F. Theoretical Chemistry Institute, University of Wisconsin, Madison, WI, (2001). http://www.chem.wisc.edu/~nbo5.

16. TURBOMOLE V6.2, a development of University of Karlsruhe and Forschungszentrum Karlsruhe. Ahlrichs, R., Armbruster, M. K., Bär, M. & Baron, H.-P. (2010). http://www.turbomole.com

17. Adamo, C. & Barone, V. Toward reliable density functional methods without adjustable parameters: the PBE0 model. *J. Chem. Phys.* **110**, 6158-6170 (1999).

18. Weigend F. & Ahlrichs, R. Balanced basis sets of split valence, triple zeta valence and quadruple zeta valence quality for H to Rn: design and assessment of accuracy. *Phys. Chem. Chem. Phys.* **7**, 3297-3305 (2005).

19. Karlström, G. *et al.* MOLCAS: a program package for computational chemistry *Comput. Mater. Sci.* **28**, 222-239 (2003).

20. Aquilante, F. *et al.* MOLCAS 7: The Next Generation. *J. Comput. Chem.* **31**, 224-247 (2010).

21. Roos, B. O., Lindh, R., Malmqvist, P.-Å., Veryazov, V. & Widmark, P.-O. Main group atoms and dimers studied with a new relativistic ANO basis set. *J. Phys. Chem. A* **108**, 2851-2858 (2004).

22. Roos, B. O., Lindh, R., Malmqvist, P.-Å, Veryazov, V., Widmark, P.-O. & Borin, A. C. New relativistic atomic natural orbital basis sets for lanthanide atoms with applications to the Ce diatom and LuF_3. *J. Phys. Chem. A* **112**, 11431-11435 (2008).

23. Roos, B. O., Lindh, R., Malmqvist, P.-Å., Veryazov, V. & Widmark, P.-O. New relativistic ANO basis sets for actinide atoms. *Chem. Phys. Lett.* **409**, 295-299 (2005).

24. Douglas, M. & Kroll, N. Quantum electrodynamical corrections to the fine structure of helium. *Ann. Phys.* **155**, 89-155 (1974).

25. Hess, B. Relativistic electronic-structure calculations employing a two-component no-pair formalism with external-field projection operators. *Phys. Rev. A* **33**, 3742-3748 (1986).

26. Bader, R. F. W. Atoms in Molecules: A Quantum Theory. (Oxford University Press, New York, 1990).

27. AIMAll Version 14.11.23. Keith, T. A. (2014) https://aim.tkgristmill.com.
